# Asprecosides A–J, ten new pentacyclic triterpenoid glycosides with cytotoxic activity from the roots of *Ilex asprella*

**DOI:** 10.1007/s13659-025-00499-7

**Published:** 2025-03-13

**Authors:** Yuwei Wu, Baihui Zhang, Wenxian Li, Lihua Peng, Weilin Qiao, Wei Li, De-an Guo

**Affiliations:** 1 School of Pharmaceutical Sciences, Southern Medical University, Guangzhou, 510515 People’s Republic of China; 2 Zhongshan Institute for Drug Discovery, Shanghai Institute of Materia Medica, Chinese Academy of Sciences, Zhongshan, 528400 People’s Republic of China; 3 Zhongshan Zhongzhi Pharmaceutical Group Co. Ltd., Zhongshan, 528400 People’s Republic of China

**Keywords:** *Ilex asprella*, Pentacyclic triterpenoid glycoside, Ursane, Oleanane, Cytotoxic activity

## Abstract

Phytochemical study of the *n*-BuOH extract of *Ilex asprella* resulted in the discovery of ten new pentacyclic triterpenoid glycosides, comprising nine ursane-type glycosides (**1**−**9**) and one oleanane-type glycoside (**10**), along with seven known compounds (**11**−**17**). Compound **1** is the first reported 19,22-epoxy ursane triterpenoid glycoside, whereas **4** and **5** are rare examples of ursane triterpenoid glycosides containing a 28,19-lactone group. The structural characterization of these compounds was achieved using spectroscopic and chemical techniques, as well as single-crystal X-ray analysis. Compounds **7**, **12**, **15**, and **17** exhibited moderate cytotoxic activities against H1975 and HCC827 cancer cells.

**Graphical abstract:**

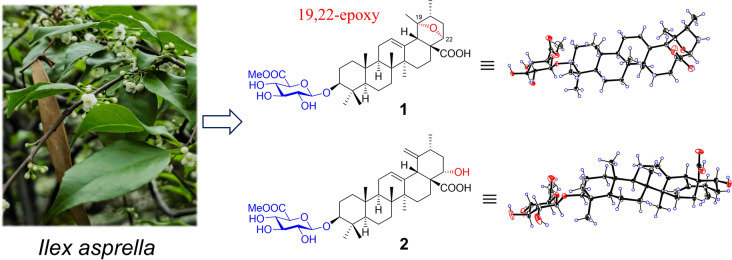

**Supplementary Information:**

The online version contains supplementary material available at 10.1007/s13659-025-00499-7.

## Introduction

Pentacyclic triterpenoid glycosides represent a large class of secondary metabolites predominantly found in higher plants, consisting of diverse triterpenoid aglycones and glycosyl moieties [1, 2]. Common pentacyclic triterpenoid aglycones mainly include oleanane-type, ursane-type, and friedelane-type (6/6/6/6/6 carbocyclic ring system), as well as lupane-type (6/6/6/6/5 carbocyclic ring system) [3]. The structural variability resulting from various functional modifications of triterpenoid aglycones endows these compounds with notable biological properties [4, 5]. In recent years, numerous pentacyclic triterpenoid glycosides exhibiting remarkable bioactivities have been identified, as exemplified by the PTP1B inhibitors gymlatinosides GL2 and GL3 [6], the neuroprotective medicagoside A [7], the *β*-glucuronidase inhibitor astraoleanoside H [8], and the cytotoxic compound ilekudinoside B *n*-butyl ester [9].

**Fig. 1:**
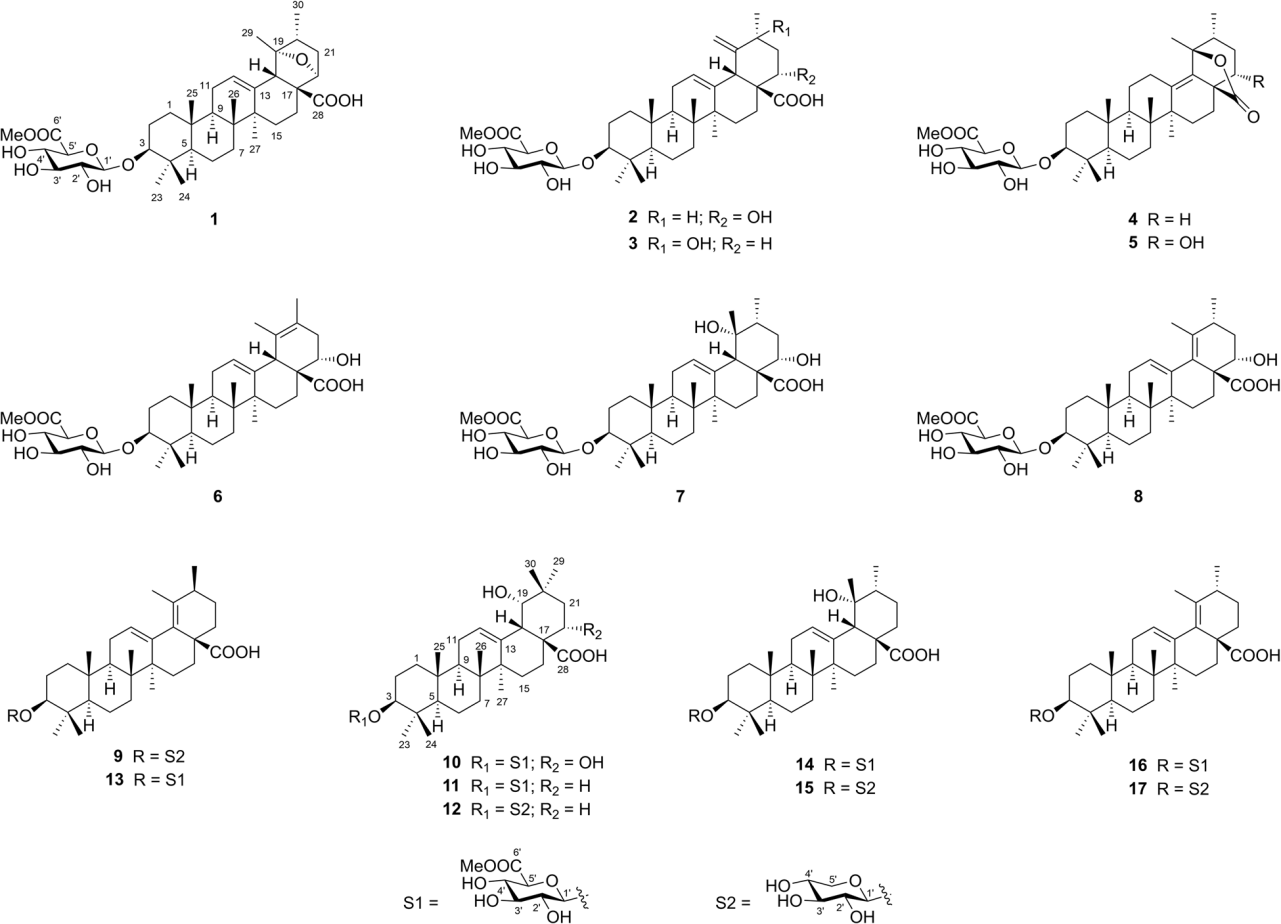
Chemical structures of compounds **1**−**17**

*Ilex asprella* (Hook. & Arn.) Champ. ex Benth (Aquifoliaceae) is a deciduous shrub and predominantly distributed in southern regions of China, particularly in Guangdong, Hunan, and Guangxi provinces [10]. The roots of *I. asprella* are extensively utilized in traditional Chinese medicine to treat of headache, cough, and pharyngitis, etc. [10]. Earlier phytochemical studies indicated that triterpenoids and their glycosides are characteristic components of this plant [11–17], with some exhibiting antiviral and cytotoxic activities. As part of ongoing research to identify structurally diverse terpenoids from medicinal plants [18, 19], nine new ursane triterpenoid glycosides (**1**−**9**), one new oleanane glycoside (**10**), and seven known analogues (**11**−**17**) were isolated from the roots of *I. asprella* (Fig. 1). Compound **1** represents the first 19,22-epoxy ursane triterpenoid glycoside, whereas **4** and **5** are rare ursane triterpenoid glycosides with a 28,19-lactone group. This study presents the isolation, structural characterization, and cytotoxic properties of these triterpenoid glycosides (**1**−**17**).

## Results and discussion

**Fig. 2:**
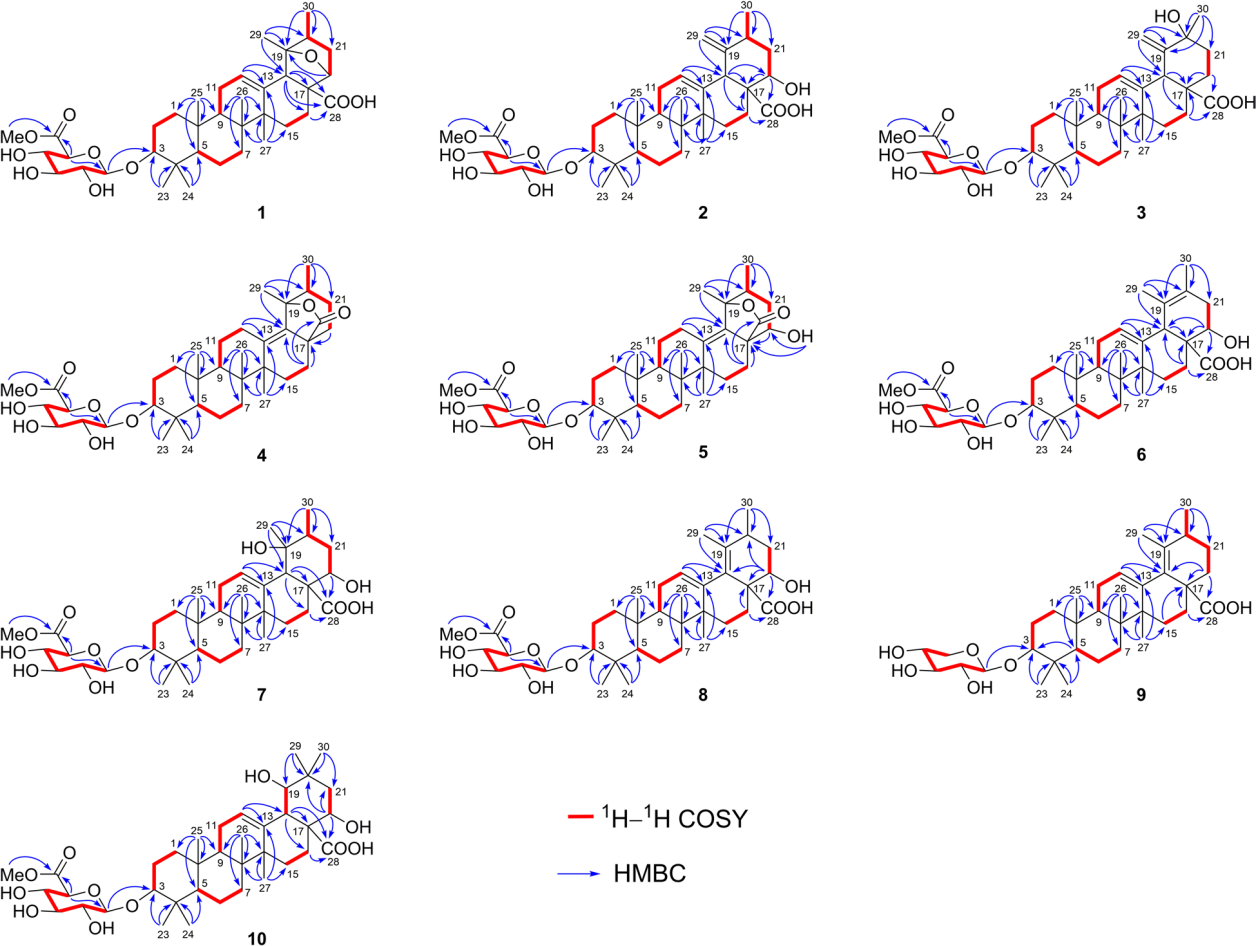
Key ^1^H−^1^H COSY and HMBC correlations of **1**−**10**

Asprecoside A (**1**) was isolated as colorless needles, and its molecular formula C_37_H_56_O_10_ was determined by the HRESIMS ion peak at *m/z* 659.3803 [M − H]^−^ (calcd for C_37_H_55_O_10_^−^, 659.3801). The ^1^H NMR data of **1** (Table 1) exhibited characteristic signals, including six methyl singlets (*δ*_H_ 0.86, 0.93, 0.99, 1.28, 1.30, and 1.32), a methyl doublet [*δ*_H_ 0.91 (d, *J* = 6.7 Hz)], a methoxy group [*δ*_H_ 3.76 (s)], seven oxygenated methines [*δ*_H_ 3.42 (1H, dd, *J* = 11.7, 4.3 Hz), 4.43 (1H, d, *J* = 5.5 Hz), 5.03 (1H, d, *J* = 7.8 Hz), 4.11 (1H, dd, *J* = 9.0, 7.8 Hz), 4.28 (1H, t, *J* = 9.0 Hz), 4.49 (1H, dd, *J* = 9.7, 9.0 Hz), and 4.62 (1H, d, *J* = 9.7 Hz)], and an olefinic proton [*δ*_H_ 5.42 (1H, t, *J* = 3.3 Hz)]. The ^13^C NMR and DEPT data (Table 2) of **1** revealed the presence of 37 carbon resonances, including a carboxyl group (*δ*_C_ 178.7), a methoxycarbonyl group (*δ*_C_ 171.2 and 52.4), a trisubstituted double bond (*δ*_C_ 137.3 and 126.6), an anomeric carbon (*δ*_C_ 107.7), an oxygenated *sp*^3^ tertiary carbon (*δ*_C_ 90.4), five *sp*^3^ quaternary carbon, ten *sp*^3^ methines (six oxygenated), eight *sp*^3^ methylenes, and seven methyls. The above NMR data indicated that the compound exhibited the primary structural characteristics of ursane triterpenoid glycosides and closely resembled ilexasoside A (**14**) [13], a previously reported triterpenoid glycoside, except that the *sp*^3^ methylene (CH_2_-22: *δ*_H_ 2.17 and 2.07; *δ*_C_ 38.5) in **14** was replaced by an oxygenated methine group (*δ*_H_ 4.43; *δ*_C_ 82.6) in **1**, as confirmed by the ^1^H−^1^H COSY correlation between H_2_-21 and H-22. Additionally, two heavily deshielded *O*-bearing carbons (C-19: *δ*_C_ 90.4; C-22: *δ*_C_ 82.6) implied the existence of an epoxy bridge connecting C-19 and C-22, which was further substantiated by the HMBC correlation of H-22/C-19. Comprehensive analysis of 2D NMR data enabled the establishment of the gross structure of **1**, as illustrated in Fig. 2.

**Table 1:** ^1^H NMR data of compounds **1**−**5** (500 MHz, *J* in Hz, *δ* in ppm)

No	**1**^*a*^	**2**^*a*^	**3**^*b*^	**4**^*a*^	**5**^*a*^
1*α*	0.79, m	0.88, m	1.01, m	0.81, m	0.84, m
1*β*	1.45, m	1.42, m	1.64, m	1.57, m	1.59, m
2*α*	2.17, m	2.17, m	1.80, m	2.20, m	2.19, m
2*β*	1.88, m	1.86, m	1.69, m	1.90, m	1.91, m
3	3.42, dd (11.7, 4.3)	3.42, dd (11.7, 4.3)	3.17, dd (11.6, 4.5)	3.40, dd (11.8, 4.5)	3.39, dd (11.7, 4.3)
5	0.81, m	0.83, br d (10.7)	0.81, br d (10.9)	0.76, m	0.77, br d (11.8)
6*α*	1.49, m	1.54, m	1.57, m	1.51, m	1.51, m
6*β*	1.25, m	1.29, m	1.45, m	1.29, m	1.24, m
7*α*	1.39, m	1.35, m	1.51, m	1.35, m	1.23, m
7*β*	1.49, m	1.53, m	1.34, m	1.44, m	1.45, m
9	1.58, dd (9.1, 8.3)	1.74, m	1.72, m	1.38, m	1.49, m
11	1.90, m	*α* 1.97, m	1.99, m	*α* 1.41, m	*α* 1.42, m
		*β* 1.90, m		*β* 1.18, m	*β* 1.32, m
12	5.42, t (3.3)	5.58, t (3.3)	5.34, t (3.4)	*α* 1.87, m	*α* 2.09, td (14.7, 5.2)
				*β* 2.58, m	*β* 2.78, br d (14.7)
15*α*	1.19, m	1.27, m	1.05, m	1.20, m	1.60, m
15*β*	1.68, m	2.53, td (13.2, 3.0)	1.83, m	1.72, m	1.66, m
16*α*	2.24, m	1.75, m	1.61, m	1.59, m	2.24, m
16*β*	2.48, m	2.73, br d (13.2)	1.73, m	2.03, m	2.28, m
18	3.31, s	3.98, br s	3.85, br s		
20	1.91, m	2.28, m		2.05, m	2.20 m
21*α*	1.37, m	1.80, m	1.61, m	1.34, m	1.78, m
21*β*	2.30, dd (12.4, 8.1)	2.10, dt (12.3, 3.9)	1.66, m	2.02, m	2.32, m
22	4.43, d (5.5)	4.51, dd (11.7, 3.9)	*α* 1.55, m	*α* 1.54, m	4.18, br dd (4.6, 3.1)
			*β* 2.21, td (13.0, 5.3)	*β* 1.71, m	
23	1.32, s	1.32, s	1.05, s	1.33, s	1.32, s
24	0.99, s	0.98, s	0.86, s	0.98, s	0.99, s
25	0.86, s	0.82, s	0.98, s	0.78, s	0.83, s
26	0.93, s	1.05, s	0.86, s	0.77, s	0.87, s
27	1.28, s	1.37, s	1.13, s	1.17, s	1.49, s
29	1.30, s	a 5.27, s	a 5.28, s	1.67, s	1.79, s
		b 5.13, s	b 5.09, s		
30	0.91, d (6.7)	1.18, d (6.4)	1.40, s	0.85, d (7.1)	1.33, d (7.1)
1′	5.03, d (7.8)	5.02, d (7.8)	4.39, d (7.8)	5.04, d (7.8)	5.04, d (7.8)
2′	4.11, dd (9.0, 7.8)	4.10, dd (9.0, 7.8)	3.23, dd (9.2, 7.8)	4.12 dd (9.0, 7.8)	4.12, dd (9.0, 7.8)
3′	4.28, t (9.0)	4.28, t (9.0)	3.35, t (9.2)	4.29, t (9.0)	4.30, t (9.0)
4′	4.49, dd (9.7, 9.0)	4.49, dd (9.7, 9.0)	3.51, dd (9.8, 9.2)	4.51, dd (9.7, 9.0)	4.51, dd (9.7, 9.0)
5′	4.62, d (9.7)	4.62, d (9.7)	3.83, d (9.8)	4.63, d (9.7)	4.64, d (9.7)
OMe-6′	3.76, s	3.76, s	3.77, s	3.76, s	3.76, s
OH-22					6.91, d (4.6)

^*a*^In pyridine-*d*_5_, ^*b*^in CD_3_OD

**Table 2:** ^13^C NMR data (125 MHz) of compounds **1**–**10**

No	**1**^*a*^	**2**^*a*^	**3**^*b*^	**4**^*a*^	**5**^*a*^	**6**^*a*^	**7**^*a*^	**8**^*a*^	**9**^*b*^	**10**^*a*^
1	39.3	39.1	39.8	39.5	39.5	39.4	39.1	39.6	40.3	38.8
2	27.0	27.0	27.0	27.2	27.1	27.1	27.0	27.1	27.2	26.9
3	89.5	89.5	91.1	89.4	89.4	89.5	89.6	89.5	90.6	89.5
4	39.9	39.9	40.2	40.0	40.0	39.9	39.9	39.9	40.2	39.9
5	56.3	56.3	57.1	56.2	56.1	56.3	56.2	56.3	57.2	56.2
6	18.8	18.8	19.3	18.8	18.8	18.7	19.0	18.8	19.2	19.0
7	34.5	33.7	34.2	35.4	35.9	34.6	33.8	35.7	35.8	33.5
8	39.6	40.1	40.5	42.7	42.4	40.1	40.8	39.9	40.2	40.5
9	48.5	48.3	48.9	52.3	51.8	48.5	48.0	48.6	49.2	48.5
10	37.2	37.3	38.0	37.7	37.7	37.2	37.3	37.1	37.8	37.4
11	24.0	24.2	24.6	22.4	22.1	23.9	24.3	23.6	24.2	24.5
12	126.6	128.8	129.3	26.6	27.7	127.6	128.5	125.4	127.2	124.1
13	137.3	138.2	138.3	137.1	141.3	138.9	140.2	141.1	139.9	144.8
14	42.2	43.8	43.6	43.5	43.5	44.7	43.0	46.4	45.7	42.7
15	27.0	29.1	29.6	28.3	28.6	28.5	29.3	29.5	29.8	29.2
16	28.9	19.7	26.3	27.0	23.6	18.7	19.7	27.5	35.9	21.1
17	59.8	55.5	49.6	48.9	55.2	53.4	54.9	57.4	49.5	53.1
18	55.5	53.4	48.7	133.5	130.5	54.1	55.8	135.0	134.5	46.4
19	90.4	153.5	154.1	91.4	92.2	129.5	72.9	133.1	137.0	81.5
20	44.1	35.8	71.0	40.2	39.6	123.4	41.0	35.1	35.7	36.8
21	37.1	39.6	35.6	27.4	36.8	39.0	36.3	38.0	27.5	38.3
22	82.6	74.7	32.7	32.5	72.8	72.5	75.4	72.3	32.4	72.0
23	28.5	28.5	28.5	28.4	28.4	28.6	28.5	28.6	28.6	28.5
24	17.3	17.3	17.0	17.2	17.2	17.4	17.2	17.4	17.1	17.2
25	16.4	15.9	16.1	17.3	17.2	16.3	15.8	16.6	16.7	15.7
26	17.7	17.5	17.6	19.2	19.0	18.5	17.4	18.8	18.6	17.7
27	21.9	27.1	26.5	25.0	21.9	22.7	25.4	22.6	22.3	25.6
28	178.7	179.3	181.3	178.5	178.2	178.9	180.1	178.7	180.5	180.9
29	16.6	110.9	113.3	23.3	23.4	17.5	27.3	17.5	19.8	26.3
30	19.9	19.7	29.2	15.8	18.5	20.8	17.0	21.4	19.1	29.5
1′	107.7	107.7	107.1	107.8	107.7	107.7	107.7	107.7	107.4	107.7
2′	75.8	75.8	75.3	75.8	75.8	75.8	75.8	75.8	75.4	75.8
3′	78.2	78.3	77.5	78.3	78.3	78.3	78.3	78.3	78.0	78.2
4′	73.5	73.5	73.2	73.6	73.6	73.5	73.6	73.5	71.2	73.6
5′	77.5	77.6	76.6	77.7	77.6	77.6	77.6	77.6	66.7	77.6
6′	171.2	171.2	171.4	171.3	171.3	171.2	171.2	171.2		171.3
OMe	52.4	52.4	52.8	52.4	52.4	52.4	52.4	52.4		52.4

^*a*^In pyridine-*d*_5_, ^*b*^in CD_3_OD

**Fig. 3:**
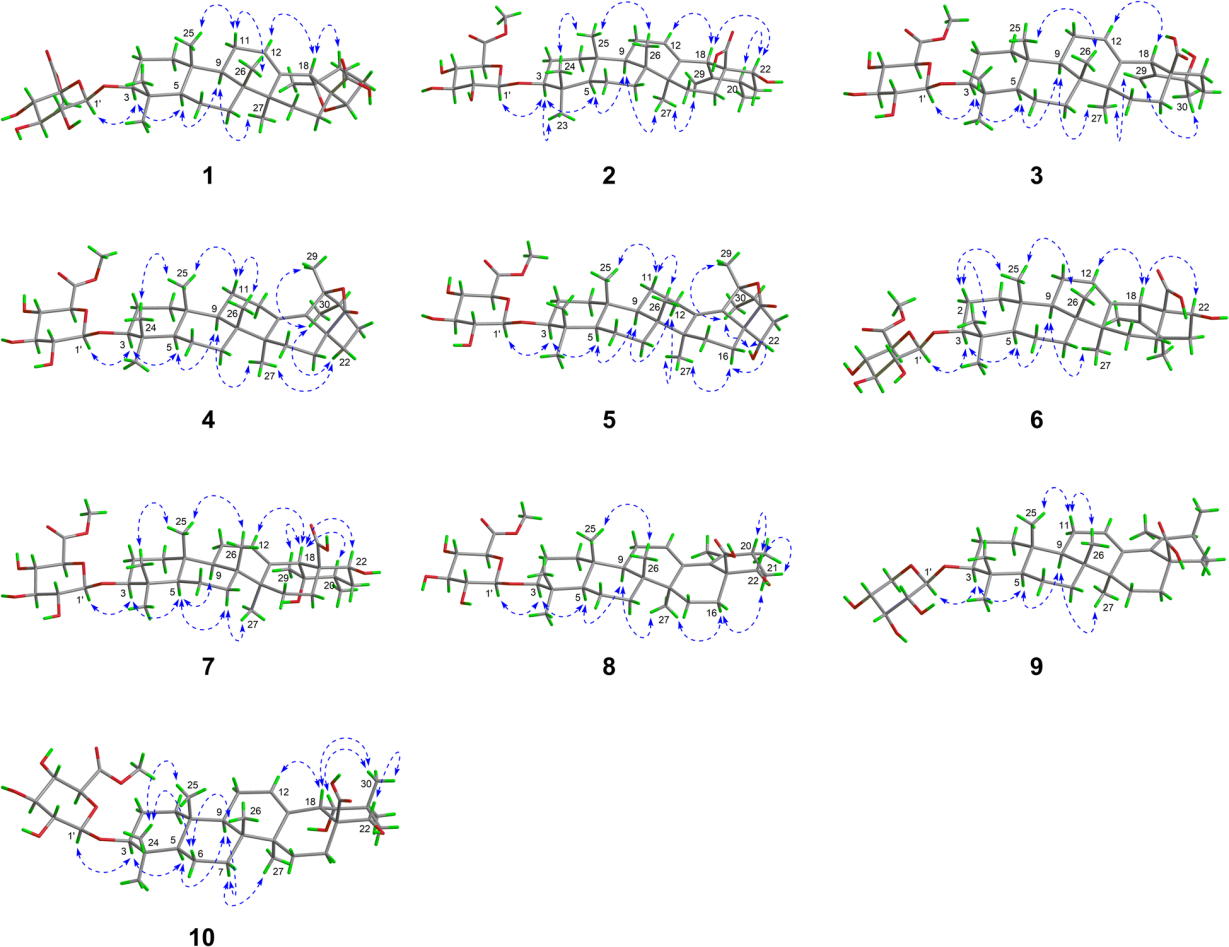
Key NOESY correlations of **1**−**10**

**Fig. 4:**
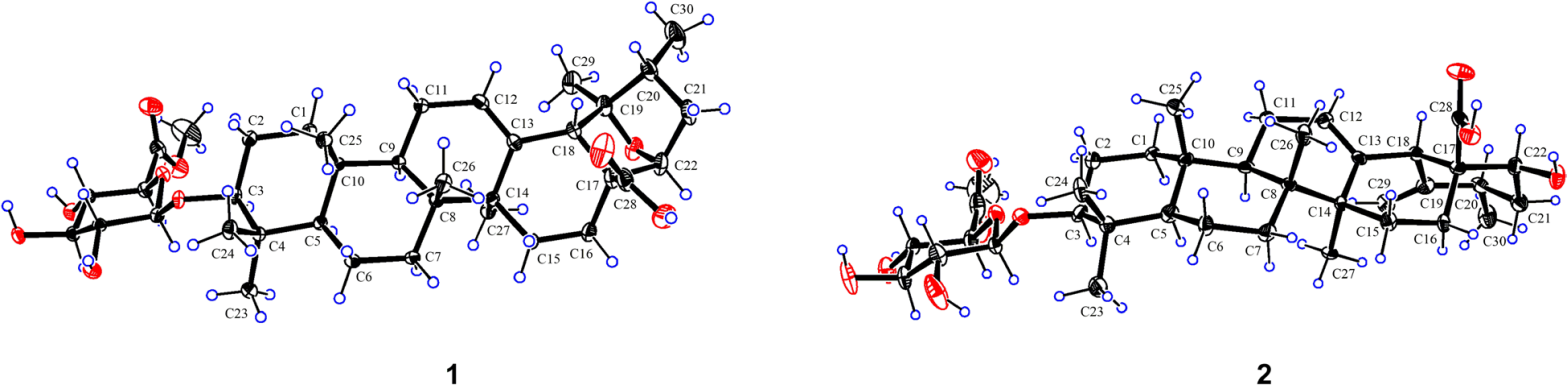
X-ray crystal structures of compounds **1** and **2**

The relative configuration of **1** was partially elucidated through the analysis of NOESY spectra and coupling constant data. The *β*-configuration of the glycosyl group was confirmed based on the coupling constant of its anomeric proton (d, *J* = 7.8 Hz, H-1′). The observed NOESY correlations (Fig. 3) of H-3/H-5, H-5/H-9, H-9/H_3_-27 revealed that these protons, together with Me-27, were cofacial and were arbitrarily assigned *α*-orientations. Consequently, the correlations of H-11*β*/H_3_-25 and H_3_-26 indicated that Me-25 and Me-26 were *β*-oriented. Furthermore, the *β*-orientations of H-18 and H-20 were supported by the NOE correlations of H-18/H-12 and H-20. Nevertheless, due to the absence of useful NOESY signals, the configurations of the other stereocenters could not be determined. Ultimately, the complete structure of **1**, including its absolute configuration, was confirmed through single-crystal X-ray diffraction analysis (Fig. 4), with a Flack parameter of − 0.02(3).

Asprecoside B (**2**) was identified with a molecular formula of C_37_H_56_O_10_, based on the HRESIMS data. A comparison of the NMR data of **2** (Tables 1 and 2) with those of **14** revealed key distinctions: the presence of an additional terminal double bond [*δ*_H_ 5.27 and 5.13 (each 1H, s); *δ*_C_ 153.5 and 110.9] in place of the oxygenated *sp*^3^ tertiary carbon and a methyl group in **14**, along with an oxymethine [*δ*_H_ 4.51 (1H, dd, *J* = 11.7, 3.9 Hz); *δ*_C_ 74.7] in **2** instead of the methylene in **14**. The location of Δ^19(29)^ double bond was confirmed by HMBC, which showed correlations (Fig. 2) from the olefinic protons (*δ*_H_ 5.27 and 5.13, H_2_-29) to C-18, C-19, and C-20; as well as from H_3_-30 to C-19. Furthermore, the position of the hydroxy group at C-22 was verified through the HMBC correlations of H-22/C-17 and C-28 and H-18/C-22, alongside the ^1^H − ^1^H COSY correlation of H-22/H_2_-21. The relative configuration of **2** was determined based on analysis of its NOE data. In particular, the NOESY correlations (Fig. 3) of H-18/H-22 and H-22/H-20 indicated the *β*-configurations of H-20 and H-22. The structure of **2** was further confirmed by single-crystal X-ray diffraction (Fig. 4).

Asprecoside C (**3**) shared the same molecular formula (C_37_H_56_O_10_) as that of **2**, suggesting that they were structural isomers. The 1D NMR spectra of **3** revealed similar structural characteristics found in **2**, with the key difference being the hydroxy group relocating from C-22 in **2** to C-20 in **3**. This was supported by the HMBC correlations (Fig. 2) from a methyl singlet (*δ*_H_ 1.40, H_3_-30) to an oxygenated *sp*^3^ tertiary carbon (*δ*_C_ 71.0, C-20), as well as the ^1^H−^1^H COSY correlation of H_2_-21/H_2_-22. The stereochemistry of **3** was assigned to be the same as that of **2** by comparison of their NOE data (Fig. 3).

The molecular formula of asprecoside D (**4**) was determined to be C_37_H_56_O_9_ based on its HRESIMS data. The ^1^H and ^13^C NMR data (Tables 1 and 2) of **4** exhibited a close resemblance to those of **14**, except for the presence of a tetrasubstituted double bond instead of the trisubstituted double bond in **14**. This suggested that Δ^12(13)^ in **14** shifted to Δ^13(18)^ in **4**, which was validated by the HMBC corrections (Fig. 2) from H_2_-12 and H_3_-27 to a hydrogen-free *sp*^2^ carbon (*δ*_C_ 137.1, C-13) and from H_2_-12 and H_3_-29 to the other hydrogen-free *sp*^2^ carbon (*δ*_C_ 133.5, C-18). Additionally, the significant downfield shift of C-19 (*δ*_C_ 91.4 in **4**; *δ*_C_ 72.6 in **14**) and slight upfield shift of C-28 (*δ*_C_ 178.5 in **4**; *δ*_C_ 180.8 in **14**) suggested that C-19 and C-28 were linked via an *O* atom, leading to the formation of a five-membered lactone ring. The relative stereochemistry of **4** was established by analysis of its NOESY correlations (Fig. 3).

Asprecoside E (**5**) exhibited a molecular formula of C_37_H_56_O_10_, containing one additional *O* atom compared to **4**. The 1D NMR spectra of **5** resembled those of **4**, except that the *sp*^3^ methylene in **4** was substituted by an oxymethine [*δ*_H_ 4.18 (1H, br dd, *J* = 4.6, 3.1 Hz); *δ*_C_ 72.8], indicating that **5** was a hydroxylated analogue of **4**. HMBC correlations (Fig. 2) revealed that the hydroxy group was attached at C-22, as evidenced by signals from OH-22 [*δ*_H_ 6.91, 1H, d (*J* = 4.6 Hz)] to C-17 and C-22. Especially, the *α*-orientation of OH-22 was assigned by the NOE correlations (Fig. 3) of OH-22/H_3_-30 and H-16*α* as well as H-16*α*/H_3_-27.

The molecular formula of asprecoside F (**6**), C_37_H_56_O_10_, was determined to be identical to that of **2** through HRESIMS analysis ([M − H]^−^ *m/z* 659.3805). When comparing its 1D NMR data (Tables 2 and 3) of with those of **2**, the only structural difference between these two compounds was that Δ^19(29)^ double bond in **2** migrated to Δ^19(20)^ in **6**. This was confirmed by the HMBC correlations (Fig. 2) from a vinyl methyl (*δ*_H_ 1.73, H_3_-29) to C-18, C-19, and C-20 and from the other vinyl methyl (*δ*_H_ 1.70, H_3_-30) to C-19, C-20, and C-21. Comparison of their NOE data demonstrated that the relative stereochemistry of **6** aligned with that of **2**.

**Table 3:** ^1^H NMR data of compounds **6**−**10** (500 MHz, *J* in Hz, *δ* in ppm)

No	**6**^*a*^	**7**^*a*^	**8**^*a*^	**9**^*b*^	**10**^*a*^
1*α*	0.79, m	0.85, m	0.82, m	1.03, m	0.86, m
1*β*	1.46, m	1.43, m	1.50, m	1.73, m	1.39, m
2*α*	2.15, m	2.14, m	2.16, m	1.84, m	2.13, br d (11.4)
2*β*	1.87, m	1.86, m	1.85, m	1.68, m	1.86, m
3	3.40, dd (11.7, 4.3)	3.37, dd (11.7, 4.2)	3.42, dd (11.7, 4.3)	3.15, dd (11.8, 4.1)	3.36, dd (10.8, 2.8)
5	0.80, m	0.84, m	0.83, m	0.81, br d (11.2)	0.81, m
6*α*	1.49, m	1.50, m	1.49, m	1.55, m	1.50, m
6*β*	1.27, m	1.34, m	1.30, m	1.39, m	1.33, m
7*α*	1.58, m	1.63, m	1.62, m	1.58, m	1.53, m
7*β*	1.44, m	1.42, m	1.51, m	1.50, m	1.37, m
9	1.48, m	1.78, m	1.47, m	1.44, m	1.81, m
11	1.84, m	2.00, m	1.91, m	1.96, m	1.98, m
12	5.66, t (3.3)	5.59, t (3.2)	5.71, t (3.3)	5.38, t (3.8)	5.57, t (3.7)
15*α*	1.35, m	1.39, m	1.45, m	1.17, dt (13.7, 3.8)	1.37, m
15*β*	2.71, m	2.51, m	2.69, td (13.2, 4.6)	1.90, td (13.7, 3.8)	2.29, br t (12.1)
16*α*	2.04, m	3.02, td (13.1, 6.4)	2.18, td (13.0, 4.3)	1.38, m	2.75, m
16*β*	2.73, m	2.78, br d (13.1)	2.91, br d (13.0)	2.17, m	2.83, m
18	3.74, s	3.08, s			3.70, br s
19					3.63, br s
20		1.72, m	2.58, m	2.19, m	
21*α*	2.67, dd (17.0, 10.7)	1.90, m	1.99, m	1.36, m	2.49, t (12.0)
21*β*	2.46, dd (17.0, 5.4)	2.49, m	2.29, m	1.83, m	1.73, m
22	4.62, dd (10.7, 5.4)	4.44, dd (11.6, 4.1)	4.83, dd (9.9, 3.2)	*α* 1.63, m	4.62, dd (12.0, 3.8)
				*β* 1.81, m	
23	1.31, s	1.31, s	1.33, s	1.06, s	1.31, s
24	0.97, s	0.98, s	0.98, s	0.86, s	0.98, s
25	0.83, s	0.83, s	0.84, s	1.01, s	0.83, s
26	1.06, s	1.12, s	1.12, s	0.94, s	1.06, s
27	1.25, s	1.80, s	1.26, s	1.00, s	1.72, s
29	1.73, s	1.46, s	1.96, s	1.74, s	1.22, s
30	1.70, s	1.19, d (6.6)	1.28, d (7.1)	1.10, d (7.0)	1.26, s
1′	5.02, d (7.8)	5.01, d (7.8)	5.03, d (7.8)	4.27, d (7.5)	5.02, d (7.7)
2′	4.10, dd (9.0, 7.8)	4.10, dd (8.7, 7.8)	4.11, dd (9.0, 7.8)	3.18, dd (8.9, 7.5)	4.11, dd (9.0, 7.7)
3′	4.28, t (9.0)	4.28, t (8.7)	4.28, t (9.0)	3.28, t (8.9)	4.29, t (9.0)
4′	4.49, dd (9.7, 9.0)	4.50, dd (9.7, 8.7)	4.50, dd (9.7, 9.0)	3.46, ddd (10.1, 8.9, 5.3)	4.49, t (9.6, 9.0)
5′	4.62, d (9.7)	4.62, d (9.7)	4.63, d (9.7)	*α* 3.19, dd (11.4, 10.1)	4.64, d (9.6)
				*β* 3.82, dd (11.4, 5.3)	
OMe-6′	3.76, s	3.76, s	3.77, s		3.74, s

^*a*^In pyridine-*d*_5_, ^*b*^in CD_3_OD

Asprecoside G (**7**) was assigned a molecular formula of C_37_H_58_O_11_, as determined based on HRESIMS data at *m/z* 677.3905 [M − H]^−^ (calcd for C_37_H_57_O_11_^−^, 677.3906). Comparing the NMR data (Tables 2 and 3) of **7** with those of **14** demonstrated their structural resemblance. The primary distinction was the substitution of the *sp*^3^ methylene (CH_2_-22) in **14** with an oxymethine [*δ*_H_ 4.44 (1H, dd, *J* = 11.6, 4.1 Hz); *δ*_C_ 75.4] in **7**, indicating that **7** was a 22-hydroxylated derivative of **14**. This was corroborated by the analysis of 2D NMR data (Fig. 2), especially the ^1^H−^1^H COSY correlation of the H_2_-21/H-22. The *β*-configuration of H-22 was established based on the NOE correlation between H-18 and H-22 (Fig. 3).

Asprecoside H (**8**) showed a molecular formula of C_37_H_56_O_10_, as proved by the HRESIMS data. Its 1D NMR data resembled those of **7**, except for the presence of a tetrasubstituted double bond (*δ*_C_ 135.0 and 133.1) in **8** replacing the *sp*^3^ methine at C-18 (*δ*_C_ 55.8) and the oxygenated *sp*^3^ tertiary carbon at C-19 (*δ*_C_ 72.9) in **7**. This suggested that **8** was a C-18 − C-19 dehydrated derivative of **7**, and was further confirmed by the HMBC correlations (Fig. 2) from a vinyl methyl (*δ*_H_ 1.96, H_3_-29) to C-18 and C-19. The similar NOE correlations observed in **8** and **7** (Fig. 3) indicated that **8** shared the same relative stereochemistry as **7**.

The molecular formula of asprecoside I (**9**) was determined as C_35_H_54_O_7_ by its HRESIMS data at *m/z* 585.3799 [M − H]^−^ (C_35_H_53_O_7_^−^, 585.3797). Comparison of its NMR data (Tables 2 and 3) with those of known compound (**13**) [20] revealed that they shared the same aglycone moiety but a different sugar unit. In the ^1^H and ^13^C NMR spectra of **9**, the chemical shifts [*δ*_H_ 4.27 (1H, d, *J* = 7.5 Hz), 3.82 (1H, dd, *J* = 11.4, 5.3 Hz), 3.46 (1H, ddd, *J* = 10.1, 8.9, 5.3 Hz), 3.28 (1H, t, *J* = 8.9 Hz), 3.19 (1H, dd, *J* = 11.4, 10.1 Hz), and 3.18 (1H, dd, *J* = 8.9, 7.5 Hz); *δ*_C_ 107.4, 78.0, 75.4, 71.2, and 66.7] indicated the presence of a *β*-xylosyl group, which was attached to C-3 of the aglycone by the HMBC correlation (Fig. 2) from H-1′ (*δ*_H_ 4.27) to C-3 (*δ*_C_ 90.6). Compound **9** shared the same relative stereochemistry with that of **13** regarding the aglycone moiety, by comparing their 1D NMR and NOESY data, especially the NOE correlations of H-1/H-3, H-3/H-5, H-5/H-9, H-9/H_3_-27, H_3_-25/H-11*β*, H-11*β*/H_3_-26. The relative configuration of the chiral center C-20 was specifically determined by quantum chemical calculations of the 1D NMR chemical shifts of aglycone moiety in **9**, for both the 20S*-isomer and 20R*-isomer. The improved probability DP4 + analysis of the experimental and calculated 1D NMR data yielded the final score, with 20S*-isomer (100.00%) showing an absolute advantage over 20R*-isomer (0.00%), suggesting a 20S*-configuration for compound **9**.

Asprecoside J (**10**) exhibited an [M − H]^−^ ion peak at *m/z* 677.3898 (C_37_H_57_O_11_^−^, 677.3906), indicating a molecular formula of C_37_H_58_O_11_. The NMR data (Tables 2 and 3) of **10** bore a resemblance to those of known oleanane glycoside (**11**) [21]. The key difference was the substitution of the sp^3^ methylene in **11** by an oxymethine [*δ*_H_ 4.62 (1H, dd, *J* = 12.0, 3.8 Hz); *δ*_C_ 72.0] in **10**, identifying **10** as a hydroxylated derivative of **11**. The HMBC correlations (Fig. 2) of H-22/C-20, C-21, and C-28, along with ^1^H−^1^H COSY correlation of H-22/H_2_-21 confirmed the position of the hydroxyl group at C-22. The relative stereochemistry of **10** was elucidated through comparison of its 1D NMR data and NOE correlations with those of **11**. Notably, the *β*-orientation of H-22 was verified by the observed NOE correlations of H-18/H_3_-30 and H_3_-30/H-22.

To ascertain the absolute configurations of the glycosyl moiety in **1**−**10**, HPLC sugar analysis following acid hydrolysis and thiazolidine thiocarbamoyl derivatization was conducted [22]. The results demonstrated the occurrence of a D-xylosyl group in **9** and a D-glucuronosyl group in **1**−**8** and **10**.

The known compounds were identified as 19*α*-hydroxy oleanolic acid 3-*O*-*β*-D-glucuronopyranoside-6′-*O*-methyl ester (**11**) [21], ilexoside A (**12**) [23], 3*β*-hydroxy-20-*epi*-ursa-12,18-dien-28-oic acid 3-*O*-*β*-D-glucuronopyranoside-6′-*O*-methyl ester (**13**) [20], ilexasoside A (**14**) [13], ilexoside B (**15**) [24], 3*β*-hydroxyursa-12,18-dien-28-oic acid 3-*O*-*β*-D-glucuronopyranoside-6′-*O*-methyl ester (**16**) [25], and ilexasprellanoside A (**17**) [14], through comparison of their NMR data with reported values.

Compounds **1**−**17** exhibited similar structures, especially the triterpenoid aglycone moiety with diverse functional groups. Based on the well-known triterpenoids, oleanolic acid and ursolic acid, the proposed biosynthetic pathways and structural relationships of **1**−**17** are shown in Additional file 1: Fig S1.

The cytotoxic activities of **1**−**17** against H1975 and HCC827 cells were evaluated using the CCK-8 method, with gefitinib serving as the positive control. Apart from four compounds (**7**, **12**, **15**, and **17**) showing moderate cytotoxicity with IC_50_ values ranging from 10.85 to 48.41 *μ*M, the other compounds did not display obvious activity (IC_50_ > 50 *μ*M). Among these, compound **12** was identified as the most potent, exhibiting cytotoxicity against H1975 cells (IC_50_ = 10.85 ± 0.34 *μ*M).

## Experimental section

### General experimental procedures

Optical rotations were determined using a Rudolph Autopol I automatic polarimeter, and X-ray crystal crystallographic data were collected on an Agilent Xcalibur Nova X-ray diffractometer. Melting points were measured on an X-4 melting instrument. UV and IR spectra were recorded using a Shimadzu UV-2600i spectrophotometer and a Bruker Tensor 37 infrared spectrophotometer, respectively. 1D and 2D NMR spectra acquired on a Bruker AM-500 spectrometer at 25 °C. HR-ESI–MS was performed using a Waters Micromass Q-TOF spectrometer. Semipreparative HPLC was performed on a Shimadzu LC-40B XR liquid chromatograph equipped with a YMC-Pack ODS-A column (250 mm × 10 mm, 5 μm). Silica gel (200 − 300 mesh, Qingdao Marine Chemical, Inc, Qingdao, China), Sephadex LH-20 (GE Healthcare Bio-Sciences AB, Sweden), and reversed-phase C_18_ (RP-C_18_) silica gel (50 *μ*m, Quebec City Canada) were employed for column chromatography (CC).

### Plant material

The roots of *I. asprella* were collected in April 2023 from Hezhou City, Guangxi Zhuang Autonomous Region, China. The plants were authenticated by one of the authors (W. Li) and a voucher specimen (No. GM202304) has been deposited in the Zhongshan Institute for Drug Discovery.

### Extraction and isolation

The dried roots of *I. asprella* (20 kg) were crushed and subjected to reflux extraction using 65% EtOH for three times (3 × 100 L, 2 h each), producing a crude extract (1.6 kg). The extract was suspended in hot water (2.0 L) and successively partitioned with EtOAc (3 × 3.0 L) and *n*-BuOH (3 × 3.0 L), yielding EtOAc (105 g) and *n*-BuOH (303 g) fractions.

The *n*-BuOH extract was chromatographed on a silica gel column using a gradient of petroleum ether (PE)/EtOAc/MeOH solvent system (5:1:0 → 0:0:1), yielding six fractions (Frs. A − F). Fr. C (24.8 g) was processed on an ODS gel column (MeOH/H_2_O, 3:7 → 1:0) to afford four sub-fractions (Frs. C1 − C4). Fr. C2 (2.8 g) was separated on a Sephadex LH-20 column (MeOH) and further purified by semipreparative RP-HPLC with a YMC-park ODS-A column (CH_3_CN/H_2_O, 45:55, 3 mL/min) to obtain compounds **15** (17.5 mg, *t*_R_ 38 min), **9** (16.6 mg, *t*_R_ 41 min), **13** (2.7 mg, *t*_R_ 43 min), and **17** (7.5 mg, *t*_R_ 33 min). Fr. D (33.5 g) was subjected to repeated silica gel column chromatography with CH_2_Cl_2_/MeOH (100:1 → 0:1), affording five fractions (Frs. D1–D5). Fr. D4 (778.2 mg) was further separated using a Sephadex LH-20 column (MeOH), resulting in five subfractions (Frs. D4a–Fr. D4e). Fr. D4b (94.1 mg) was purified via HPLC (MeOH/H_2_O, 77:23, 2 mL/min) to yield **5** (1.9 mg, *t*_R_ 28 min), **11** (7.2 mg, *t*_R_ 34 min), and **12** (10.7 mg, *t*_R_ 35 min). Similarly, Fr. D4c (39.1 mg) was processed using HPLC (CH_3_CN/H_2_O, 43:57, 2 mL/min) to yield **1** (19.7 mg, *t*_R_ 50 min). Fr. D4e (88.5 mg) was also purified using HPLC (CH_3_CN/H_2_O, 52:48, 2 mL/min) to afford **3** (4.1 mg, *t*_R_ 12 min), **14** (15.5 mg, *t*_R_ 15 min), and **16** (15.7 mg, *t*_R_ 17 min). Chromatography of Fr. D5 on an ODS gel column (MeOH/H_2_O, 1:1 → 1:0) produced six fractions (Fr. D5a–D5f). Fr. D5b (17.5 mg) was further refined using HPLC (CH_3_CN/H_2_O, 30:70, 2 mL/min) to isolate **7** (5.7 mg, *t*_R_ 64 min) and **10** (9.6 mg, *t*_R_ 74min). Compounds **8** (8.0 mg, *t*_R_ 18 min), **4** (5.6 mg, *t*_R_ 21 min), and **6** (12.7 mg, *t*_R_ 22 min) were purified from Fr. D5c (50.5 mg) by HPLC (CH_3_CN/H_2_O, 45:55, 2 mL/min), and **2** (11.7 mg, *t*_R_ 55 min) was obtained from Fr. D5f (24.2 mg) by HPLC (CH_3_CN/H_2_O, 38:62, 2 mL/min).

### Spectroscopic data of compounds

#### Asprecoside A (1)

Colorless needles; [*α*]_D_^20^ −16 (*c* 0.1, CH_3_CN); IR (KBr) *ν*_max_ 3417, 2950, 1738, 1444, 1374, 1238, 1092, 1045, 1027, 982 cm^−1^; ^1^H and ^13^C NMR data (Tables 1 and 2); HRESIMS *m/z* 659.3803 [M − H]^−^ (calcd for C_37_H_55_O_10_^−^, 659.3801).

#### Asprecoside B (2)

Colorless needles; [*α*]_D_^20^ −12 (*c* 0.1, CH_3_CN); IR (KBr) *ν*_max_ 3424, 2925, 1738, 1458, 1376, 1168, 1051, 1025, 910 cm^−1^; ^1^H and ^13^C NMR data (Tables 1 and 2); HRESIMS *m/z* 659.3799 [M − H]^−^ (calcd for C_37_H_55_O_10_^−^, 659.3801).

#### Asprecoside C (3)

White amorphous powder; [*α*]_D_^20^ −21 (*c* 0.1, MeOH); IR (KBr) *ν*_max_ 3434, 2948, 1737, 1452, 1387, 1227, 1171, 1046 cm^−1^; ^1^H and ^13^C NMR data (Tables 1 and 2); HRESIMS *m/z* 659.3816 [M − H]^−^ (calcd for C_37_H_55_O_10_^−^, 659.3801).

#### Asprecoside D (4)

White amorphous powder; [*α*]_D_^20^ −11 (*c* 0.1, MeOH); IR (KBr) *ν*_max_ 3424, 2943, 1747, 1456, 1373, 1167, 1053 cm^−1^; ^1^H and ^13^C NMR data (Tables 1 and 2); HRESIMS *m/z* 689.3907 [M + HCOO]^−^ (calcd for C_38_H_57_O_11_^−^, 689.3906).

#### Asprecoside E (5)

White amorphous powder; [*α*]_D_^20^ −14 (*c* 0.1, MeOH); IR (KBr) *ν*_max_ 3441, 2925, 1743, 1456, 1376, 1049 cm^−1^; ^1^H and ^13^C NMR data (Tables 1 and 2); HRESIMS *m/z* 705.3857 [M + HCOO]^−^ (calcd for C_38_H_57_O_12_^−^ 705.3856).

#### Asprecoside F (6)

White amorphous powder; [*α*]_D_^20^ −25 (*c* 0.1, CH_3_CN); IR (KBr) *ν*_max_ 3423, 2925, 1739, 1441,1374, 1244, 1164, 1046, 1025, 914 cm^−1^; ^1^H and ^13^C NMR data (Tables 2 and 3); HRESIMS *m/z* 659.3805 [M − H]^−^ (calcd for C_37_H_55_O_10_^−^, 659.3801).

#### Asprecoside G (7)

White amorphous powder; [*α*]_D_^20^ −69 (*c* 0.1, CH_3_CN); IR (KBr) *ν*_max_ 3423, 2925, 1738, 1443, 1375, 1249, 1166, 1048, 1026, 982 cm^−1^; ^1^H and ^13^C NMR data (Tables 2 and 3); HRESIMS *m/z* 677.3905 [M − H]^−^ (calcd for C_37_H_57_O_11_^−^, 677.3906).

#### Asprecoside H (8)

White amorphous powder; [*α*]_D_^20^ +18 (*c* 0.1, CH_3_CN); UV (CH_3_CN) *λ*_max_ (log ε) 225 (3.97) nm; IR (KBr) *ν*_max_ 3417, 2924, 1738, 1442, 1372, 1238, 1166, 1084, 1044 cm^−1^; ^1^H and ^13^C NMR data (Tables 2 and 3); HRESIMS *m/z* 659.3817 [M − H]^−^ (calcd for C_37_H_55_O_10_^−^, 659.3801).

#### Asprecoside I (9)

White amorphous powder; [*α*]_D_^20^ +14 (*c* 0.1, CH_3_CN); UV (CH_3_CN) *λ*_max_ (log ε) 226 (3.99) nm; IR (KBr) *ν*_max_ 3414, 2937, 2873, 1696, 1460, 1372, 1239, 1163, 1043 cm^−1^; ^1^H and ^13^C NMR data (Tables 2 and 3); HRESIMS *m/z* 585.3799 [M − H]^−^ (calcd for C_35_H_53_O_7_^−^, 585.3797).

#### Asprecoside J (10)

White amorphous powder; [*α*]_D_^20^ −13 (*c* 0.1, CH_3_CN); IR (KBr) *ν*_max_ 3418, 2924, 1729, 1443, 1375, 1251, 1168, 1045, 1023, 983 cm^−1^; ^1^H and ^13^C NMR data (Tables 2 and 3); HRESIMS *m/z* 677.3898 [M − H]^−^ (calcd for C_37_H_57_O_11_^−^, 677.3906).

### X-ray crystallographic data of 1 and 2

The X-ray crystallographic data of asprecosides A (**1**) and B (**2**) have been deposited at the Cambridge Crystallographic Data Centre, with the following CCDC deposition numbers: 2413613 (**1**) and 2,413,615 (**2**).

#### Crystallographic data of asprecoside A (1)

C_37_H_56_O_10_⋅4(H_2_O) (*M* = 732.88 g/mol): monoclinic, space group P2_1_ (no. 4), *a* = 13.2729(3) Å, *b* = 7.0751(2) Å, *c* = 20.4063(5) Å, *β* = 96.8560(10)°, *V* = 1902.59(8) Å^3^, *Z* = 2, *T* = 170.00 K, *μ*(CuK*α*) = 0.801 mm^−1^, *Dcalc* = 1.279 g/cm^3^, 22,369 reflections measured (6.708° ≤ 2Θ ≤ 133.79°), 6424 unique (*R*_int_ = 0.0391, *R*_sigma_ = 0.0367) which were used in all calculations. The final *R*_1_ was 0.0387 (I > 2*σ*(I)) and *wR*_2_ was 0.1039 (all data). Flack parameter =  − 0.02(3).

#### Crystallographic data of asprecoside B (2)

C_37_H_56_O_10_ (*M* = 660.81 g/mol): monoclinic, space group P2_1_ (no. 4), *a* = 7.0183(2) Å, *b* = 26.3891(6) Å, *c* = 23.1192(5) Å, *β* = 98.73°, *V* = 4232.22(18) Å^3^, *Z* = 4, *T* = 170.15 K, *μ*(CuK*α*) = 0.605 mm^−1^, *Dcalc* = 1.037 g/cm^3^, 12,902 reflections measured (5.116° ≤ 2Θ ≤ 133.192°), 12,902 unique (*R*_sigma_ = 0.0806) which were used in all calculations. The final *R*_1_ was 0.0631 (I > 2*σ*(I)) and *wR*_2_ was 0.1789 (all data). Flack parameter = 0.09(4).

### Acid hydrolysis of asprecosides A − J (1−10)

Each compound (1 mg) was heated under refluxed in 1.5 mL of 2 M HCl (dioxane/H_2_O, 1:1) at 90 °C for 4 h. Following hydrolysis, 5 mL of water was added, followed by extraction with EtOAc. The aqueous layer was evaporated under vacuum and the residue was dissolved in anhydrous pyridine (400 *μ*L), followed by the addition of 2 mg of L-cysteine methyl ester hydrochloride. After stirring the mixture at 60 °C for 1 h, 50 *μ*L of *o*-tolyl isothiocyanate was added, and the reaction continued at the same temperature for an additional hour. The authentic samples D-xylose and D-glucuronic acid underwent identical treatment and were analyzed by reversed-phase HPLC. D-xylose (2 mL/min, MeCN/H_2_O, 25:75, *t*_R_ = 51.7 min) was identified in **9**, whereas D-glucuronic acid (2 mL/min, MeCN/H_2_O, 50:50, *t*_R_ = 25.2 min) was observed in **1**−**8** and **10**.

### Cell culture

H1975 and HCC827 cells, sourced from the American Type Culture Collection, were cultured in RPMI 1640 medium (Gibco BRL, USA) containing 10% fetal bovine serum (FBS). These cells were grown under conditions of 5% CO_2_ and 37 °C.

### Cell viability assay

Cell viability was evaluated through the Cell Counting Kit-8 (CCK-8) assay. In brief, Briefly, H1975 and HCC827 cells were seeded into 96-well plates (1× 10^3^ cells/well) and incubated for 24 h. Then, the attached cells were treated with different concentrations of compounds. Following a 3-days incubation, CCK-8 reagent (Dojindo) was added, and luminescence was measured according to the manufacturer’s protocol.

## Supplementary Information

Additional file 1.

## Data Availability

All data generated and analyzed during this study are included in this published article and its supplementary information file.
