# Supplementary material for: Asprecosides A–J, ten new pentacyclic triterpenoid glycosides with cytotoxic activity from the roots of Ilex asprella
Source: Nat Prod Bioprospect. 2025 Mar 13;15(1):18. doi: 10.1007/s13659-025-00499-7 (PMC11906957; doi:10.1007/s13659-025-00499-7)

Supplementary Information

Asprecosides A–J, ten new pentacyclic triterpenoid glycosides with cytotoxic activity from the roots of *Ilex asprella*

Yuwei Wu[1,2], Baihui Zhang[1,2], Wenxian Li[2], Lihua Peng[3], Weilin Qiao[3], Wei Li[1,2*] and De-an Guo[1,2*]

[1]School of Pharmaceutical Sciences, Southern Medical University, Guangzhou 510515, People's Republic of China

[2]Zhongshan Institute for Drug Discovery, Shanghai Institute of Materia Medica, Chinese Academy of Sciences, Zhongshan 528400, People's Republic of China

[3]Zhongshan Zhongzhi Pharmaceutical Group Co. Ltd., Zhongshan 528400, People's Republic of China

*Corresponding authors.

E-mail addresses: liwei1@simm.ac.cn (W Li); daguo@simm.ac.cn (D Guo)

Contents

Figure S1. Proposed biosynthetic pathways of compounds **1–17**. 6

S1. 1D NMR calculation of compound 9 6

Figure S2. Two candidate aglycone moieties of **9**. 7

Table S1. Energy (298.15 K) analysis for 3*S**,5*R**,8*R**,9*R**,10*R**,14*S**,17*S**,20*S**-isomer and 3*S**,5*R**,8*R**,9*R**,10*R**,14*S**,17*S**,20*R**-isomer 7

Figure S3. B3LYP/6-31G(d) optimized lowest energy conformers for 3*S**,5*R**,8*R**,9*R**,10*R**,14*S**,17*S**,20*S**-isomer. 8

Figure S4. B3LYP/6-31G(d) optimized lowest energy conformers for 3*S**,5*R**,8*R**,9*R**,10*R**,14*S**,17*S**,20*R**-isomer 8

Table S2. Calculated ^{13}C-NMR chemical shifts for conformers of 3*S**,5*R**,8*R**,9*R**,10*R**,14*S**,17*S**,20*S**-isomer. 9

Table S3. Calculated ^{13}C-NMR chemical shifts for conformers of 3*S**,5*R**,8*R**,9*R**,10*R**,14*S**,17*S**,20*R**-isomer 10

Table S4. Calculated ^{1}H-NMR chemical shifts for conformers of 3*S**,5*R**,8*R**,9*R**,10*R**,14*S**,17*S**,20*S**-isomer. 11

Table S5. Calculated ^{1}H-NMR chemical shifts for conformers of 3*S**,5*R**,8*R**,9*R**,10*R**,14*S**,17*S**,20*R**-isomer 12

Figure S5. DP4+ evaluation of theoretical and experimental NMR data of aglycone moiety of **9**. 14

S2. NMR, HRESIMS, and IR spectra of 1–10 15

Figure S6. ^{1}H NMR spectrum of **1** in pyridine-d_5 (500 MHz) 15

Figure S7. ^{13}C NMR and DEPT spectra of **1** in pyridine-d_5 (125 MHz) 16

Figure S8. HSQC spectrum of **1** in pyridine-d_5 (500 MHz) 17

Figure S9. HMBC spectrum of **1** in pyridine-d_5 (500 MHz) 18

Figure S10. ^{1}H–^{1}H COSY spectrum of **1** in pyridine-d_5 (500 MHz) 19

Figure S11. NOESY spectrum of **1** in pyridine-d_5 (500 MHz) 20

Figure S12. HRESIMS spectrum of **1** 21

Figure S13. IR (KBr disc) spectrum of **1** 22

Figure S14. ^{1}H NMR spectrum of **2** in pyridine-d_5 (500 MHz) 23
Figure S15. ^{13}C NMR and DEPT spectra of **2** in pyridine-d_5 (125 MHz) 24
Figure S16. HSQC spectrum of **2** in pyridine-d_5 (500 MHz) 25
Figure S17. HMBC spectrum of **2** in pyridine-d_5 (500 MHz) 26
Figure S18. ^{1}H–^{1}H COSY spectrum of **2** in pyridine-d_5 (500 MHz) 27
Figure S19. NOESY spectrum of **2** in pyridine-d_5 (500 MHz) 28
Figure S20. HRESIMS spectrum of **2** 29
Figure S21. IR (KBr disc) spectrum of **2** 30
Figure S22. ^{1}H NMR spectrum of **3** in CD_3OD (500 MHz) 31
Figure S23. ^{13}C NMR and DEPT spectra of **3** in CD_3OD (125 MHz) 32
Figure S24. HSQC spectrum of **3** in CD_3OD (500 MHz) 33
Figure S25. HMBC spectrum of **3** in CD_3OD (500 MHz) 34
Figure S26. ^{1}H–^{1}H COSY spectrum of **3** in CD_3OD (500 MHz) 35
Figure S27. NOESY spectrum of **3** in CD_3OD (500 MHz) 36
Figure S28. HRESIMS spectrum of **3** 37
Figure S29. IR (KBr disc) spectrum of **3** 38
Figure S30. ^{1}H NMR spectrum of **4** in pyridine-d_5 (500 MHz) 39
Figure S31. ^{13}C NMR and DEPT spectra of **4** in pyridine-d_5 (125 MHz) 40
Figure S32. HSQC spectrum of **4** in pyridine-d_5 (500 MHz) 41
Figure S33. HMBC spectrum of **4** in pyridine-d_5 (500 MHz) 42
Figure S34. ^{1}H–^{1}H COSY spectrum of **4** in pyridine-d_5 (500 MHz) 43
Figure S35. NOESY spectrum of **4** in pyridine-d_5 (500 MHz) 44
Figure S36. HRESIMS spectrum of **4** 45
Figure S37. IR (KBr disc) spectrum of **4** 46
Figure S38. ^{1}H NMR spectrum of **5** in pyridine-d_5 (500 MHz) 47
Figure S39. ^{13}C NMR and DEPT spectra of **5** in pyridine-d_5 (125 MHz) 48
Figure S40. HSQC spectrum of **5** in pyridine-d_5 (500 MHz) 49
Figure S41. HMBC spectrum of **5** in pyridine-d_5 (500 MHz) 50
Figure S42. ^{1}H–^{1}H COSY spectrum of **5** in pyridine-d_5 (500 MHz) 51
Figure S43. NOESY spectrum of **5** in pyridine-d_5 (500 MHz) 52
Figure S44. HRESIMS spectrum of **5** 53
Figure S45. IR (KBr disc) spectrum of **5** 54

Figure S46. ^{1}H NMR spectrum of **6** in pyridine-d_5 (500 MHz) 55
Figure S47. ^{13}C NMR and DEPT spectra of **6** in pyridine-d_5 (125 MHz) 56
Figure S48. HSQC spectrum of **6** in pyridine-d_5 (500 MHz) 57
Figure S49. HMBC spectrum of **6** in pyridine-d_5 (500 MHz) 58
Figure S50. ^{1}H–^{1}H COSY spectrum of **6** in pyridine-d_5 (500 MHz) 59
Figure S51. NOESY spectrum of **6** in pyridine-d_5 (500 MHz) 60
Figure S52. HRESIMS spectrum of **6** 61
Figure S53. IR (KBr disc) spectrum of **6** 62
Figure S54. ^{1}H NMR spectrum of **7** in pyridine-d_5 (500 MHz) 63
Figure S55. ^{13}C NMR and DEPT spectra of **7** in pyridine-d_5 (125 MHz) 64
Figure S56. HSQC spectrum of **7** in pyridine-d_5 (500 MHz) 65
Figure S57. HMBC spectrum of **7** in pyridine-d_5 (500 MHz) 66
Figure S58. ^{1}H–^{1}H COSY spectrum of **7** in pyridine-d_5 (500 MHz) 67
Figure S59. NOESY spectrum of **7** in pyridine-d_5 (500 MHz) 68
Figure S60. HRESIMS spectrum of **7** 69
Figure S61. IR (KBr disc) spectrum of **7** 70
Figure S62. ^{1}H NMR spectrum of **8** in pyridine-d_5 (500 MHz) 71
Figure S63. ^{13}C NMR and DEPT spectra of **8** in pyridine-d_5 (125 MHz) 72
Figure S64. HSQC spectrum of **8** in pyridine-d_5 (500 MHz) 73
Figure S65. HMBC spectrum of **8** in pyridine-d_5 (500 MHz) 74
Figure S66. ^{1}H–^{1}H COSY spectrum of **8** in pyridine-d_5 (500 MHz) 75
Figure S67. NOESY spectrum of **8** in pyridine-d_5 (500 MHz) 76
Figure S68. HRESIMS spectrum of **8** 77
Figure S69. IR (KBr disc) spectrum of **8** 78
Figure S70. ^{1}H NMR spectrum of **9** in CD_3OD (500 MHz) 79
Figure S71. ^{13}C NMR and DEPT spectra of **9** in CD_3OD (125 MHz) 80
Figure S72. HSQC spectrum of **9** in CD_3OD (500 MHz) 81
Figure S73. HMBC spectrum of **9** in CD_3OD (500 MHz) 82
Figure S74. ^{1}H–^{1}H COSY spectrum of **9** in CD_3OD (500 MHz) 83
Figure S75. NOESY spectrum of **9** in CD_3OD (500 MHz) 84
Figure S76. HRESIMS spectrum of **9** 85
Figure S77. IR (KBr disc) spectrum of **9** 86

Figure S78. ^{1}H NMR spectrum of **10** in pyridine-d_5 (500 MHz) 87
Figure S79. ^{13}C NMR and DEPT spectra of **10** in pyridine-d_5 (125 MHz) 88
Figure S80. HSQC spectrum of **10** in pyridine-d_5 (500 MHz) 89
Figure S81. HMBC spectrum of **10** in pyridine-d_5 (500 MHz) 90
Figure S82. ^{1}H–^{1}H COSY spectrum of **10** in pyridine-d_5 (500 MHz) 91
Figure S83. NOESY spectrum of **10** in pyridine-d_5 (500 MHz) 92
Figure S84. HRESIMS spectrum of **10** 93
Figure S85. IR (KBr disc) spectrum of **10** 94

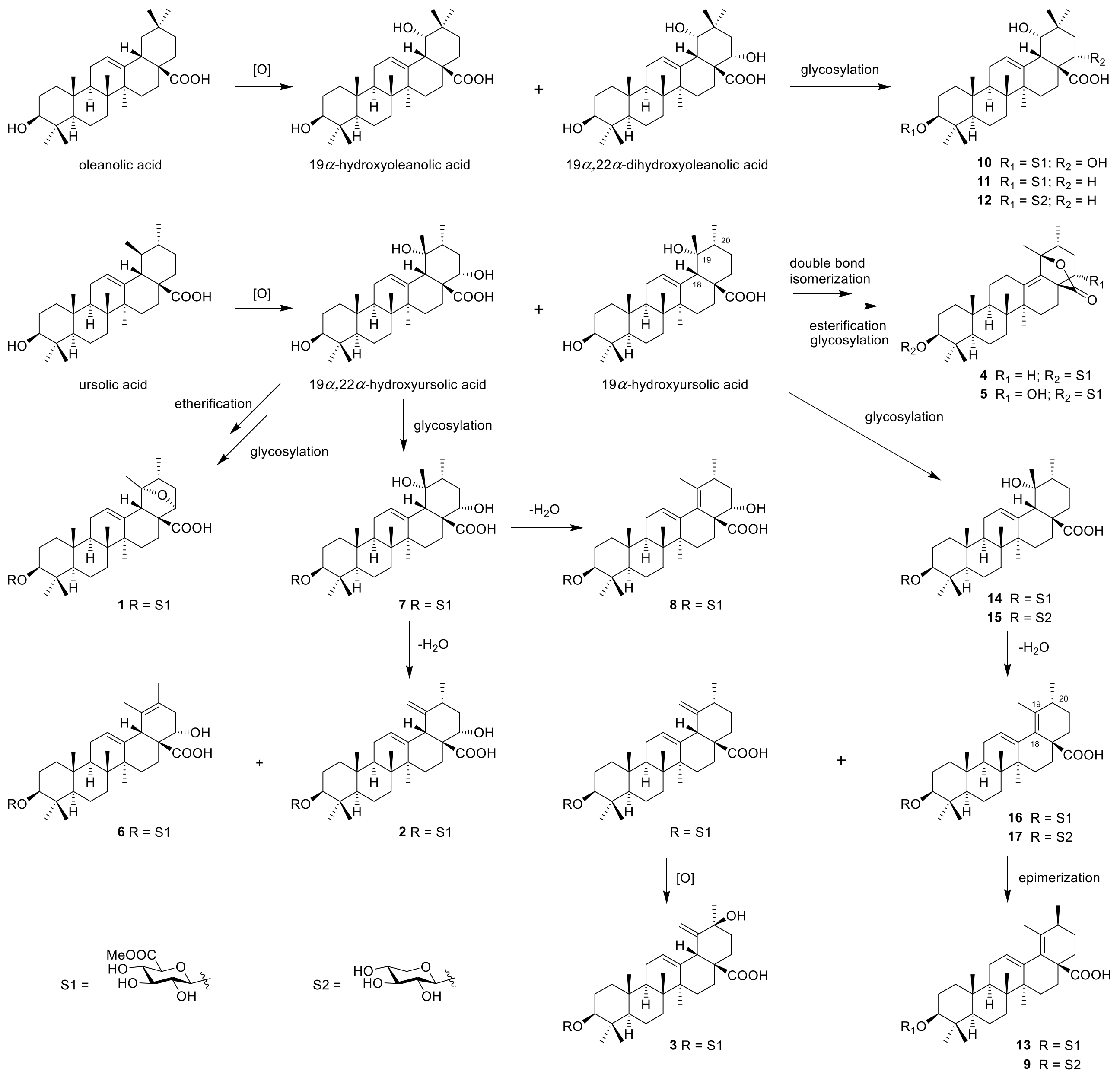

Figure S1. Proposed biosynthetic pathways of compounds **1**–**17**.

S1. 1D NMR calculation of compound 9

Two candidate aglycone moieties of **9** (Figure S2), isomer **1** (3*S**,5*R**,8*R**,9*R**,10*R**,14*S**,17*S**,20*S**-isomer) and isomer **2** (3*S**,5*R**,8*R**,9*R**,10*R**,14*S**,17*S**,20*R**-isomer) were studied by quantum chemical DFT calculations of their theoretical 1D NMR chemical shifts. Conformational analyses were first carried out via Monte Carlo searching using molecular mechanism with MMFF force field in the

Spartan 18 program.[1] The conformers were reoptimized using DFT at the B3LYP/6-31G(d) level using the *Gaussian 09 program*.[2] Five conformers of 3*S**,5*R**,8*R**,9*R**,10*R**,14*S**,17*S**,20*S**-isomer (Figure S3) and three conformers of 3*S**,5*R**,8*R**,9*R**,10*R**,14*S**,17*S**,20*R**-isomer (Figure S4) were refined and considered for next step. Gauge-Independent Atomic Orbital (GIAO) calculations of their ^{1}H and ^{13}C NMR chemical shifts were accomplished by density functional theory (DFT) at the rmpw1pw91/6-31+g(d,p) level in PCM (MeOH). The calculated NMR data of the lowest energy conformers for each compound were averaged according to the Boltzmann distribution theory and their relative Gibbs free energy (ΔG). The ^{1}H and ^{13}C NMR chemical shifts for TMS were calculated by the same protocol and used as the reference. The experimental and calculated data were analyzed by the improved probability DP4+ method.[3]

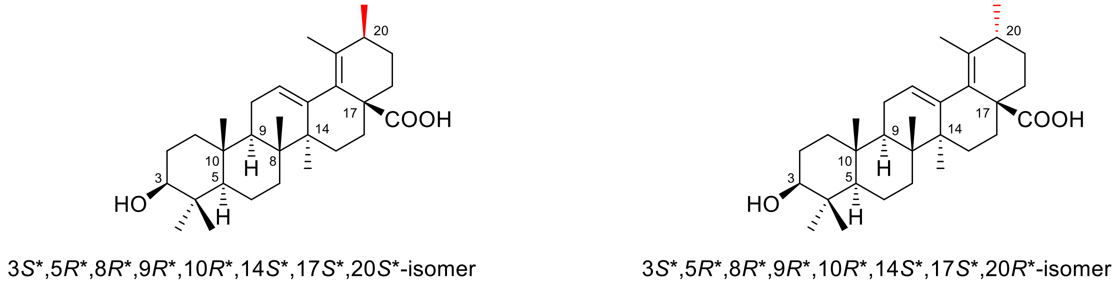

Figure S2. Two candidate aglycone moieties of **9**.

Table S1. Energy (298.15 K) analysis for 3*S**,5*R**,8*R**,9*R**,10*R**,14*S**,17*S**,20*S**-isomer and 3*S**,5*R**,8*R**,9*R**,10*R**,14*S**,17*S**,20*R**-isomer.

Conf.	G (Hartree)	ΔG (Kcal/mol)	Boltzmann Distribution
3*S**,5*R**,8*R**,9*R**,10*R**,14*S**,17*S**,20*S**-isomer**C1**	-1395.8951	0.22527609	0.291864136
3*S**,5*R**,8*R**,9*R**,10*R**,14*S**,17*S**,20*S**-isomer**C2**	-1395.8954	0	0.426966214
3*S**,5*R**,8*R**,9*R**,10*R**,14*S**,17*S**,20*S**-isomer**C3**	-1395.8948	0.39784134	0.218084593
3*S**,5*R**,8*R**,9*R**,10*R**,14*S**,17*S**,20*S**-isomer**C4**	-1395.8927	1.68611937	0.024764456
3*S**,5*R**,8*R**,9*R**,10*R**,14*S**,17*S**,20*S**-isomer**C5**	-1395.8932	1.42758525	0.038320601
3*S**,5*R**,8*R**,9*R**,10*R**,14*S**,17*S**,20*R**-isomer**C1**	-1395.889717	0.25163151	0.305186597
3*S**,5*R**,8*R**,9*R**,10*R**,14*S**,17*S**,20*R**-isomer**C2**	-1395.890118	0	0.466774098
3*S**,5*R**,8*R**,9*R**,10*R**,14*S**,17*S**,20*R**-isomer**C3**	-1395.889442	0.42419676	0.228039305

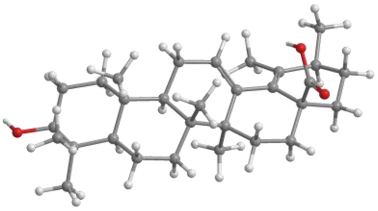

3S*,5R*,8R*,9R*,10R*,14S*,17S*,20S*-isomer**C1**

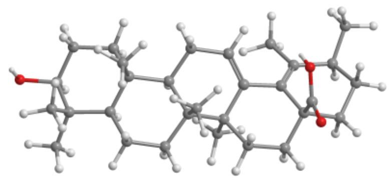

3S*,5R*,8R*,9R*,10R*,14S*,17S*,20S*-isomer**C2**

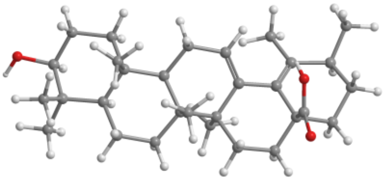

3S*,5R*,8R*,9R*,10R*,14S*,17S*,20S*-isomer**C3**

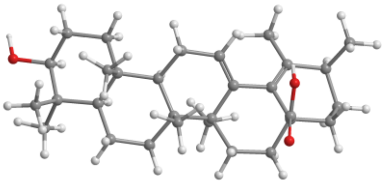

3S*,5R*,8R*,9R*,10R*,14S*,17S*,20S*-isomer**C4**

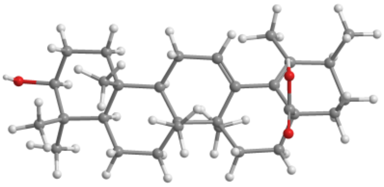

3S*,5R*,8R*,9R*,10R*,14S*,17S*,20S*-isomer**C5**

Figure S3. B3LYP/6-31G(d) optimized lowest energy conformers for 3S*,5R*,8R*,9R*,10R*,14S*,17S*,20S*-isomer.

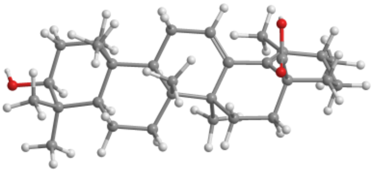

3S*,5R*,8R*,9R*,10R*,14S*,17S*,20R*-isomer**C1**

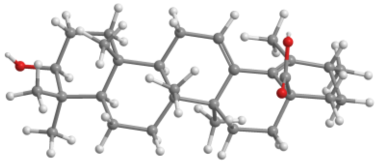

3S*,5R*,8R*,9R*,10R*,14S*,17S*,20R*-isomer**C2**

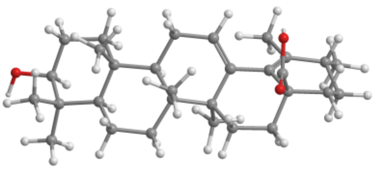

3S*,5R*,8R*,9R*,10R*,14S*,17S*,20R*-isomer**C3**

Figure S4. B3LYP/6-31G(d) optimized lowest energy conformers for 3S*,5R*,8R*,9R*,10R*,14S*,17S*,20R*-isomer.

Table S2. Calculated ^{13}C-NMR chemical shifts for conformers of 3*S**,5*R**,8*R**,9*R**,10*R**,14*S**,17*S**,20*S**-isomer.

NO.	3*S**,5*R**,8*R**,9*R**,10*R**,14*S**,17*S**,20*S**-isomer**C1**	3*S**,5*R**,8*R**,9*R**,10*R**,14*S**,17*S**,20*S**-isomer**C2**	3*S**,5*R**,8*R**,9*R**,10*R**,14*S**,17*S**,20*S**-isomer**C3**	3*S**,5*R**,8*R**,9*R**,10*R**,14*S**,17*S**,20*S**-isomer**C4**	3*S**,5*R**,8*R**,9*R**,10*R**,14*S**,17*S**,20*S**-isomer**C5**
1	39.3592	39.9955	39.6788	39.4068	40.0343
2	30.577	29.785	27.4711	30.5795	29.7907
3	77.5792	77.9499	78.312	77.5932	77.9545
4	42.324	42.7098	42.745	42.3294	42.7214
5	56.2382	56.3868	55.2353	56.2051	56.3535
6	21.6811	22.0337	21.9783	21.6897	22.0432
7	36.7484	36.6954	36.7797	37.1243	37.0703
8	43.6128	43.5878	43.5858	43.7526	43.721
9	50.1878	50.1426	50.26	50.2791	50.2145
10	41.2855	41.0727	41.2822	41.3125	41.1048
11	26.8869	26.8704	26.8283	26.4107	26.3946
12	129.9416	129.8935	129.9338	128.1837	128.1396
13	139.3733	139.3578	139.3387	140.1299	140.113
14	49.7161	49.7136	49.7025	50.433	50.4284
15	31.0224	31.0211	31.0255	31.4451	31.4264
16	36.2228	36.2061	36.2223	34.4159	34.4092
17	56.3067	56.2806	56.3	56.2917	56.28
18	133.7433	133.7497	133.7272	132.5273	132.5188
19	147.4934	147.5122	147.511	145.8696	145.862
20	40.3476	40.3331	40.3496	39.3243	39.3129
21	30.0685	30.0599	30.0669	28.0243	28.0147
22	37.8381	37.8185	37.8323	31.9467	31.9308
23	29.2383	28.9761	28.3583	29.2584	28.9988
24	18.3378	17.3217	18.4583	18.3654	17.3464
25	18.5016	18.325	18.6833	18.4988	18.3247
26	20.4961	20.4798	20.4557	20.7743	20.7564
27	24.1724	24.1596	24.1652	23.9614	23.9529
28	173.3408	173.3169	173.339	172.3414	172.3393
29	23.3567	23.3426	23.3585	23.2097	23.1967
30	21.7293	21.7268	21.7305	21.2767	21.2857

Table S3. Calculated ^{13}C-NMR chemical shifts for conformers of 3*S**,5*R**,8*R**,9*R**,10*R**,14*S**,17*S**,20*R**-isomer.

NO.	3*S**,5*R**,8*R**,9*R**,10*R**,14*S**,17*S**,20*R**-isomer**C1**	3*S**,5*R**,8*R**,9*R**,10*R**,14*S**,17*S**,20*R**-isomer**C2**	3*S**,5*R**,8*R**,9*R**,10*R**,14*S**,17*S**,20*R**-isomer**C3**
1	39.4346	40.0662	39.7528
2	30.5897	29.7999	27.4771
3	77.5932	77.9498	78.315
4	42.3318	42.7235	42.7545
5	56.1968	56.3476	55.196
6	21.7146	22.0691	22.0045
7	37.3387	37.2904	37.37
8	43.9126	43.8836	43.8816
9	50.4105	50.3531	50.4763
10	41.3042	41.0929	41.2998
11	26.3411	26.3259	26.2802
12	127.5146	127.4726	127.511
13	140.7233	140.7077	140.6899
14	50.7145	50.7116	50.6993
15	31.5384	31.5249	31.5367
16	34.4917	34.4818	34.5025
17	56.8979	56.8834	56.891
18	132.826	132.815	132.8064
19	145.2001	145.1953	145.2171
20	37.9263	37.9244	37.929
21	30.658	30.6492	30.6592
22	35.9974	35.9782	36.0023
23	29.263	29.0021	28.3818
24	18.3622	17.3525	18.4912
25	18.5521	18.3729	18.7269
26	20.9372	20.9218	20.8972
27	23.8912	23.8838	23.8898
28	172.2976	172.2961	172.3022
29	19.4915	19.4836	19.4968
30	21.567	21.5617	21.5655

Table S4. Calculated ^{1}H-NMR chemical shifts for conformers of 3*S**,5*R**,8*R**,9*R**,10*R**,14*S**,17*S**,20*S**-isomer.

NO.	3*S**,5*R**,8*R**,9*R**,10*R**,14*S**,17*S**,20*S**-isomer**C1**	3*S**,5*R**,8*R**,9*R**,10*R**,14*S**,17*S**,20*S**-isomer**C2**	3*S**,5*R**,8*R**,9*R**,10*R**,14*S**,17*S**,20*S**-isomer**C3**	3*S**,5*R**,8*R**,9*R**,10*R**,14*S**,17*S**,20*S**-isomer**C4**	3*S**,5*R**,8*R**,9*R**,10*R**,14*S**,17*S**,20*S**-isomer**C5**
1	1.607717	1.640617	1.649117	1.607217	1.639617
	0.993017	1.023217	1.004517	0.979417	1.008517
2	1.259217	1.496317	1.410917	1.250517	1.487917
	1.634217	1.429317	1.564317	1.630617	1.423117
3	3.376617	3.093217	3.318417	3.369817	3.086117
5	0.853617	0.870017	0.830417	0.841017	0.857317
6	1.453417	1.451917	1.442017	1.437817	1.436017
	1.388517	1.375717	1.394017	1.377017	1.364117
7	1.404417	1.400117	1.403317	1.393917	1.389617
	1.499717	1.504917	1.495017	1.518317	1.522917
9	1.390017	1.391117	1.397117	1.348517	1.350717
11	2.057717	2.051317	2.058217	2.008817	2.002517
	1.888017	1.885417	1.901117	1.868717	1.866317
12	5.768217	5.764817	5.771217	5.597117	5.594917
15	1.590417	1.588917	1.591217	1.959817	1.962417
	1.157017	1.156017	1.154017	1.140817	1.139417
16	2.128617	2.128217	2.128317	1.792017	1.790117
	1.332017	1.331917	1.331117	1.768417	1.769417
20	2.251017	2.252517	2.251917	2.206817	2.207917
21	1.106117	1.108217	1.107417	1.395417	1.395917
	1.496417	1.497517	1.497217	1.717217	1.718617
22	1.440517	1.442617	1.440917	1.380417	1.380317
	1.678117	1.676717	1.677917	1.909217	1.909517
23	0.980217	0.929217	1.141817	0.981417	0.930117
	1.551517	1.483117	0.861617	1.545717	1.478217
	0.306217	0.279217	0.272817	0.301117	0.273917
24	0.939517	0.839517	0.956117	0.936617	0.836217
	0.825517	0.518417	0.749117	0.818417	0.512017
	0.573717	0.730217	0.557317	0.571317	0.728917
25	1.084317	1.067917	1.085817	1.083617	1.067417
	0.865017	0.859417	0.883717	0.845817	0.841217

	0.922317	0.863317	0.922817	0.925417	0.865417
27	1.282717	1.270617	1.276817	1.287517	1.275917
	0.462217	0.461417	0.460517	0.475317	0.475717
	0.909317	0.908117	0.907617	0.885417	0.883717
29	1.043617	1.047217	1.042617	0.970217	0.973617
	1.169517	1.171317	1.169717	1.267717	1.269817
	0.659917	0.661017	0.657217	0.663317	0.664917
30	1.381617	1.384117	1.384017	1.191317	1.194217
	2.104017	2.106517	2.107117	2.351517	2.353017
	1.903717	1.904117	1.907517	1.899317	1.900517

Table S5. Calculated ^{1}H-NMR chemical shifts for conformers of 3*S**,5*R**,8*R**,9*R**,10*R**,14*S**,17*S**,20*R**-isomer.

NO.	3*S**,5*R**,8*R**,9*R**,10*R**,14*S**,17*S**,20*R**-isomer**C1**	3*S**,5*R**,8*R**,9*R**,10*R**,14*S**,17*S**,20*R**-isomer**C2**	3*S**,5*R**,8*R**,9*R**,10*R**,14*S**,17*S**,20*R**-isomer**C3**
1	1.613517	1.646017	1.655317
	0.977417	1.006817	0.988617
2	1.250217	1.488117	1.403117
	1.630117	1.423117	1.560017
3	3.368617	3.084917	3.310817
5	0.841717	0.858417	0.818517
6	1.437417	1.435917	1.426117
	1.375017	1.361617	1.380017
7	1.395117	1.390917	1.394117
	1.537117	1.542217	1.532117
9	1.315917	1.317917	1.323417
11	2.013417	2.006817	2.014017
	1.863417	1.860717	1.876417
12	5.621517	5.619717	5.624817
15	2.008017	2.009617	2.008717
	1.135717	1.134617	1.133017

16	1.768917	1.767517	1.768617
	1.793017	1.793717	1.792817
20	2.359517	2.360217	2.361017
21	1.487417	1.488017	1.487717
	1.381017	1.382017	1.381417
22	1.523517	1.523417	1.523817
	1.680217	1.680417	1.681417
23	0.980317	0.928517	1.142417
	1.547017	1.478717	0.855317
	0.301317	0.274317	0.268217
24	0.935217	0.835217	0.952417
	0.819817	0.515317	0.741417
	0.569417	0.725617	0.554117
25	1.084517	1.067717	1.085617
	0.845117	0.839417	0.863717
	0.928917	0.869017	0.929617
27	1.286517	1.273217	1.281217
	0.470217	0.470517	0.468217
	0.888617	0.887417	0.887117
29	0.938217	0.942017	0.937617
	1.327617	1.329517	1.326917
	0.658317	0.659717	0.655917
30	1.523817	1.526317	1.526317
	2.376817	2.378917	2.379517
	1.397017	1.398217	1.400917

Functional	Solvent?	Basis Set	Type of Data
mPW1PW91	PCM	6-31+G(d,p)	Unscaled Shifts

		DP4+	100.00%	0.00%	–	–	–
**Nuclei**	**sp2?**	**xperimenta**	**Isomer 1**	**Isomer 2**	**Isomer 3**	**Isomer 4**	**Isomer 5**
C		40.3	39.7276275	39.8019766			
C		27.2	29.5314242	29.5112467			
C		90.6	77.9120172	77.9242504			
C		40.2	42.5958995	42.6110276			
C		57.2	56.0865288	56.0389678			
C		19.2	21.9105519	21.94618			
C		35.8	36.7542412	37.3232924			
C		40.2	43.6038459	43.8919943			
C		49.2	50.187531	50.3987122			
C		37.8	41.187666	41.2045673			
C		24.2	26.8364172	26.3201174			
C	x	127.2	129.806775	127.494175			
C	x	139.9	139.406219	140.708402			
C		45.7	49.757116	50.7096802			
C		29.8	31.0484705	31.5317109			
C		35.9	36.1013155	34.4895418			
C		49.5	56.2927004	56.8895583			
C	x	134.5	133.665484	132.816396			
C	x	137	147.402537	145.201736			
C		35.7	40.2768534	37.9260288			
C		27.5	29.9351528	30.654166			
C		32.4	37.456198	35.9895553			
C		28.6	28.925755	28.9402704			
C		17.1	17.8929313	17.9203153			
C		16.7	18.4589755	18.5083154			
C		18.6	20.4971942	20.9208901			
C		22.3	24.151728	23.8874266			
C		180.5	173.267075	172.297949			
C		19.8	23.3413007	19.4890211			
C		19.1	21.7002869	21.564184			
H		1.03	1.00867619	0.99369387			
H		1.73	1.6320026	1.63821887			
H		1.84	1.52330855	1.51750887			
H		1.68	1.40208226	1.39612943			
H		3.15	3.23162139	3.22301218			
H		0.81	0.8553891	0.84422128			
H		1.55	1.44923695	1.43413966			
H		1.39	1.38303115	1.36990209			
H		1.58	1.50226155	1.53835702			
H		1.5	1.40151365	1.39292818			
H		1.44	1.38950101	1.31856051			
H		1.96	2.05176685	2.01047278			
H		1.96	1.88845395	1.86512089			
H	x	5.38	5.75654108	5.621429			
H		1.17	1.15485982	1.13458751			
H		1.9	1.61335394	2.00892313			
H		1.38	1.35934633	1.76819477			
H		2.17	2.10707322	1.7932978			
H		2.19	2.24910719	2.36018547			
H		1.36	1.12556647	1.38157466			
H		1.83	1.51104363	1.48776514			
H		1.63	1.43770529	1.5235384			
H		1.81	1.69206575	1.68058367			
H		1.06	0.99179372	0.99310294			
H		1.06	1.36890308	1.35740121			
H		1.06	0.2860405	0.28116567			
H		0.86	0.89640991	0.89246153			
H		0.86	0.66554434	0.65980567			
H		0.86	0.64284821	0.63883778			
H		1.01	1.07697659	1.07692571			
H		1.01	0.86531633	0.84669759			
H		1.01	0.89513103	0.90111653			
H		0.94	1.27612197	1.27909996			
H		0.94	0.46234609	0.46990062			
H		0.94	0.90686069	0.88771448			
H		1	1.04043551	0.93985358			
H		1	1.17660425	1.32834391			
H		1	0.6600733	0.65842286			
H		1.74	1.37131353	1.5255537			
H		1.74	2.12143118	2.3784126			
H		1.74	1.90448459	1.39846615			
H		1.1	0.93619899	0.83067359			
H		1.1	1.40304057	1.44272172			
H		1.1	0.61911834	0.71859154			

Functional	Solvent?	Basis Set	Type of Data
mPW1PW91	PCM	6-31+G(d,p)	Unscaled Shifts

	Isomer 1	Isomer 2	Isomer 3	Isomer 4	Isomer 5	Isomer 6
sDP4+ (H data)	99.87%	0.13%	–	–	–	–
sDP4+ (C data)	49.52%	50.48%	–	–	–	–
sDP4+ (all data)	99.87%	0.13%	–	–	–	–
uDP4+ (H data)	99.96%	0.04%	–	–	–	–
uDP4+ (C data)	76.01%	23.99%	–	–	–	–
uDP4+ (all data)	99.99%	0.01%	–	–	–	–
DP4+ (H data)	100.00%	0.00%	–	–	–	–
DP4+ (C data)	75.66%	24.34%	–	–	–	–
DP4+ (all data)	100.00%	0.00%	–	–	–	–

Figure S5. DP4+ evaluation of theoretical and experimental NMR data of aglycone moiety of **9**.

S2. NMR, HRESIMS, and IR spectra of 1–10

Figure S6. ^{1}H NMR spectrum of **1** in pyridine-d_5 (500 MHz)

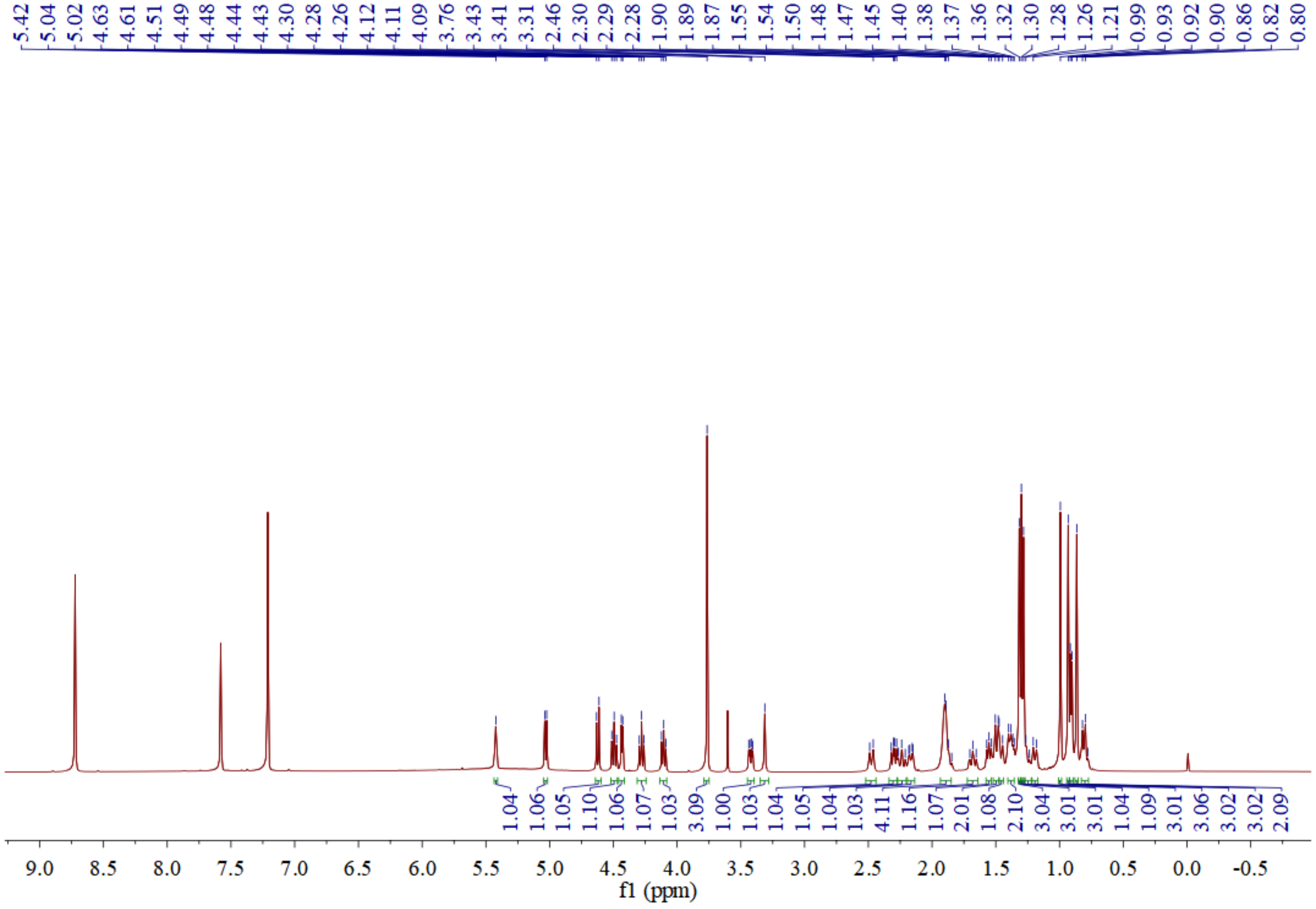

Figure S7. ^{13}C NMR and DEPT spectra of **1** in pyridine-d_5 (125 MHz)

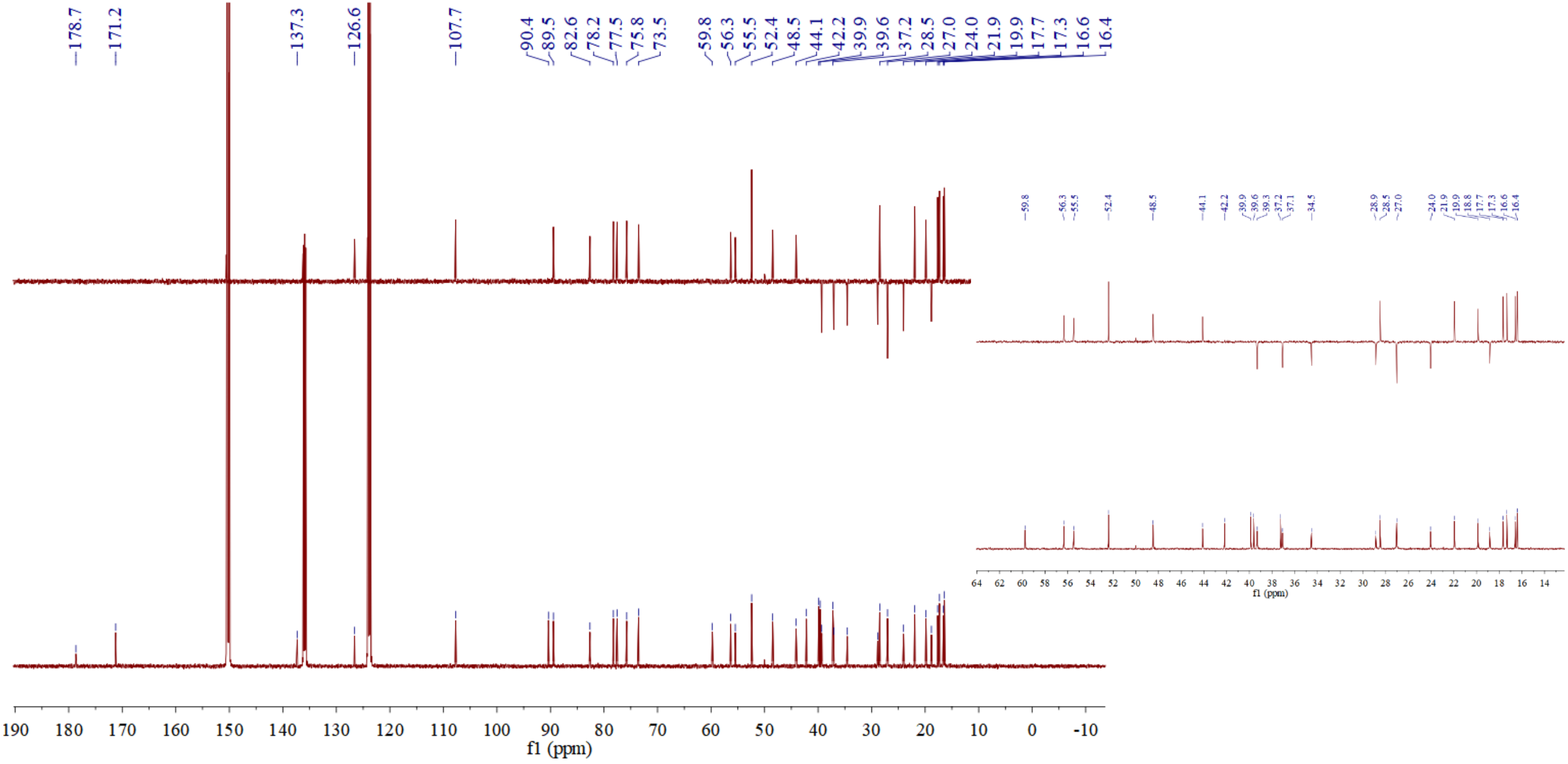

Figure S8. HSQC spectrum of **1** in pyridine-d_5 (500 MHz)

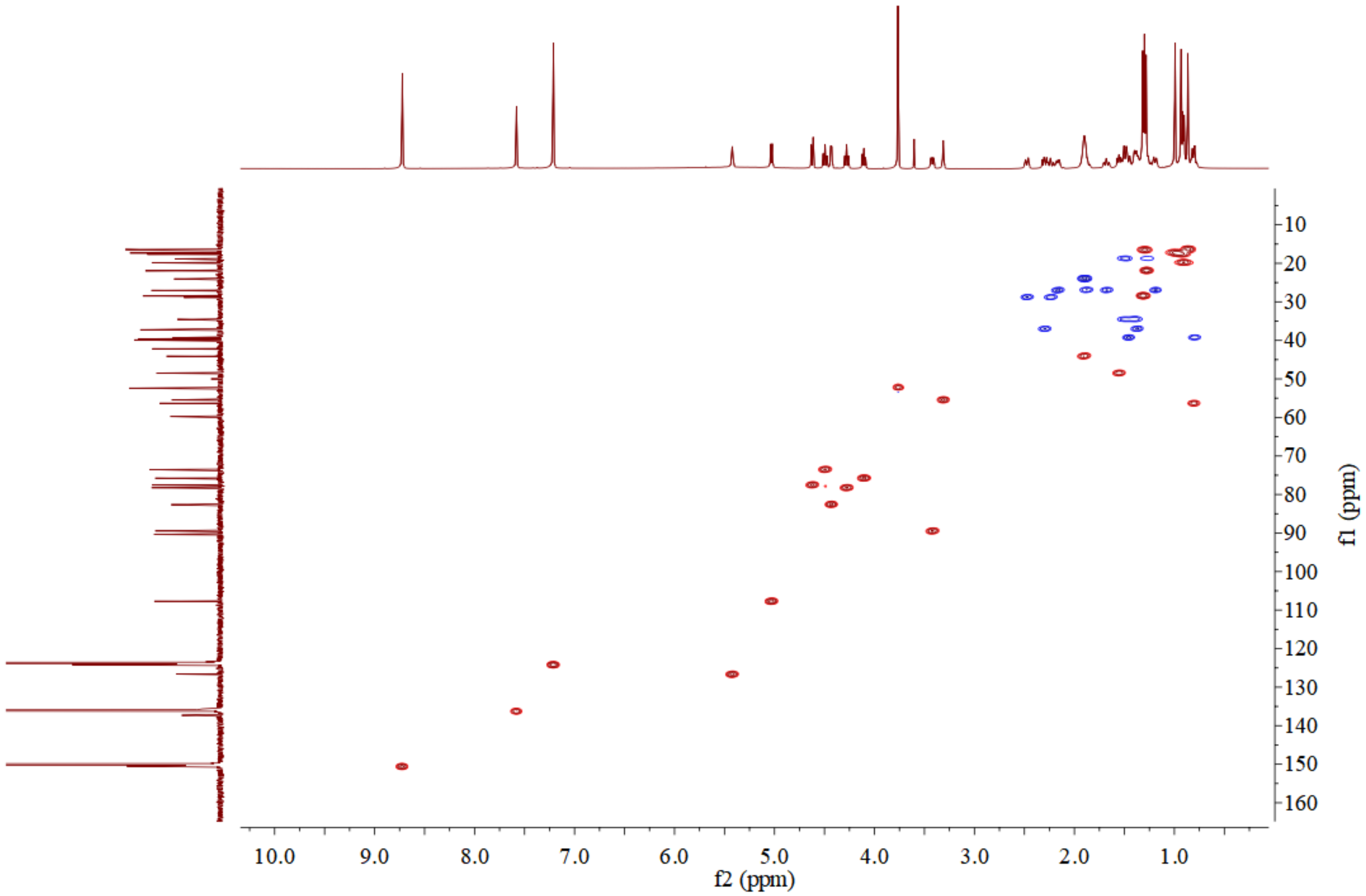

Figure S9. HMBC spectrum of **1** in pyridine-d_5 (500 MHz)

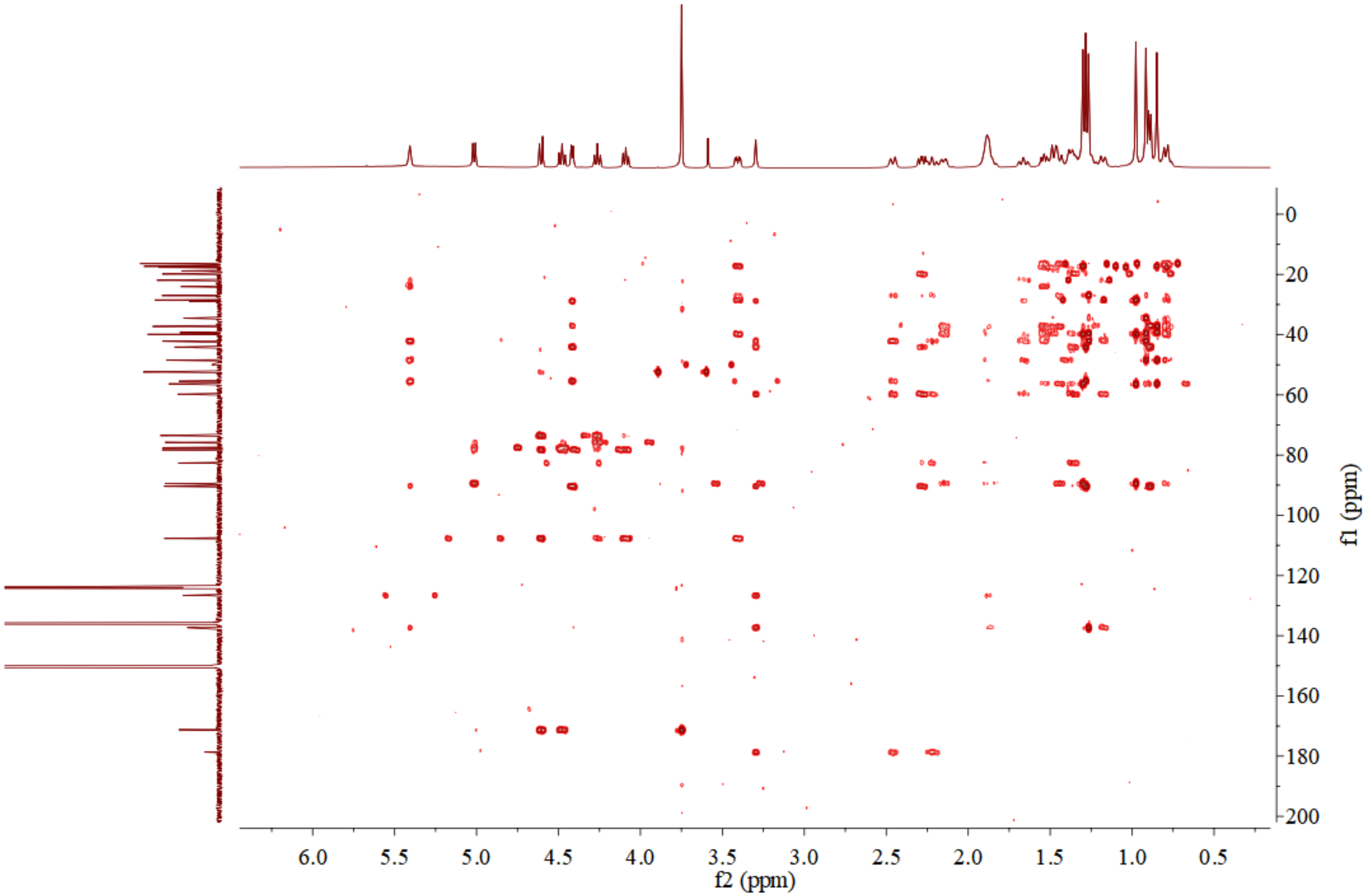

Figure S10. ^1H–^1H COSY spectrum of **1** in pyridine-d_5 (500 MHz)

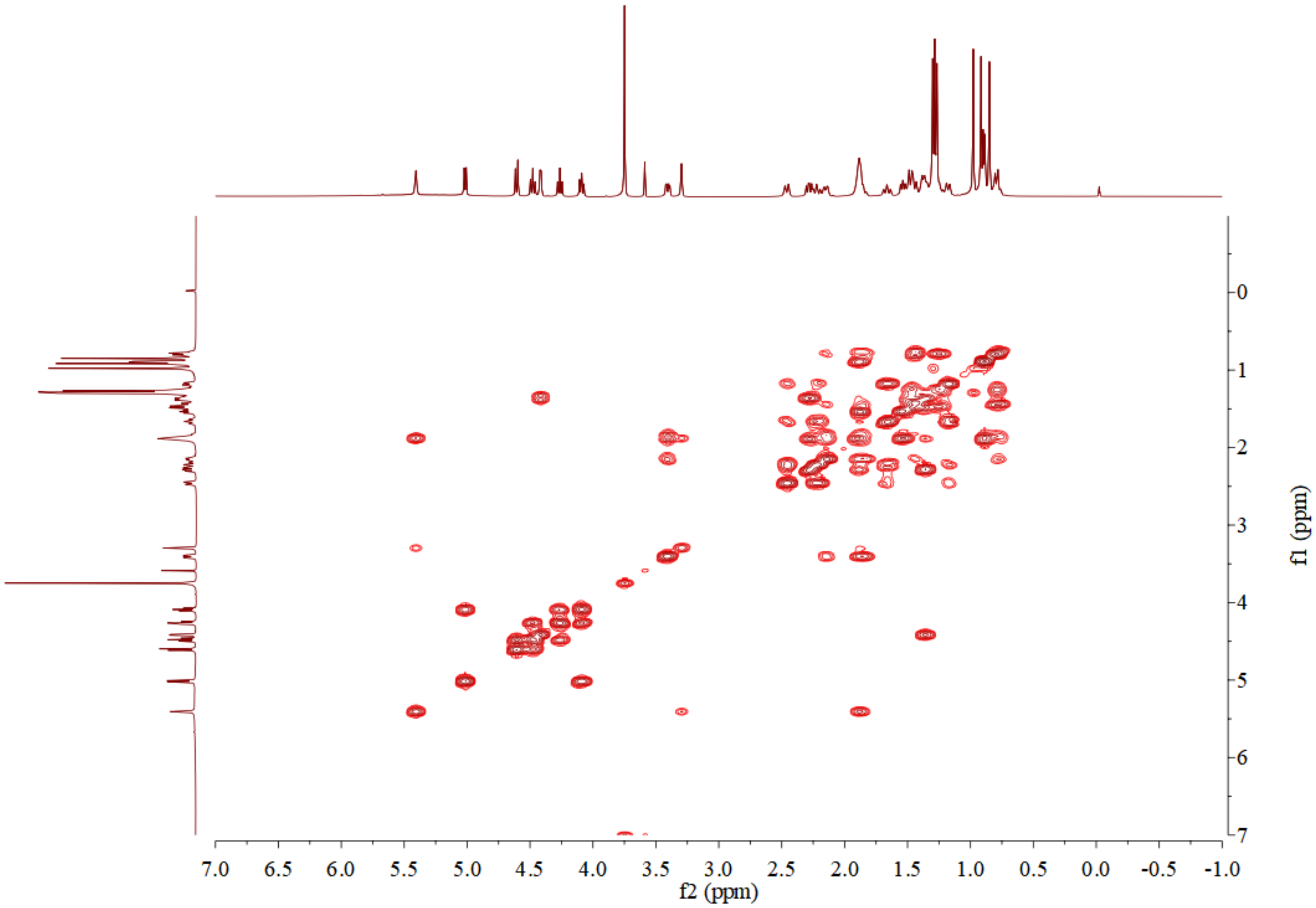

Figure S11. NOESY spectrum of **1** in pyridine-d_5 (500 MHz)

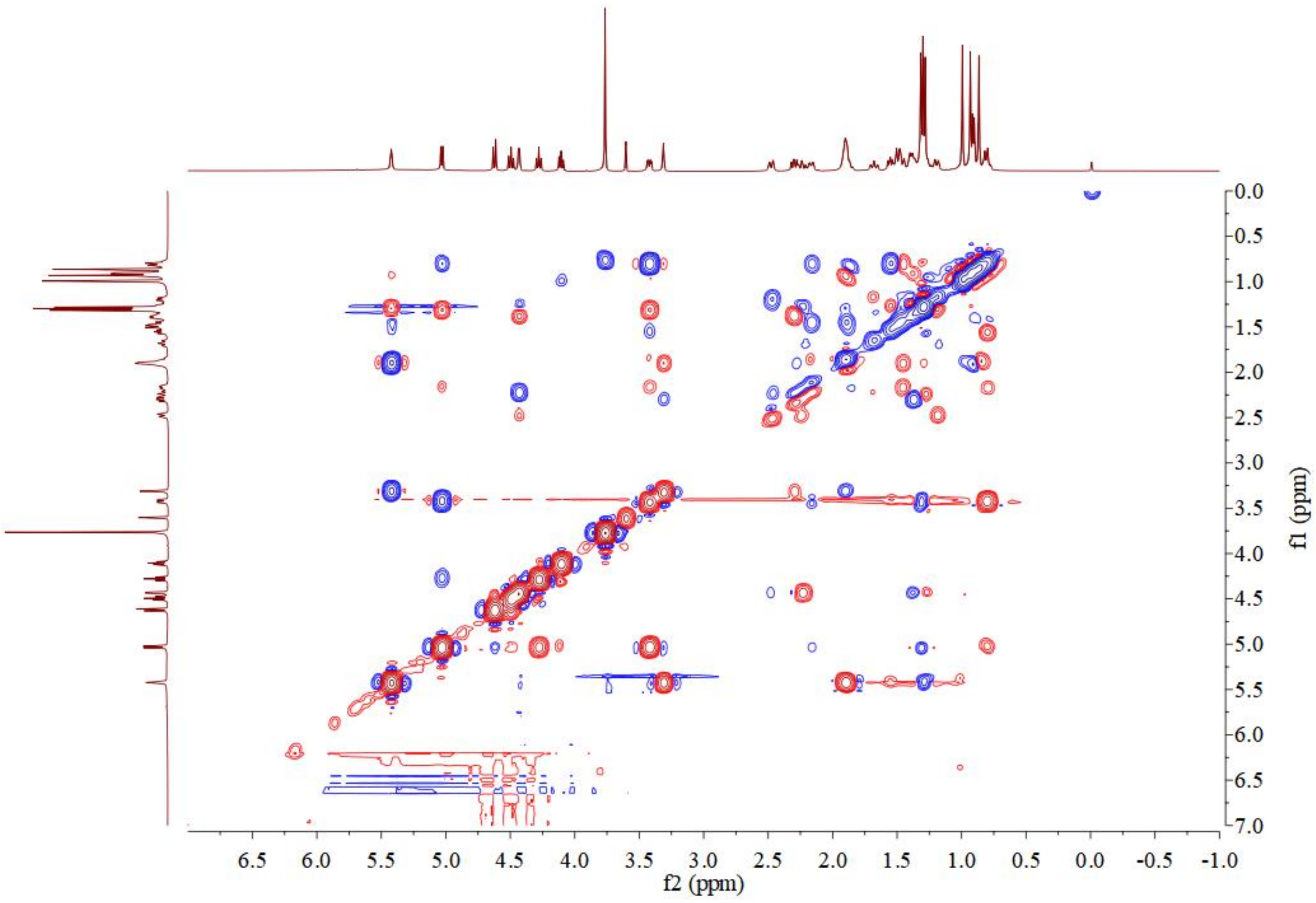

Figure S12. HRESIMS spectrum of **1**

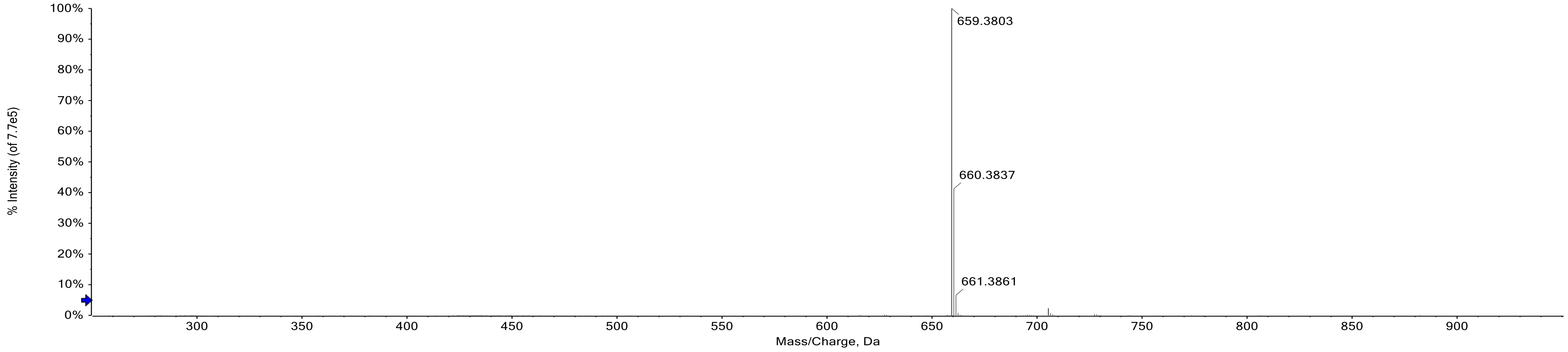

Figure S13. IR (KBr disc) spectrum of **1**

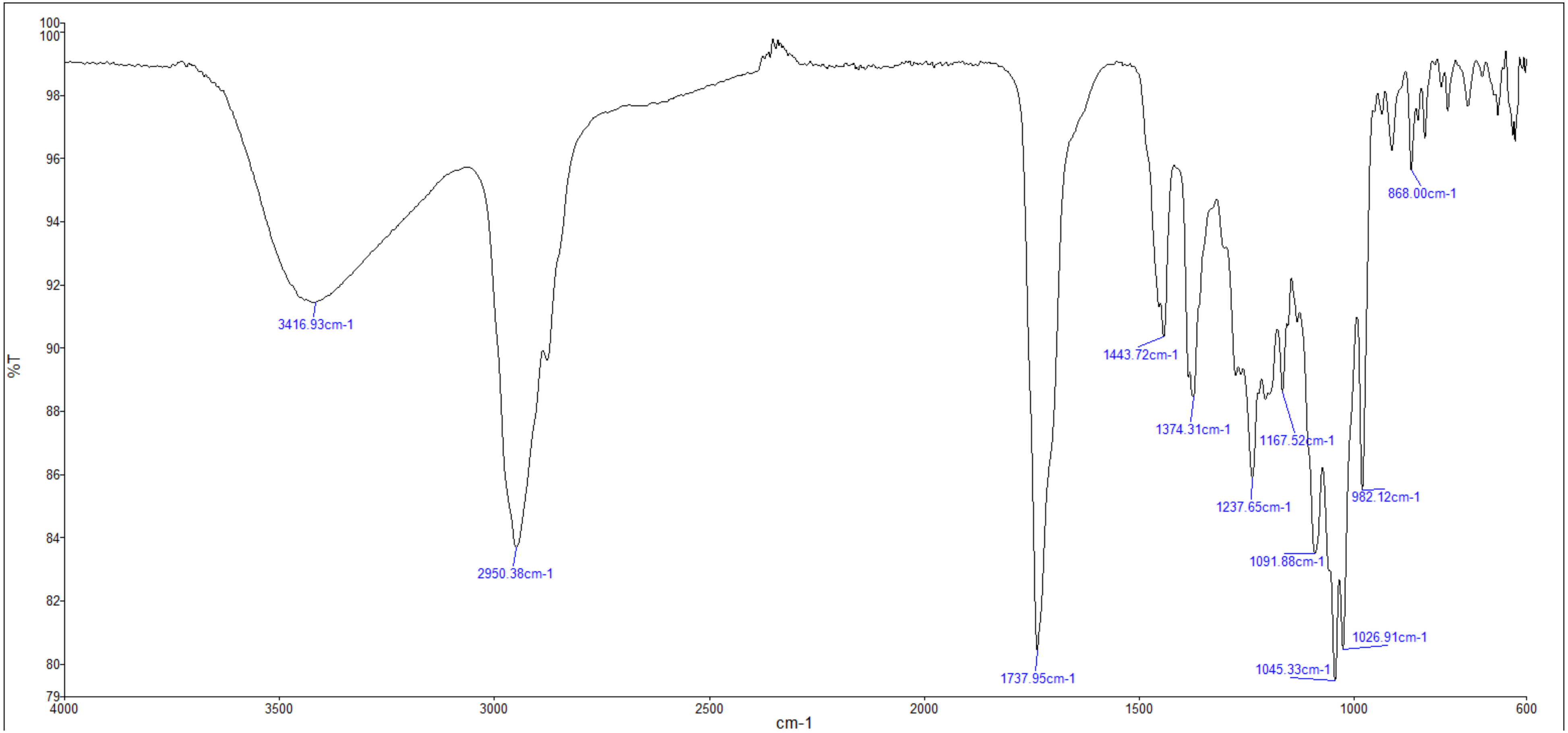

Figure S14. ^{1}H NMR spectrum of **2** in pyridine-d_5 (500 MHz)

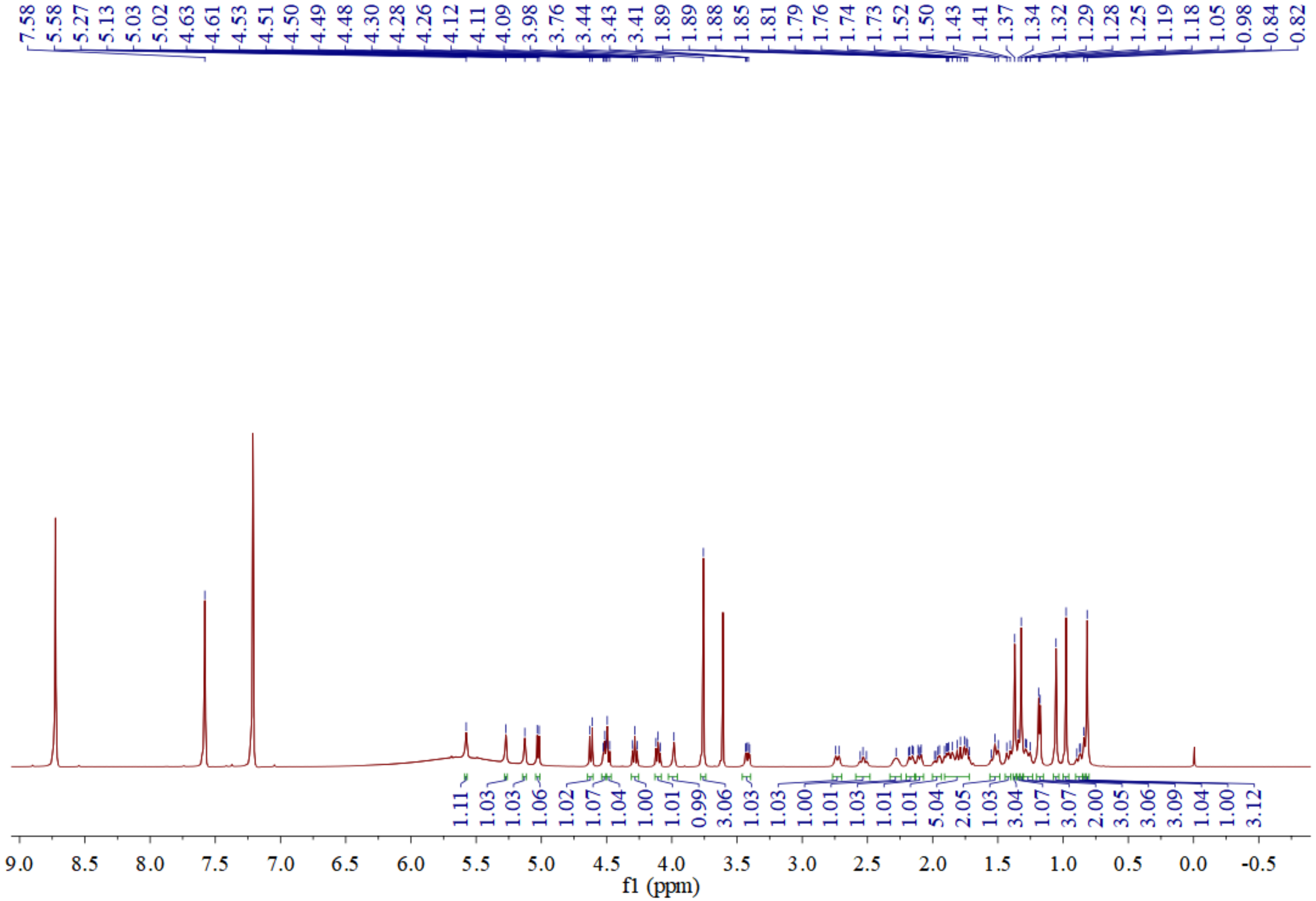

Figure S15. ^{13}C NMR and DEPT spectra of **2** in pyridine-d_5 (125 MHz)

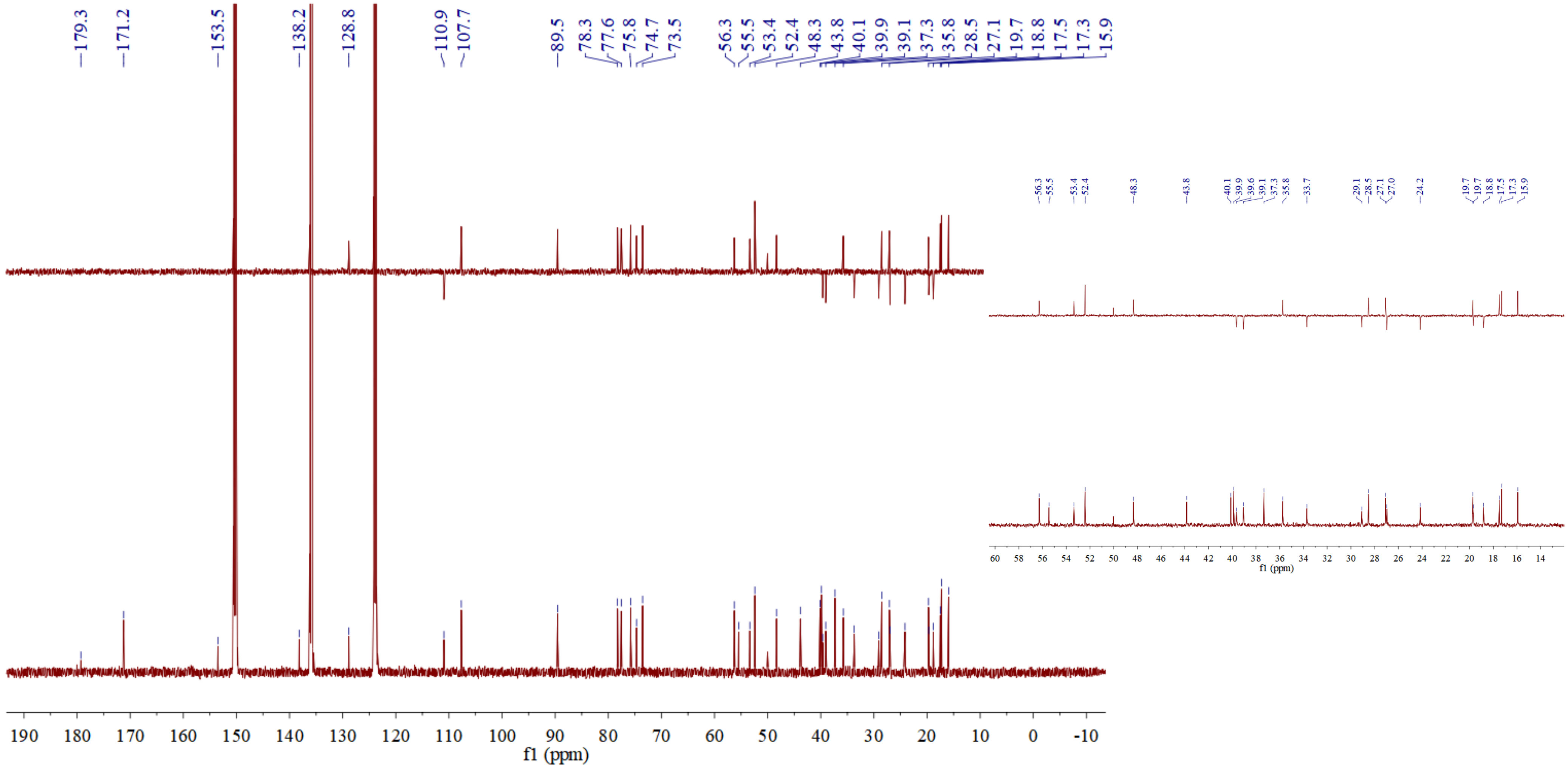

Figure S16. HSQC spectrum of **2** in pyridine-d_5 (500 MHz)

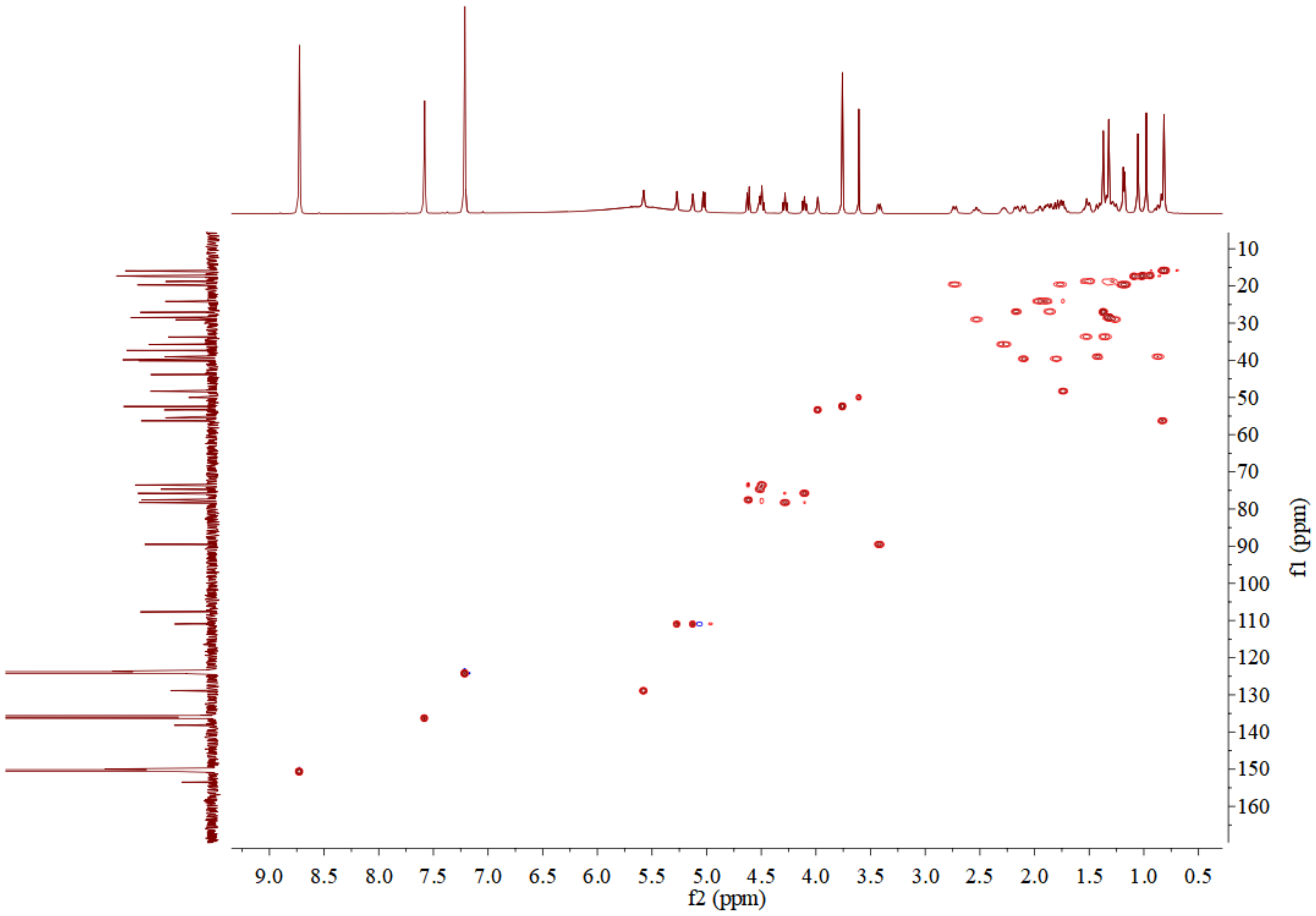

Figure S17. HMBC spectrum of **2** in pyridine-d_5 (500 MHz)

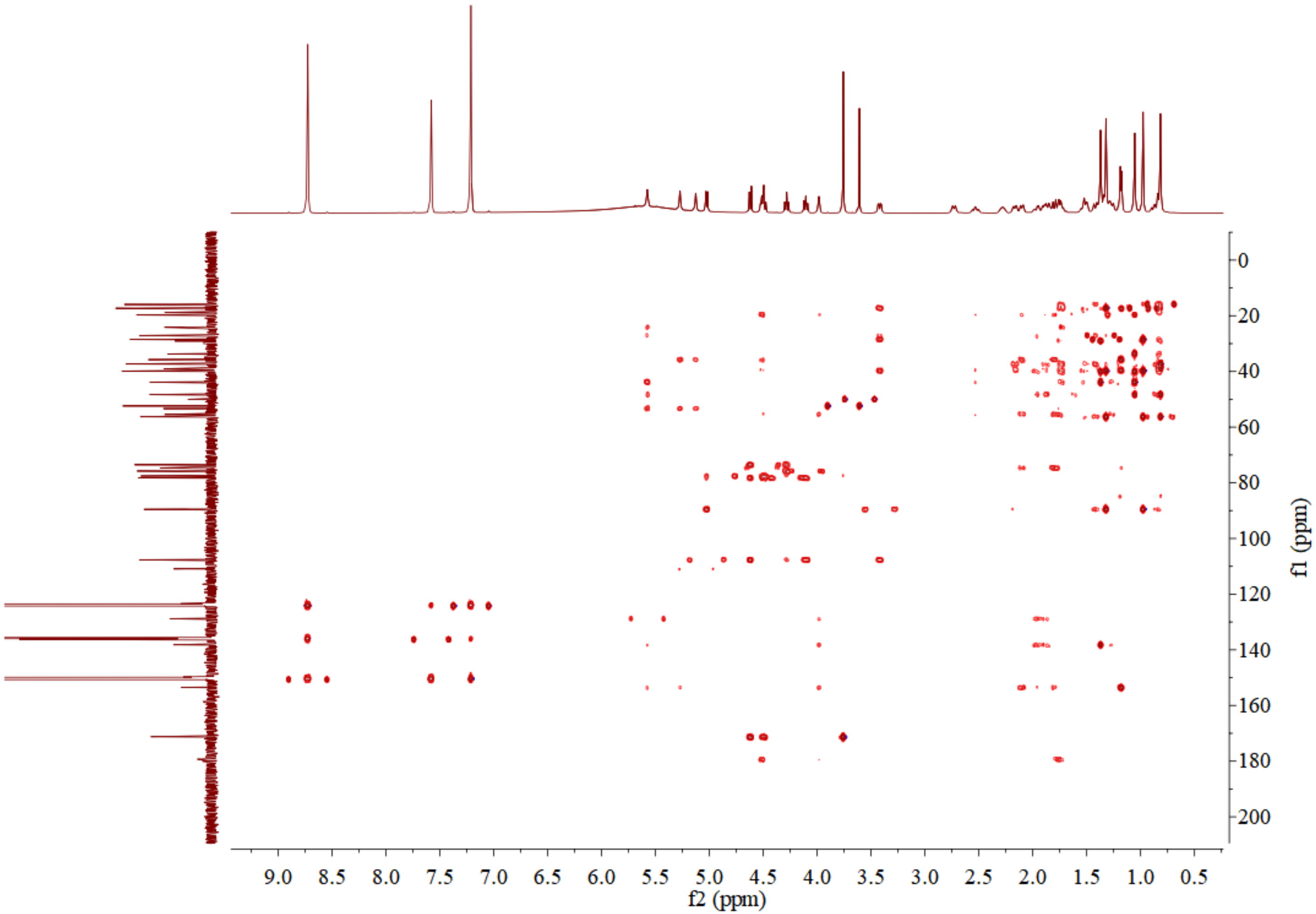

Figure S18. ^1H–^1H COSY spectrum of **2** in pyridine-d_5 (500 MHz)

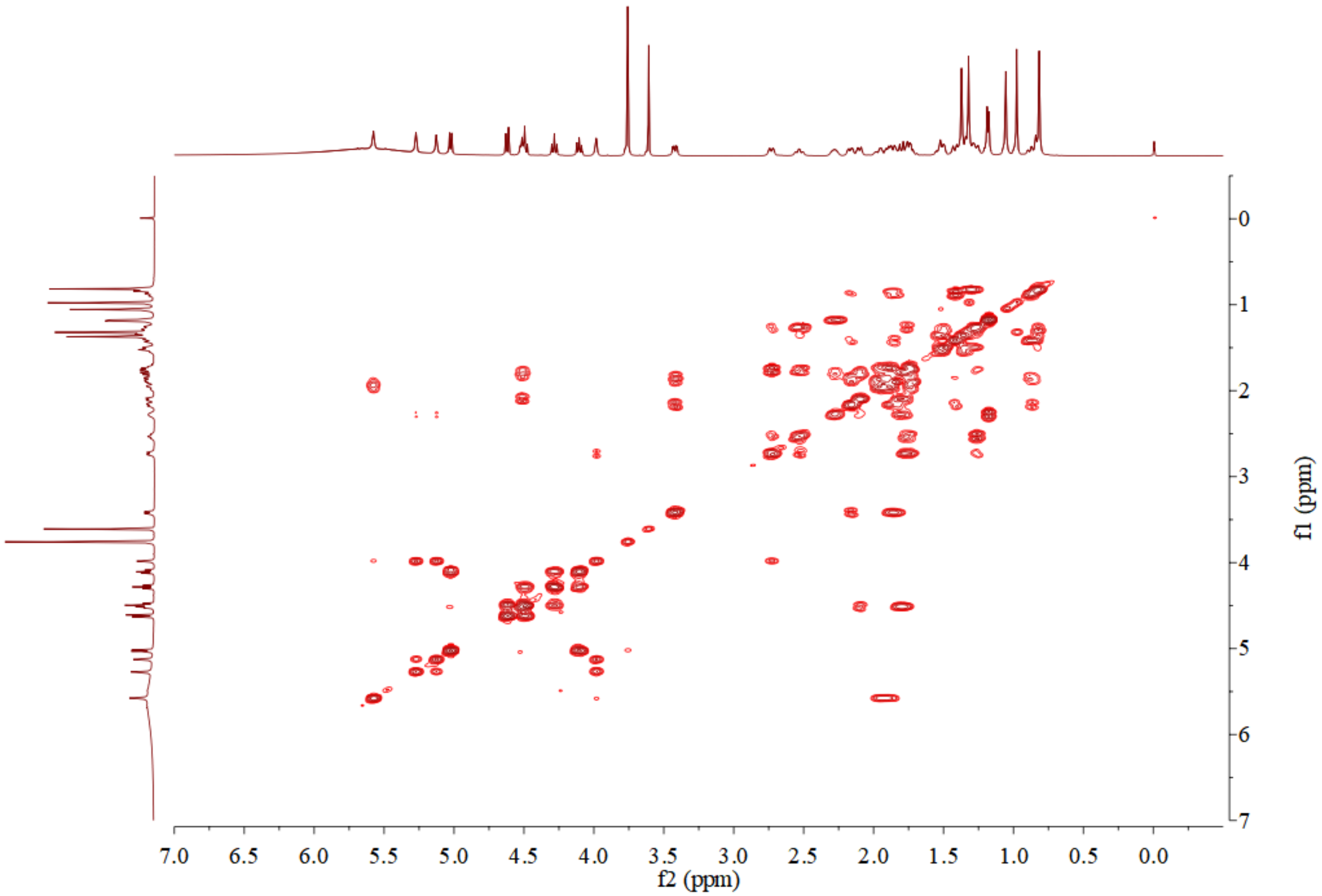

Figure S19. NOESY spectrum of **2** in pyridine-d_5 (500 MHz)

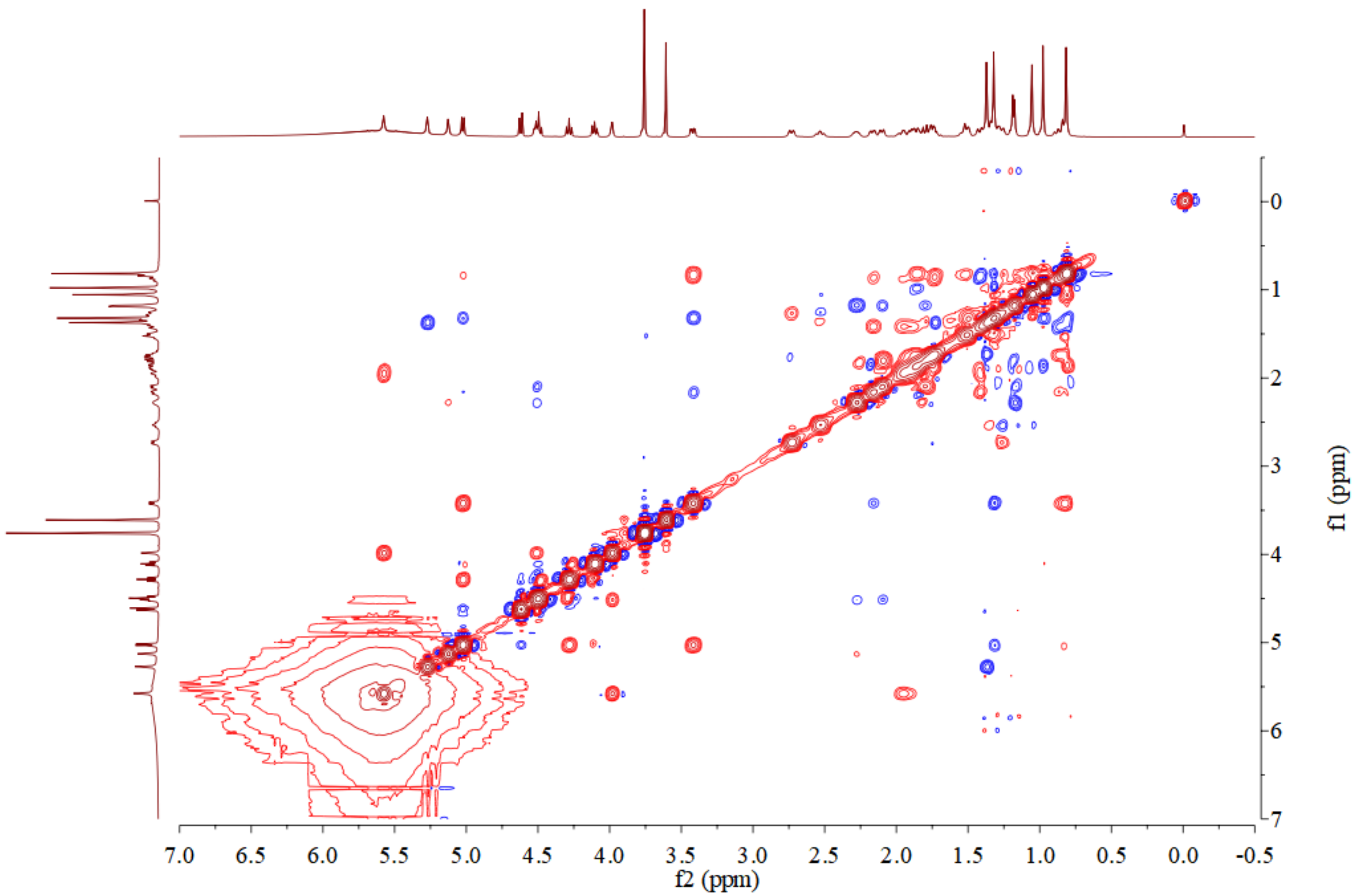

Figure S20. HRESIMS spectrum of **2**

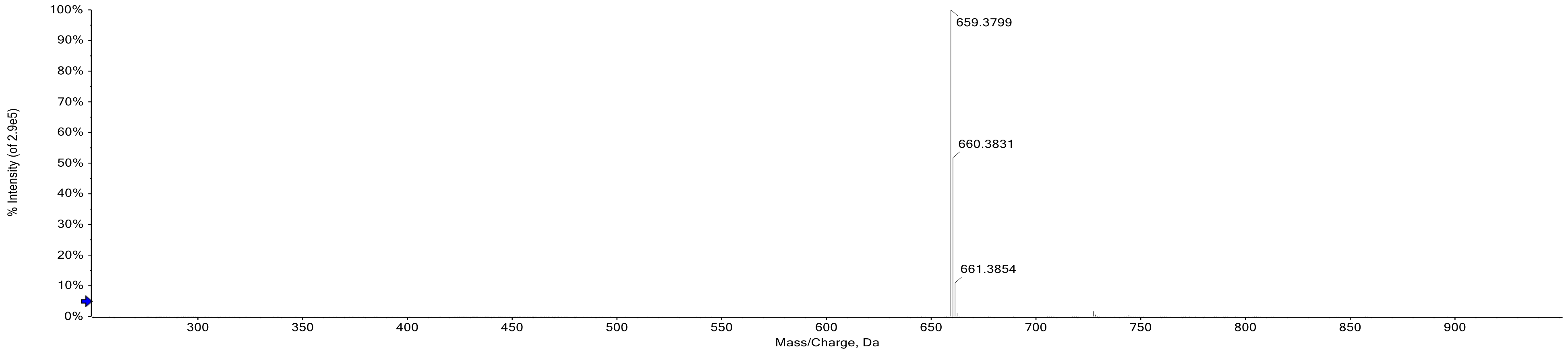

Figure S21. IR (KBr disc) spectrum of **2**

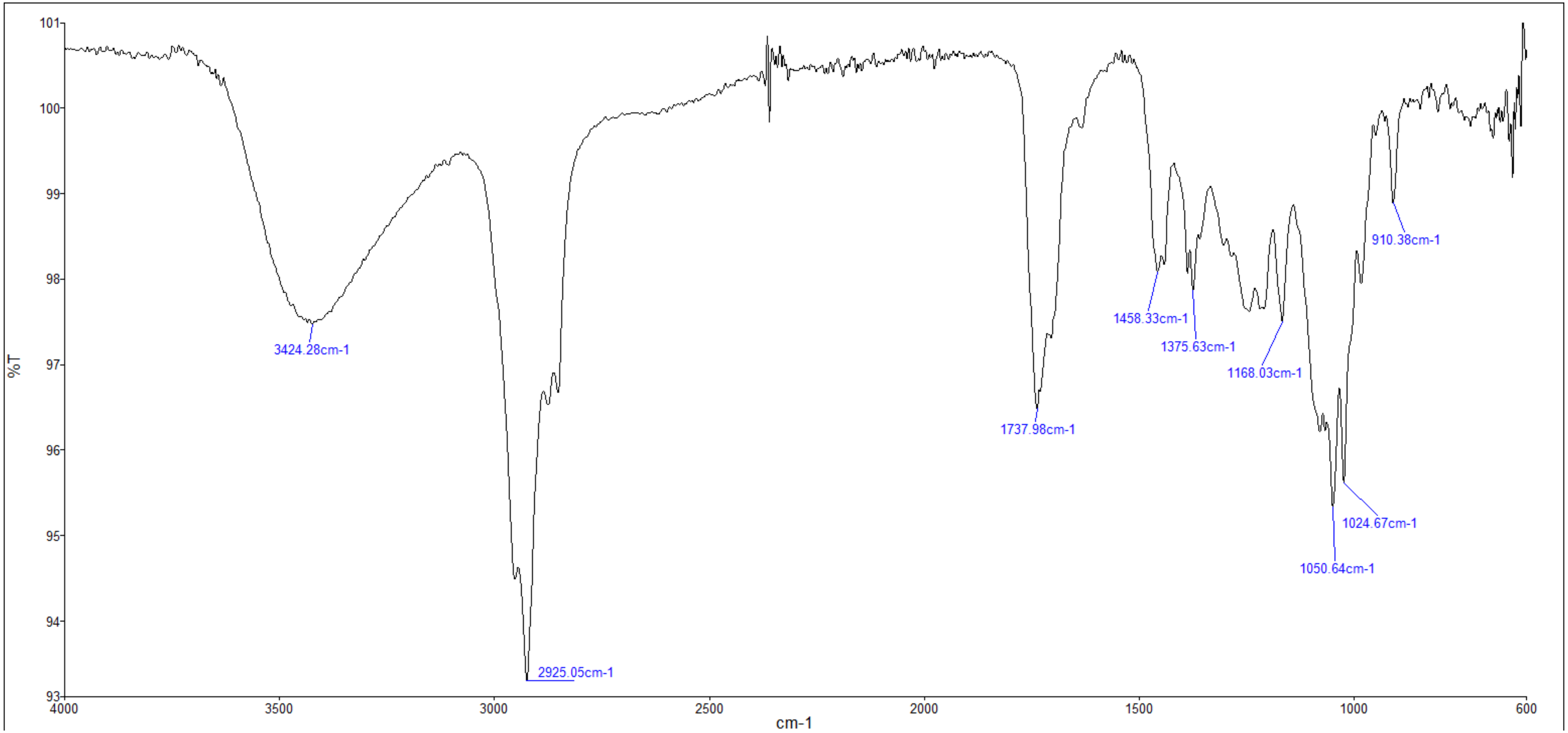

Figure S22. ^{1}H NMR spectrum of **3** in CD_3OD (500 MHz)

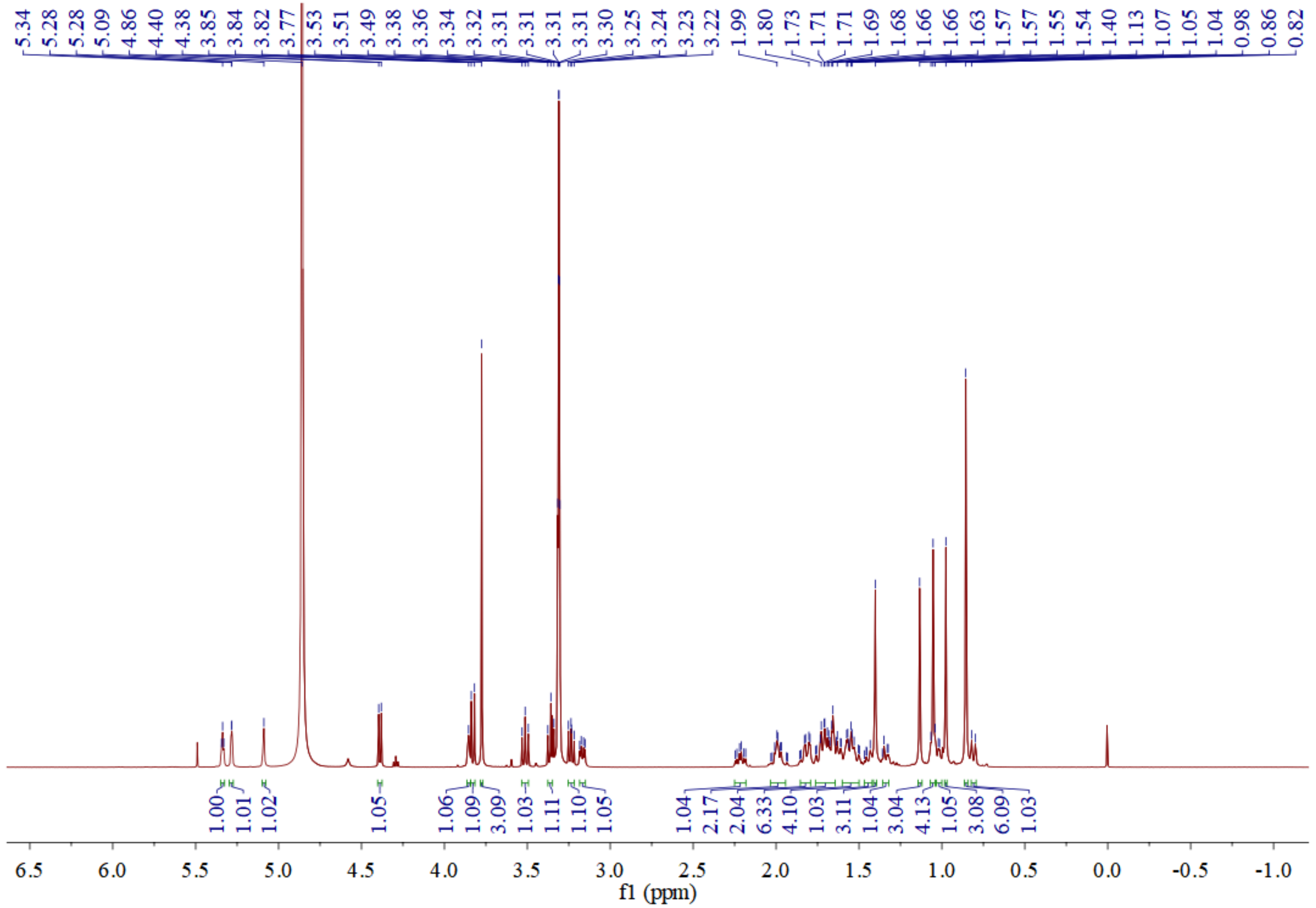

Figure S23. ^{13}C NMR and DEPT spectra of **3** in CD_3OD (125 MHz)

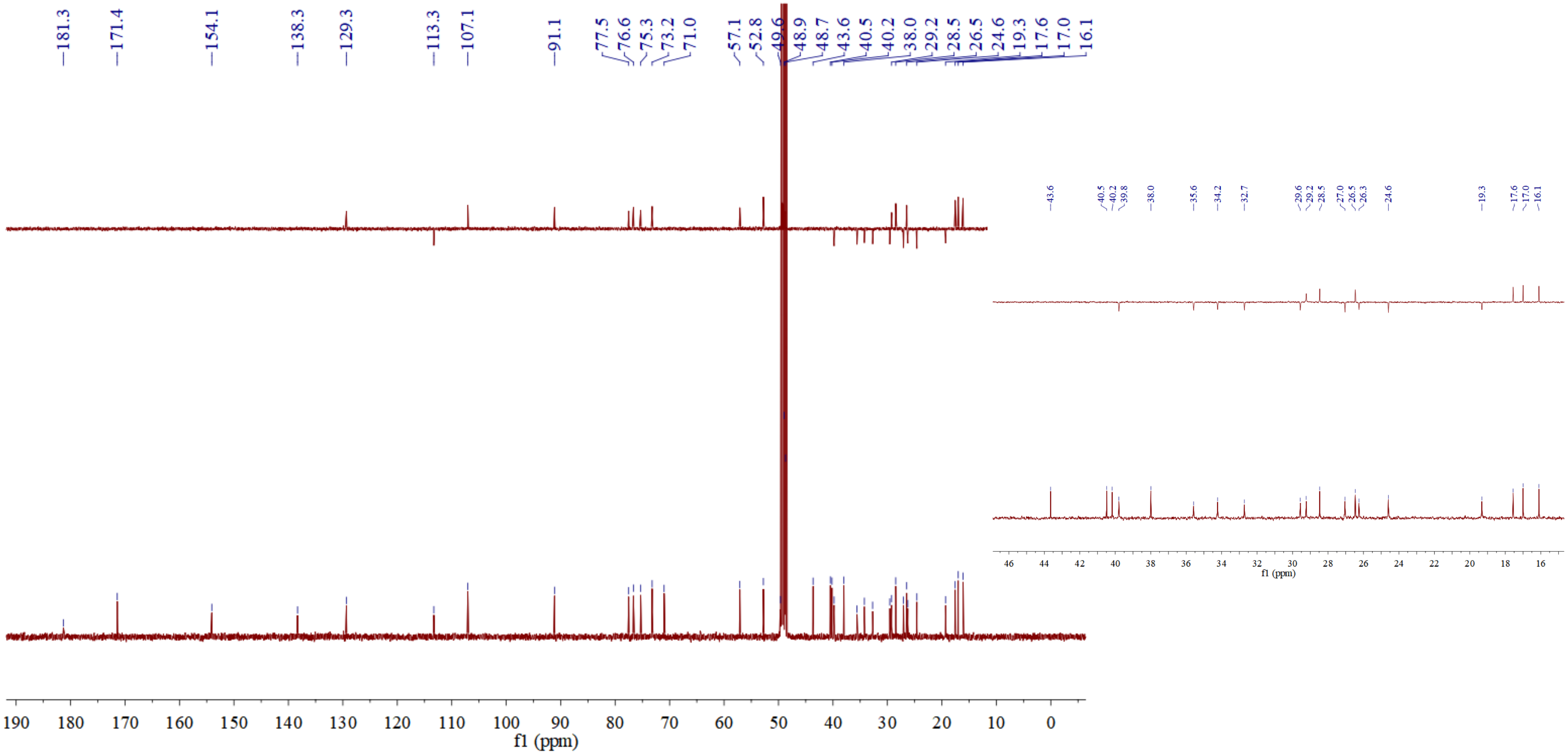

Figure S24. HSQC spectrum of **3** in CD_3OD (500 MHz)

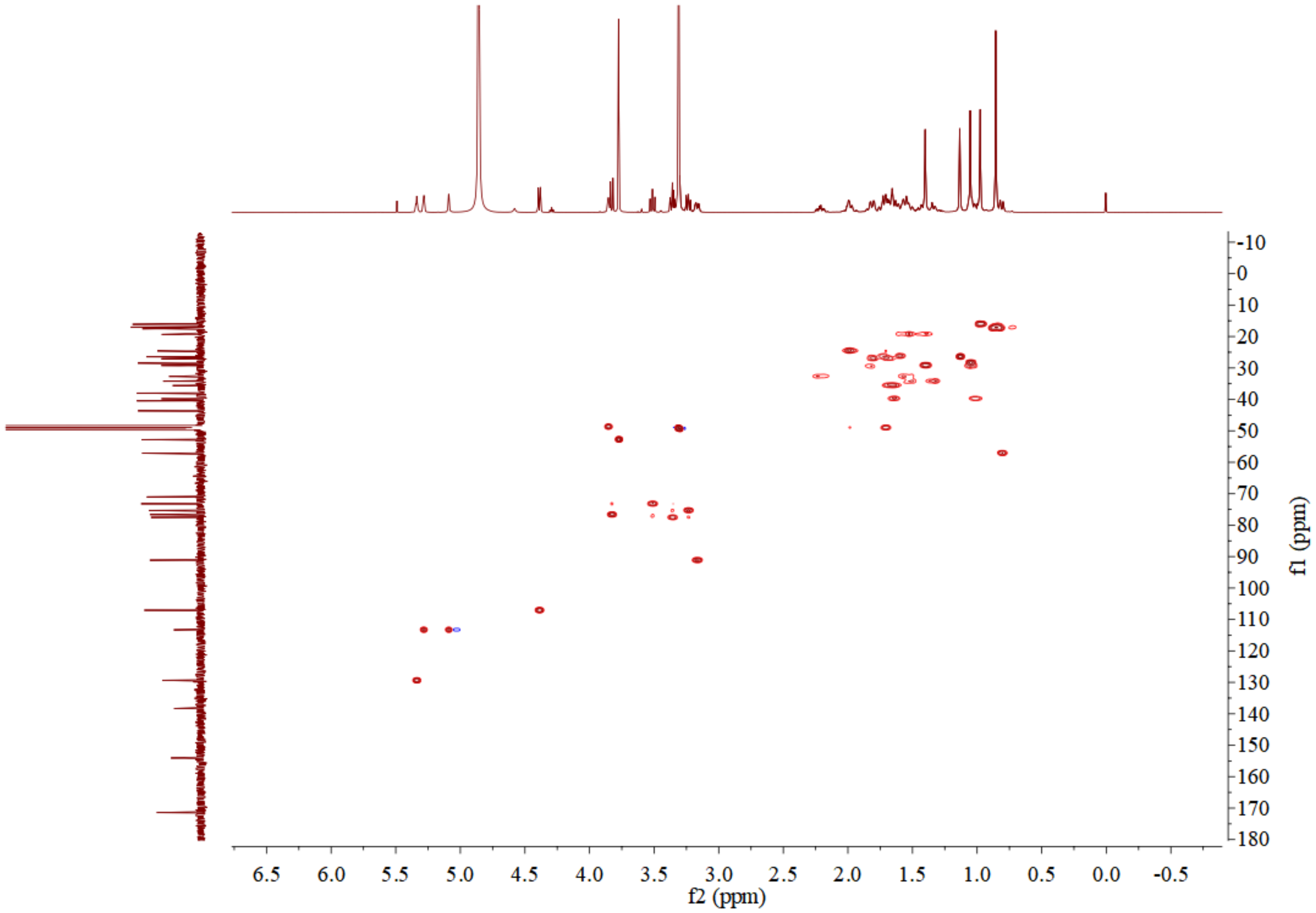

Figure S25. HMBC spectrum of **3** in CD_3OD (500 MHz)

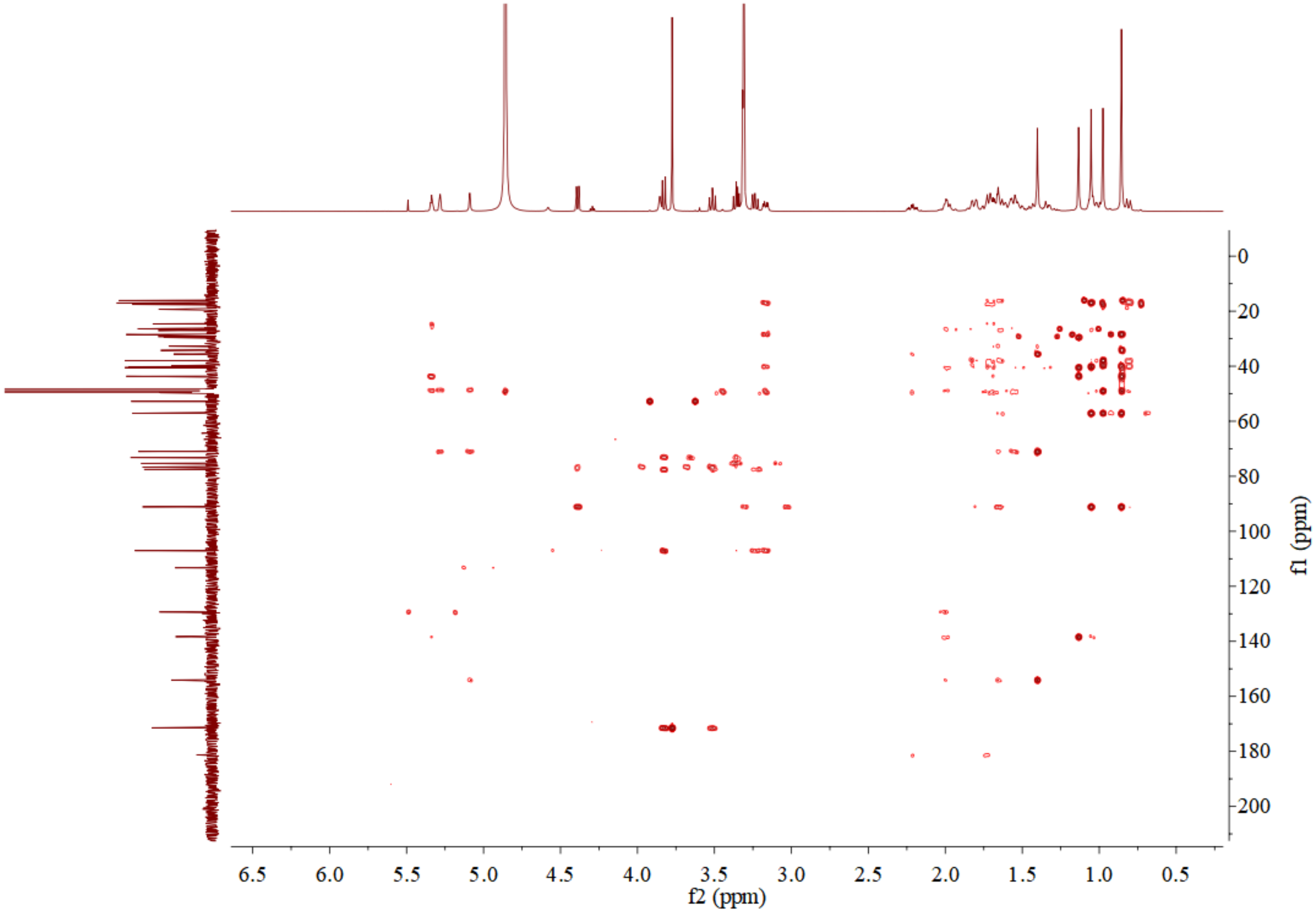

Figure S26. ^1H–^1H COSY spectrum of **3** in CD_3OD (500 MHz)

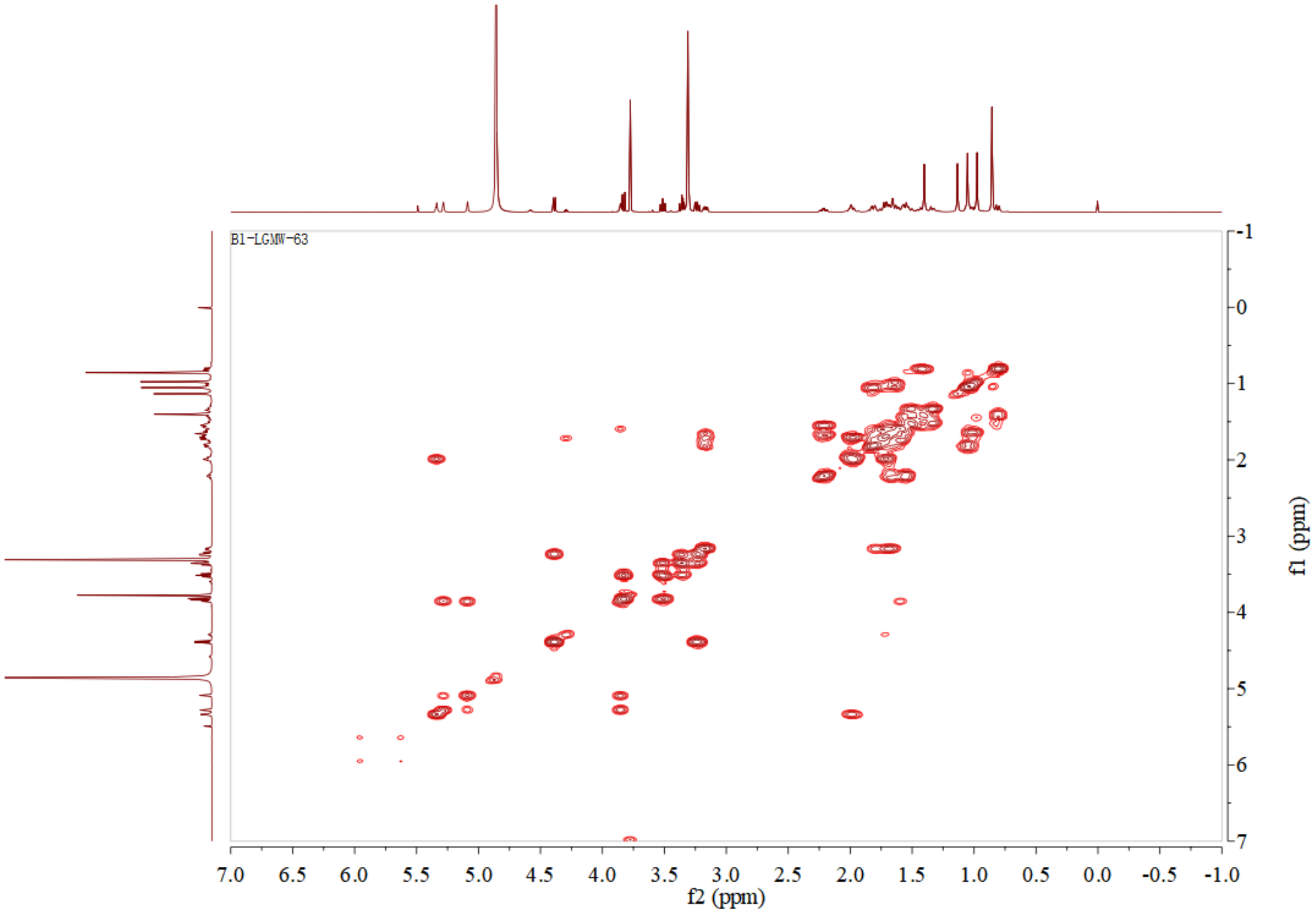

Figure S27. NOESY spectrum of **3** in CD_3OD (500 MHz)

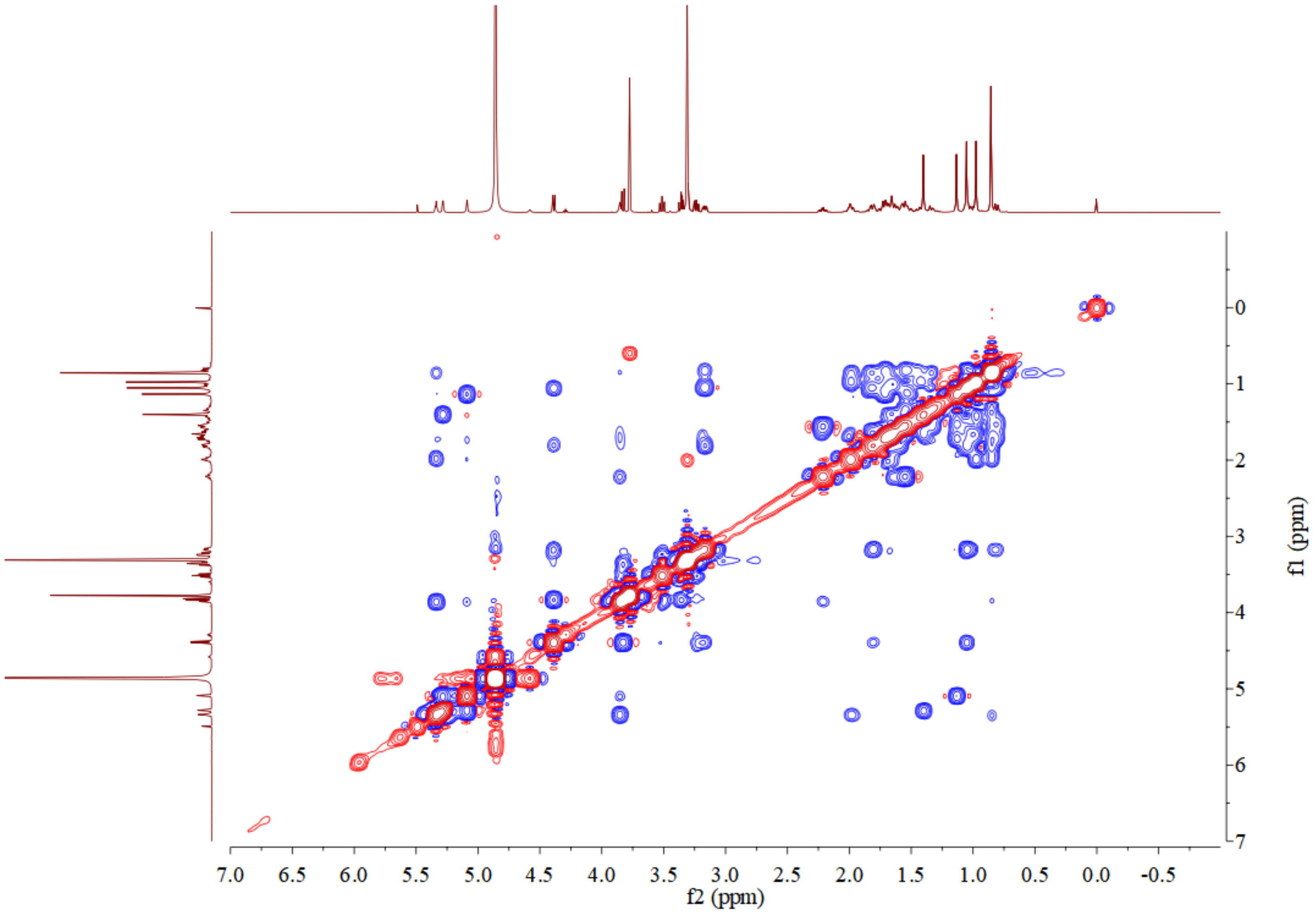

Figure S28. HRESIMS spectrum of **3**

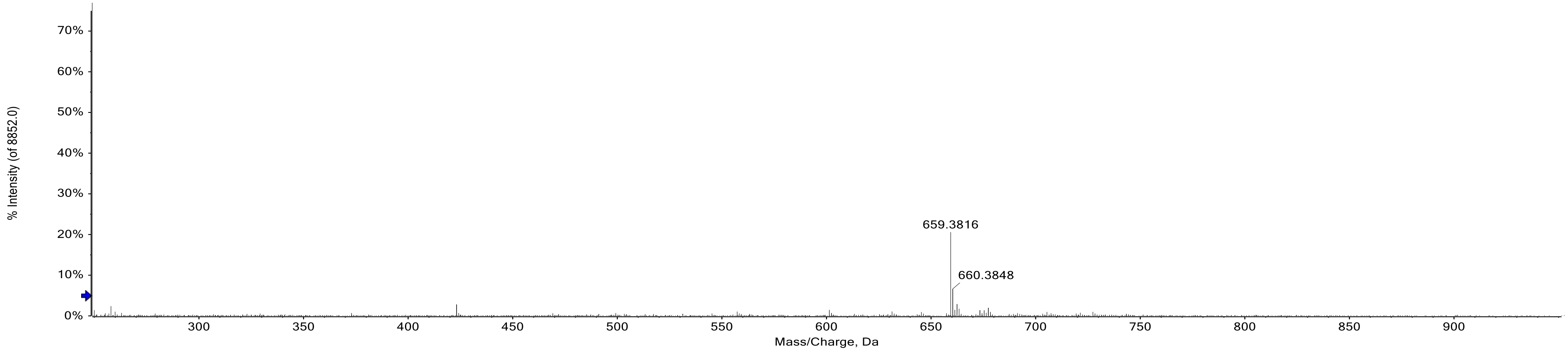

Figure S29. IR (KBr disc) spectrum of **3**

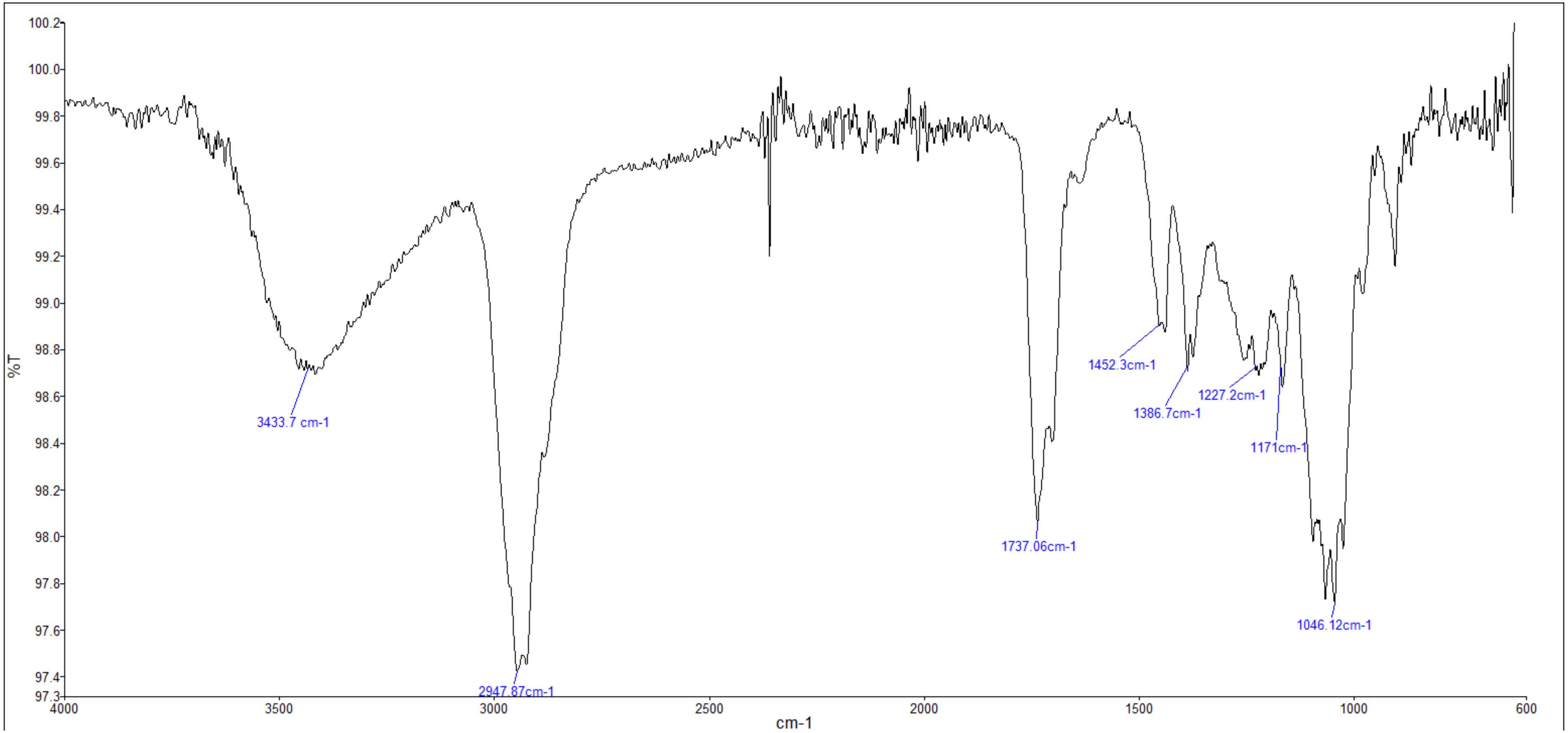

Figure S30. ^{1}H NMR spectrum of **4** in pyridine-d_5 (500 MHz)

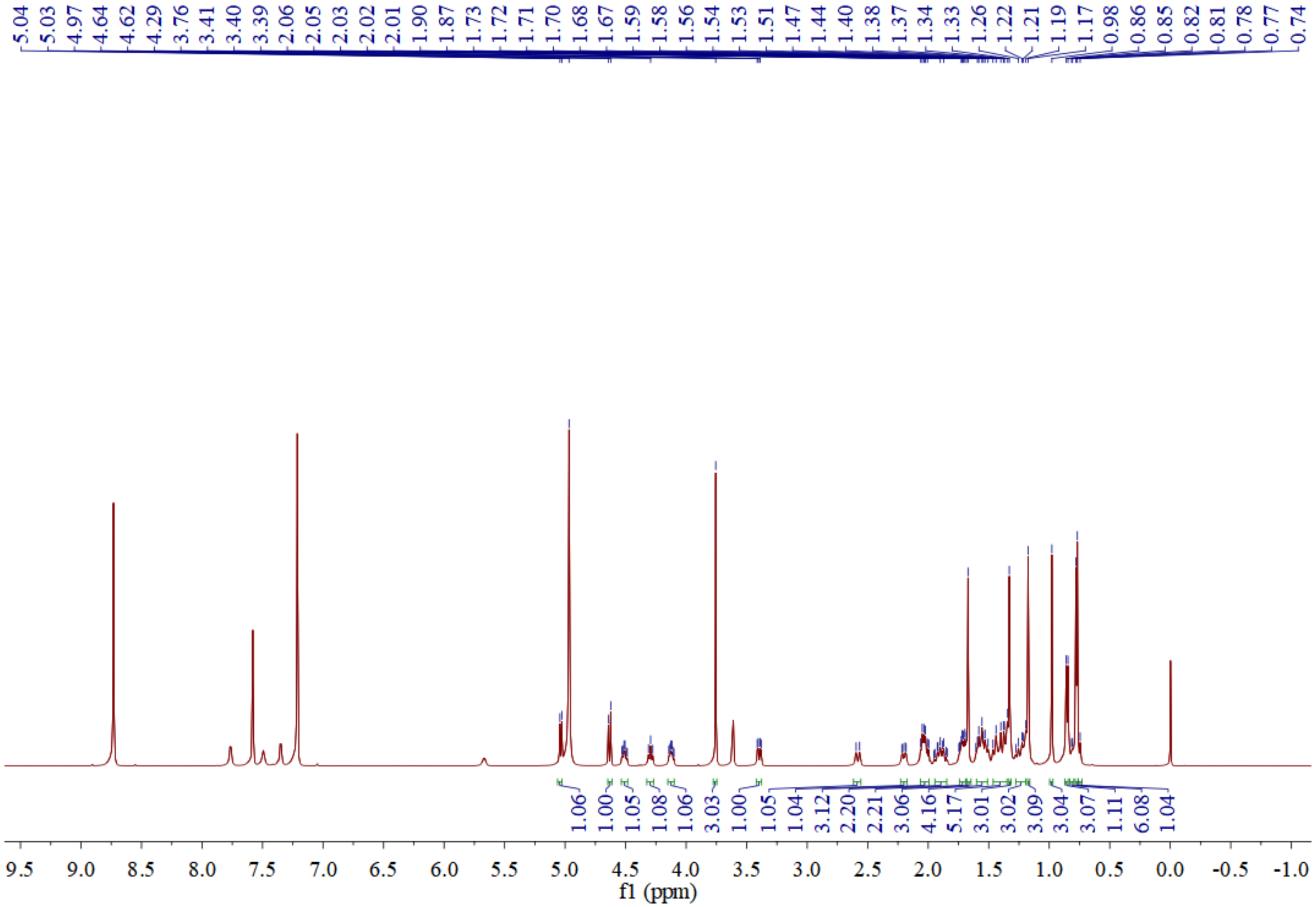

Figure S31. ^{13}C NMR and DEPT spectra of **4** in pyridine-d_5 (125 MHz)

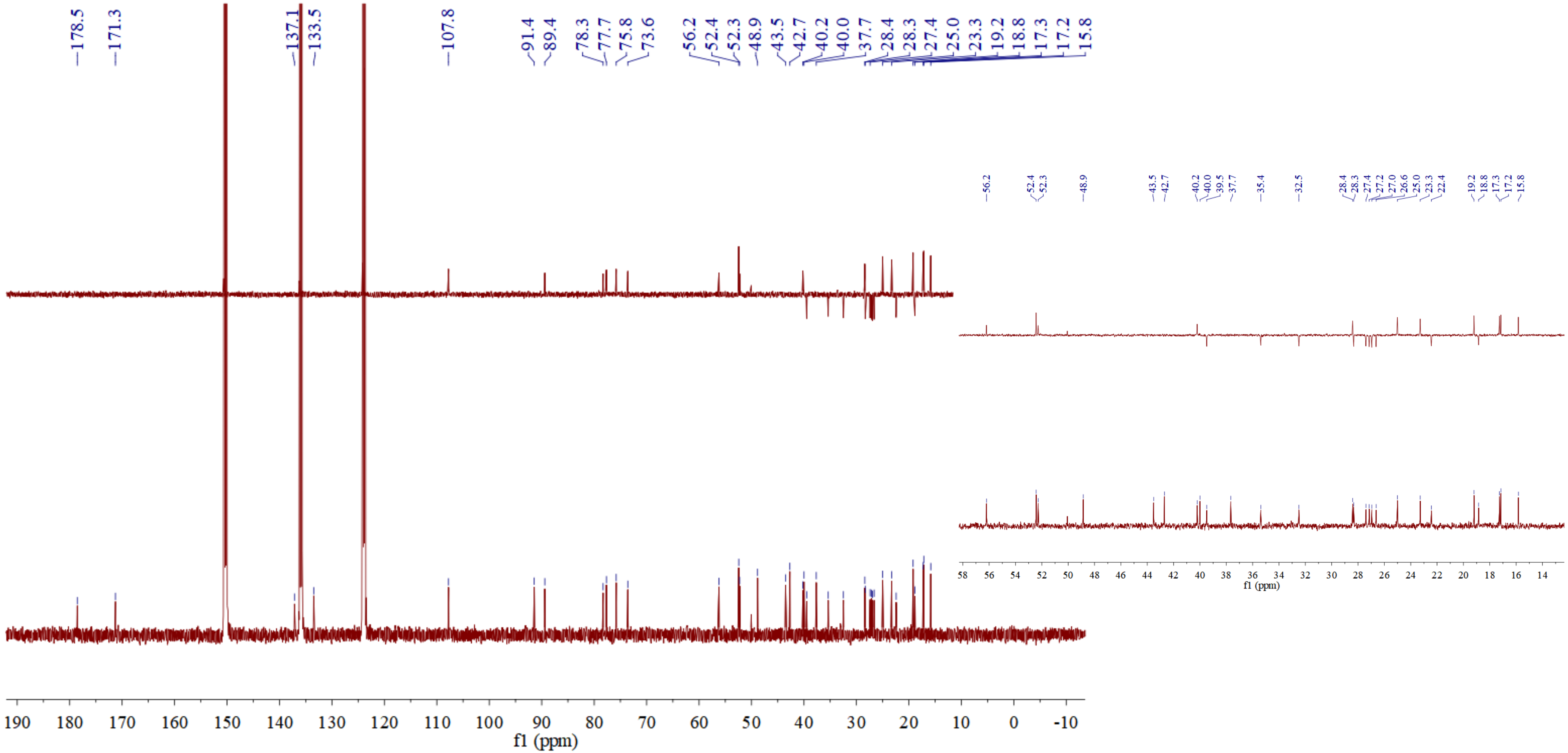

Figure S32. HSQC spectrum of **4** in pyridine-d_5 (500 MHz)

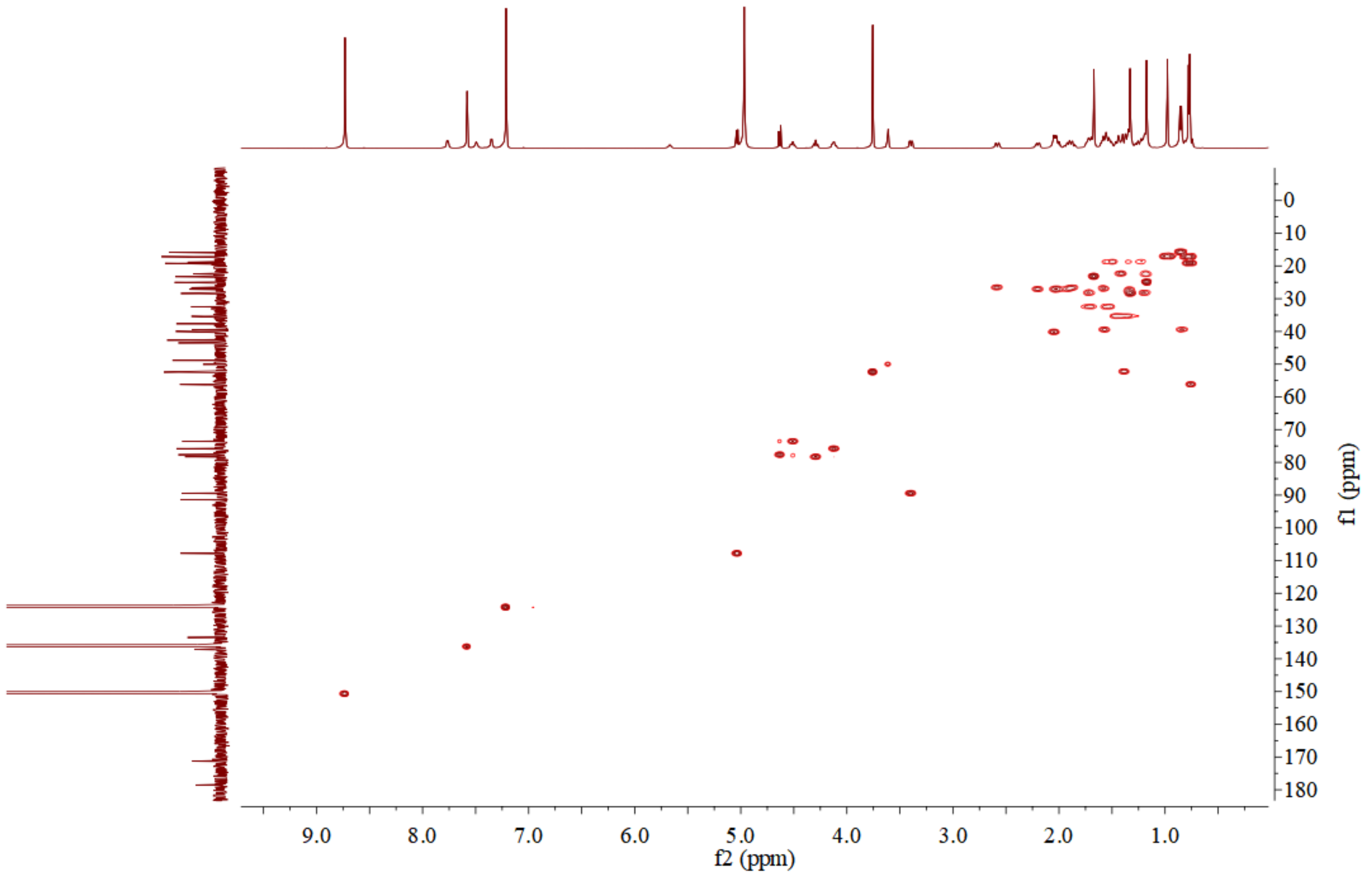

Figure S33. HMBC spectrum of **4** in pyridine-d_5 (500 MHz)

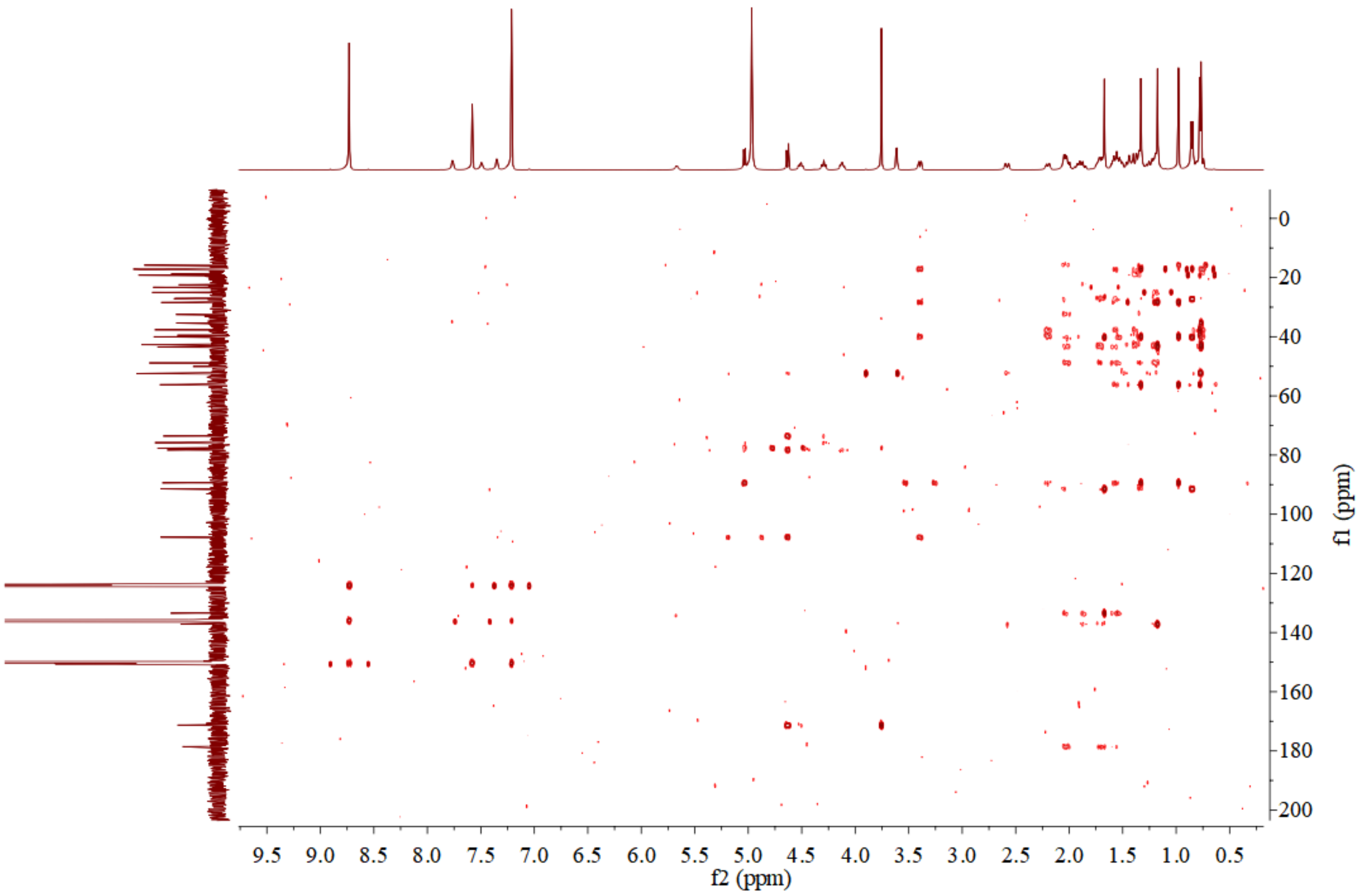

Figure S34. ^{1}H–^{1}H COSY spectrum of **4** in pyridine-d_5 (500 MHz)

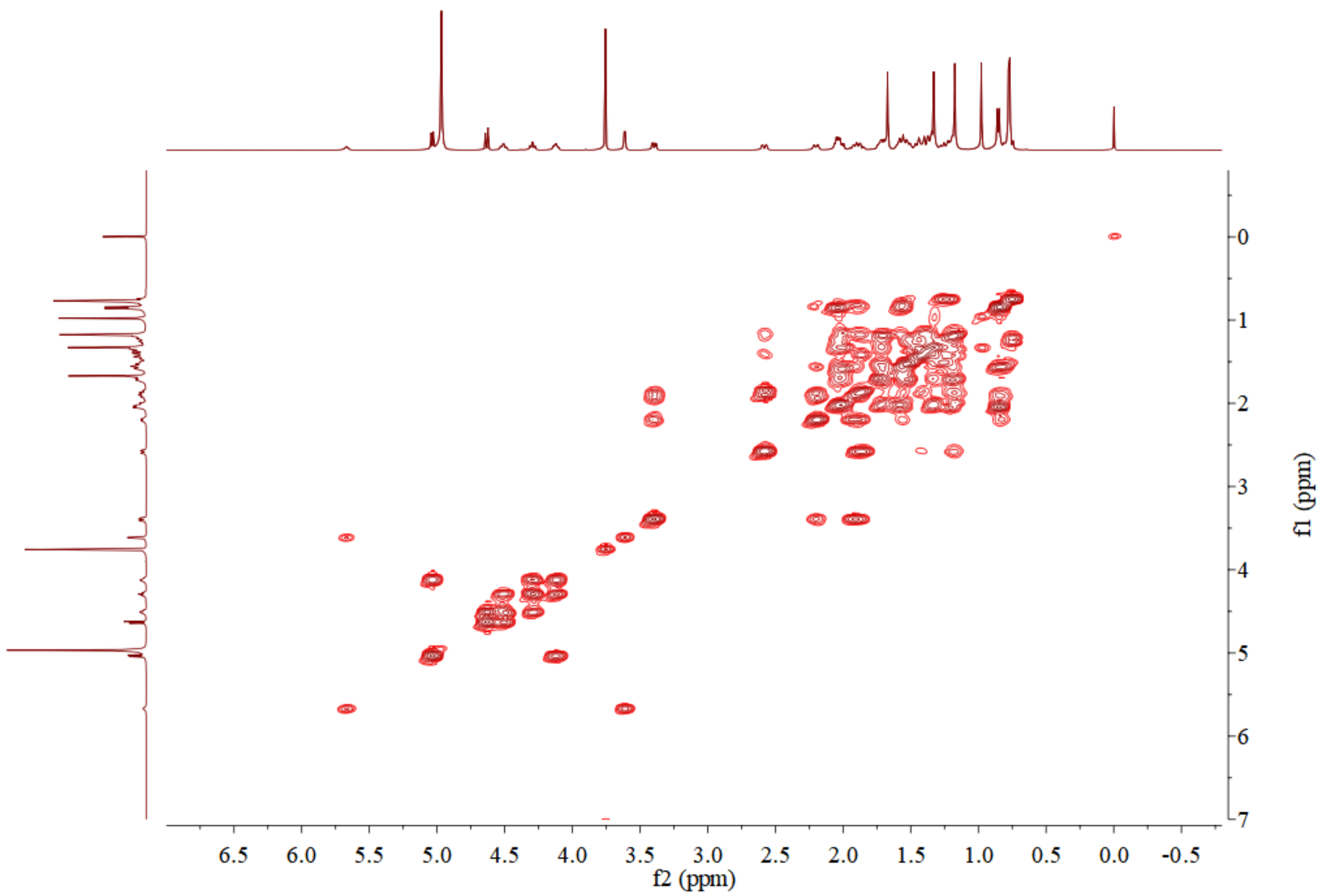

Figure S35. NOESY spectrum of **4** in pyridine-d_5 (500 MHz)

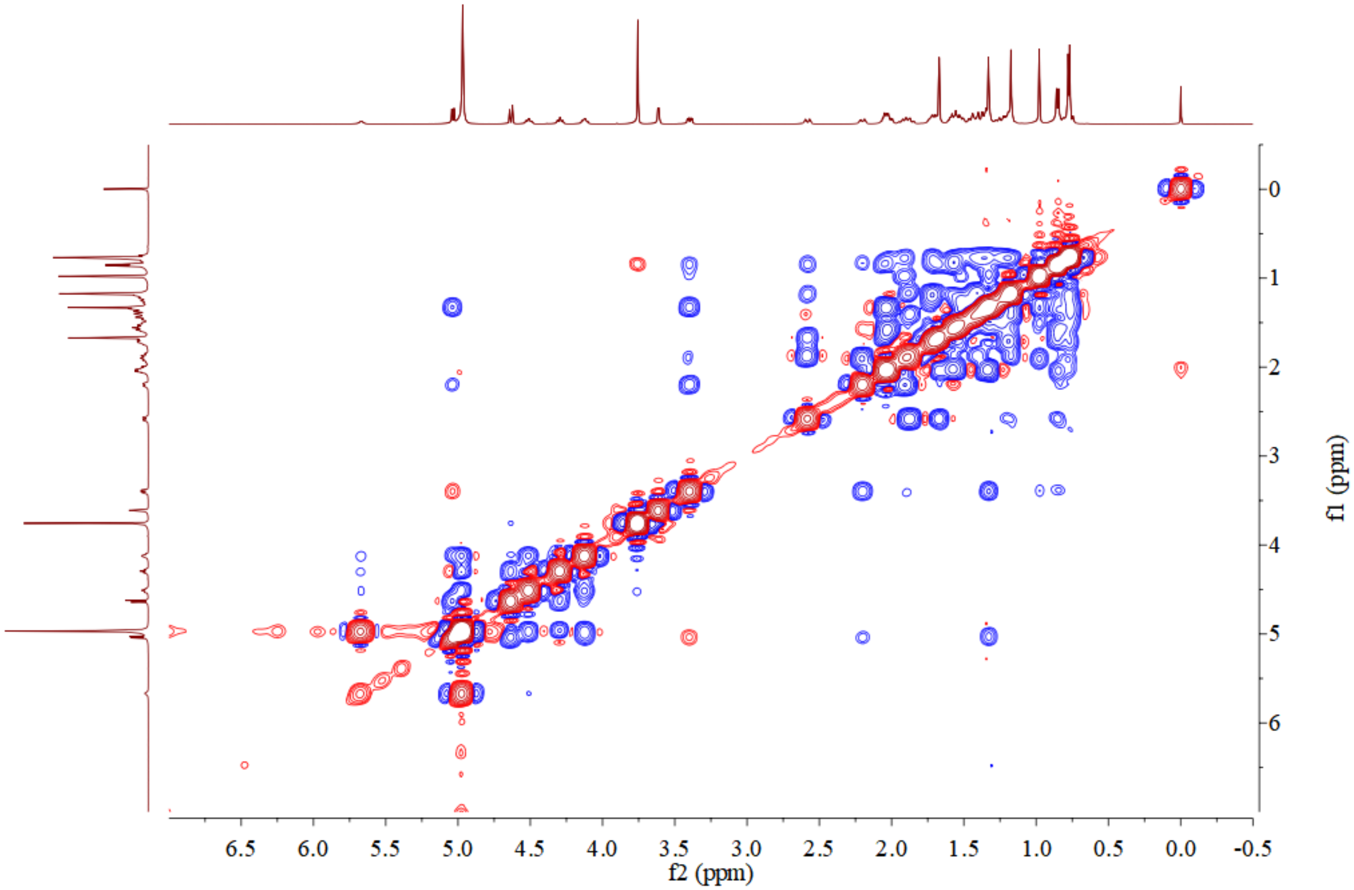

Figure S36. HRESIMS spectrum of **4**

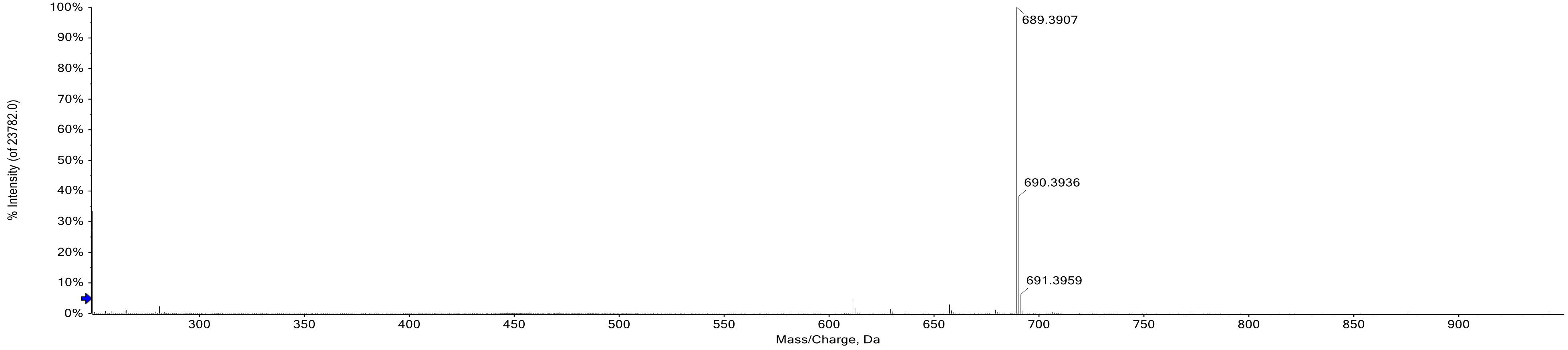

Figure S37. IR (KBr disc) spectrum of **4**

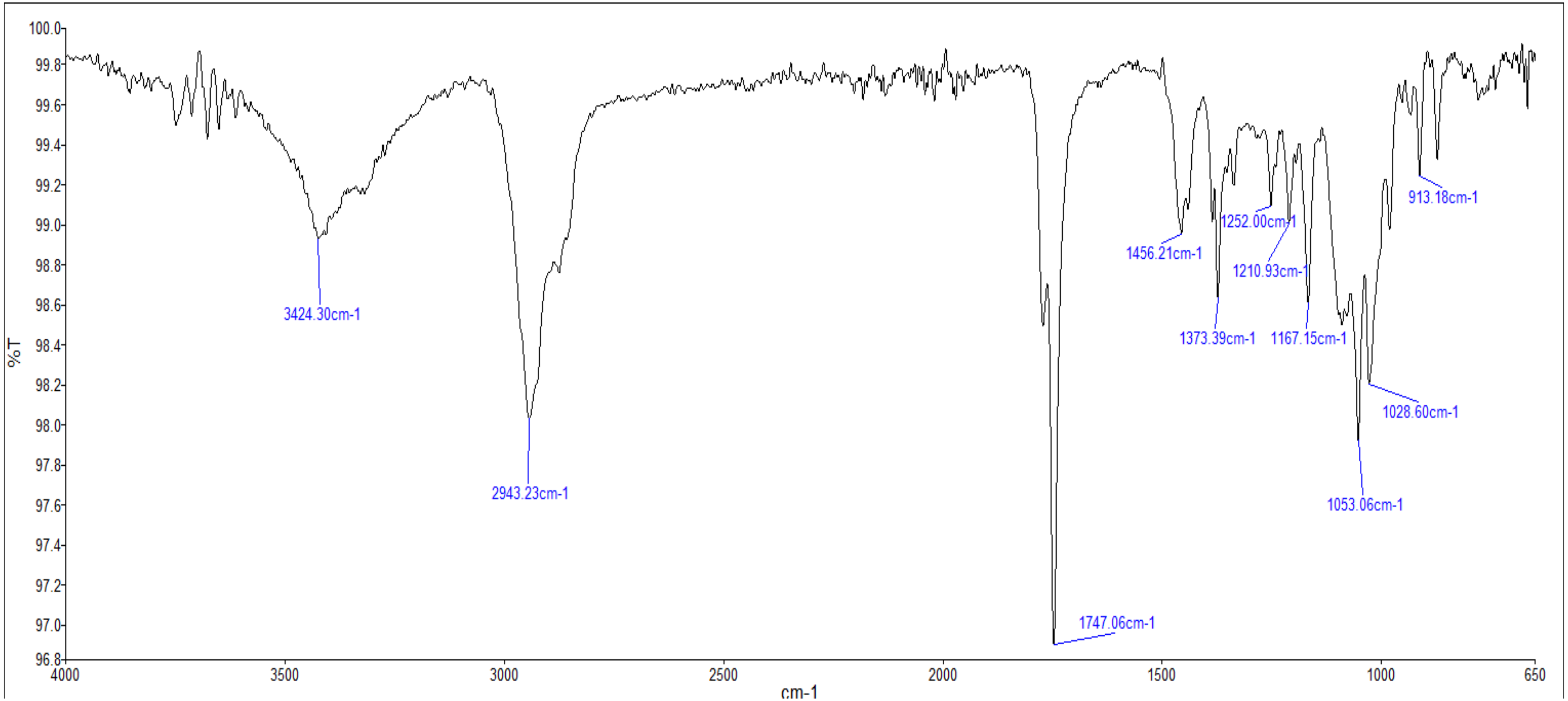

Figure S38. ¹H NMR spectrum of **5** in pyridine-d_5 (500 MHz)

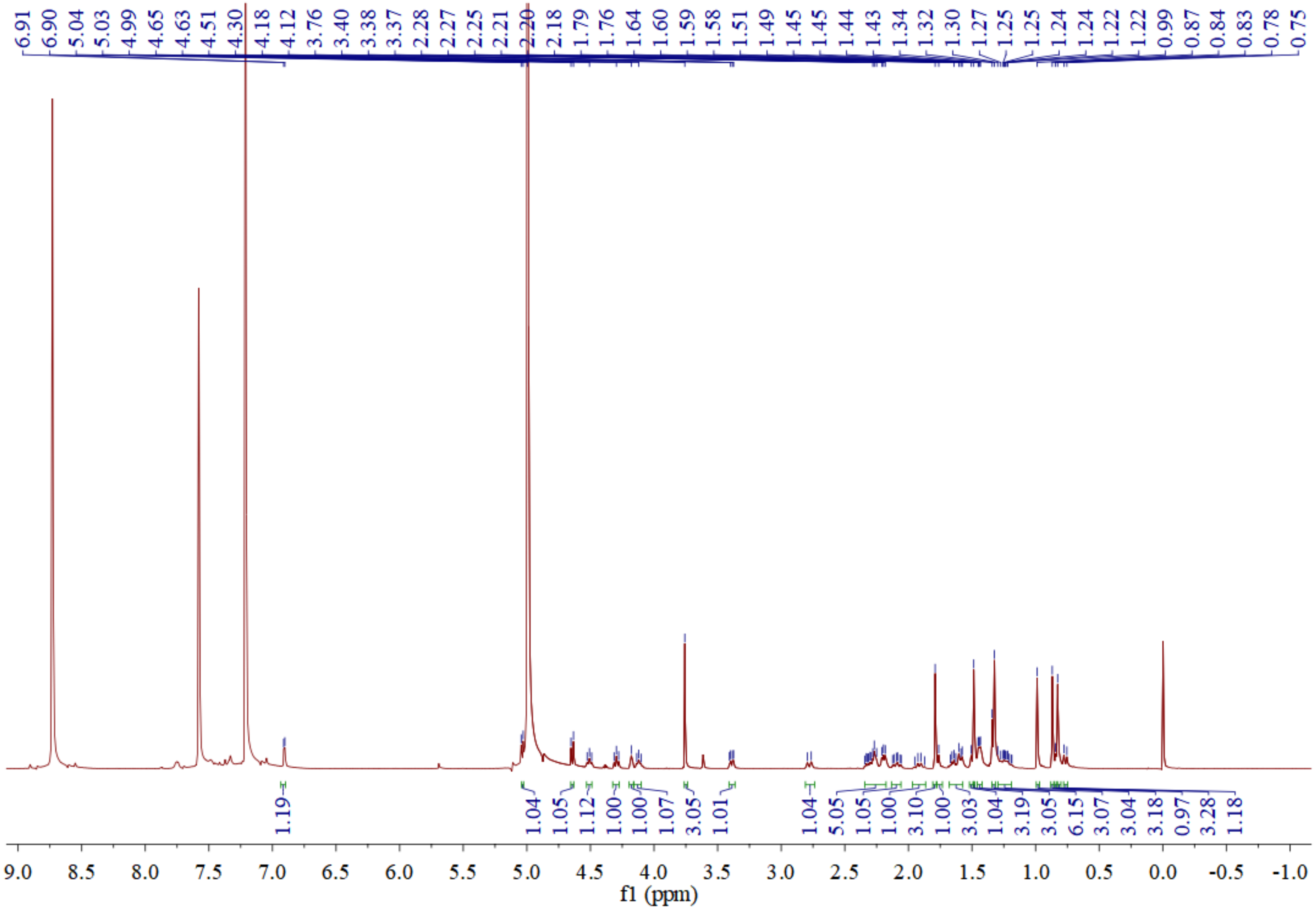

Figure S39. ^{13}C NMR and DEPT spectra of **5** in pyridine-d_5 (125 MHz)

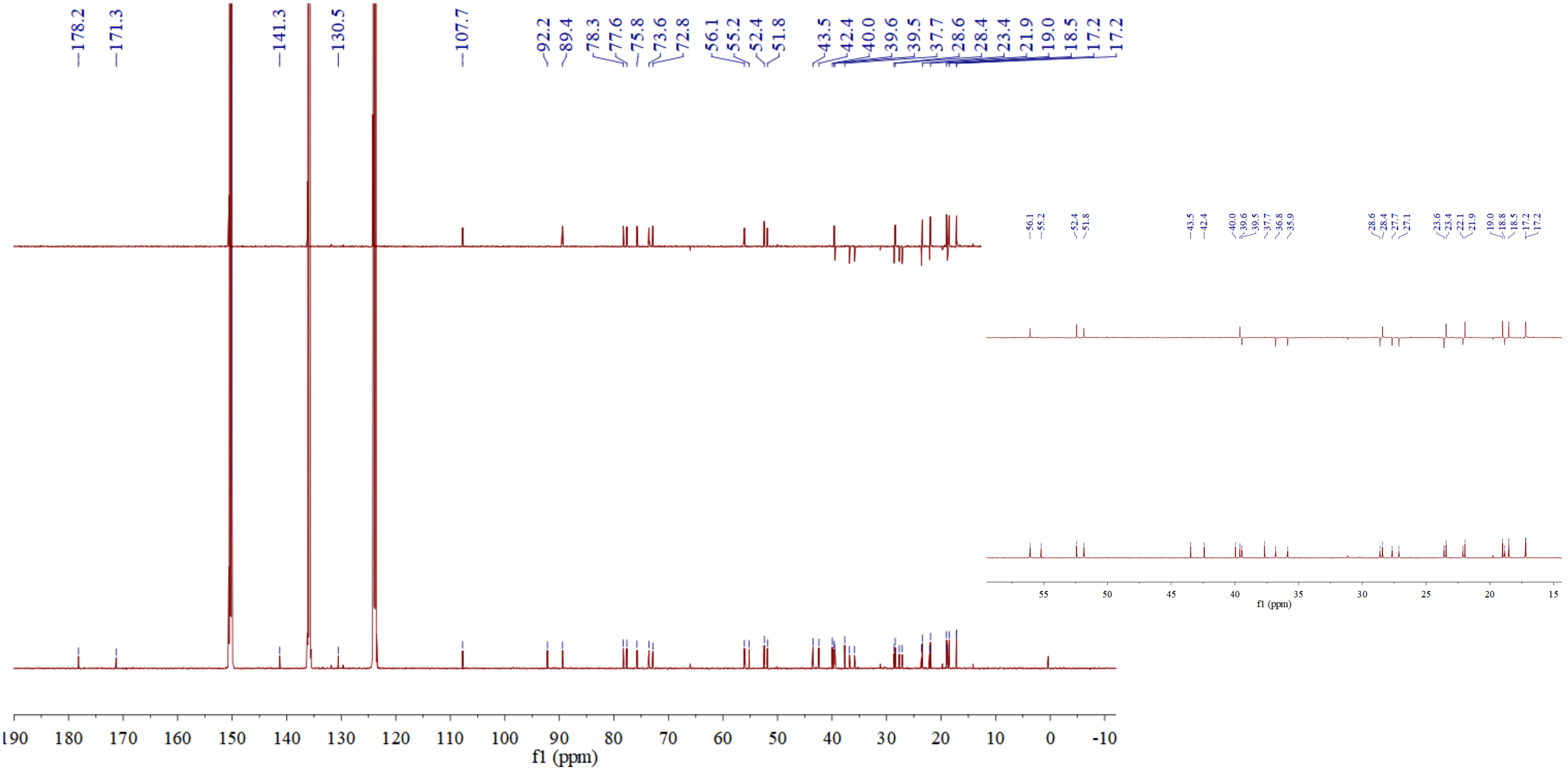

Figure S40. HSQC spectrum of **5** in pyridine-d_5 (500 MHz)

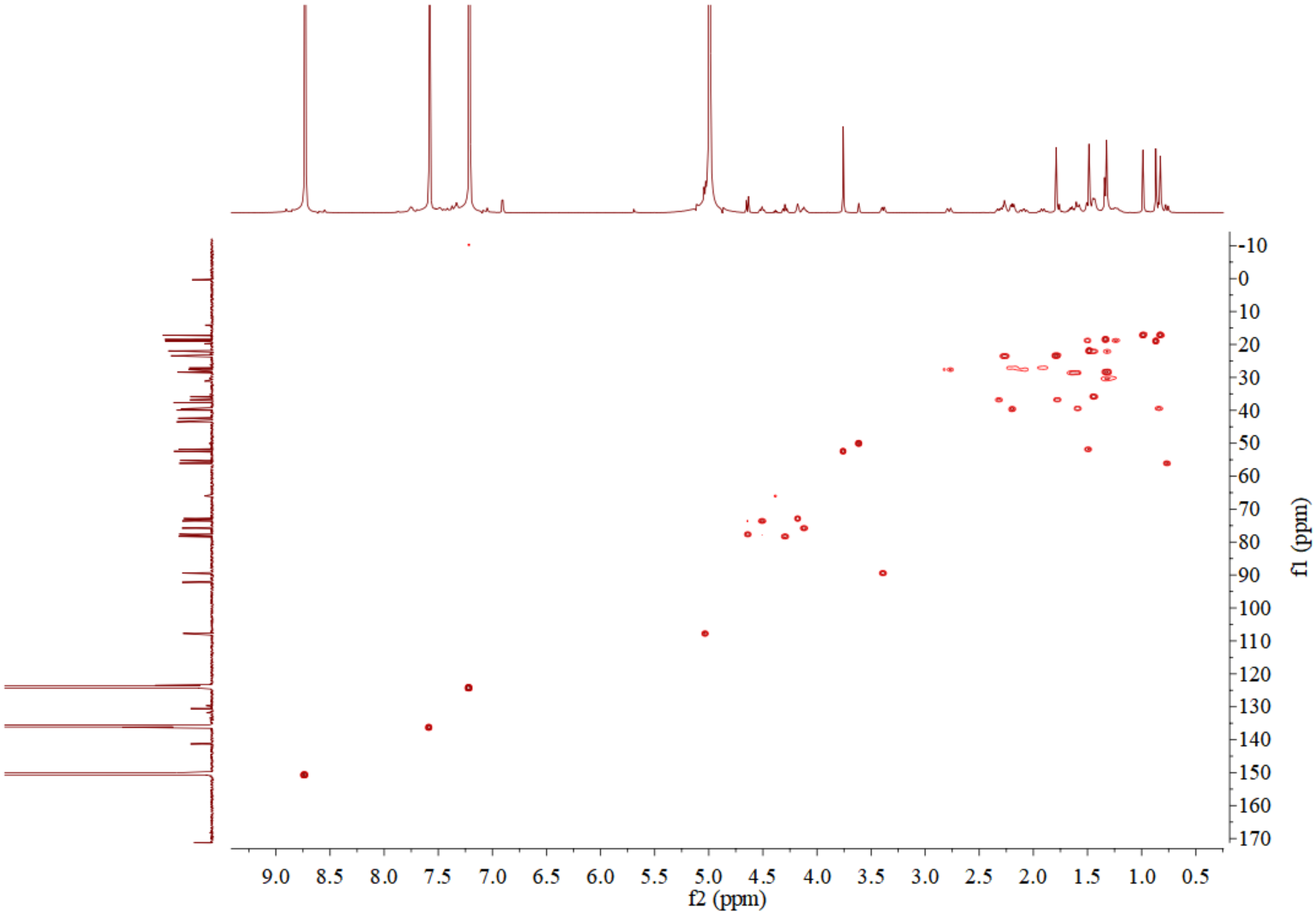

Figure S41. HMBC spectrum of **5** in pyridine-d_5 (500 MHz)

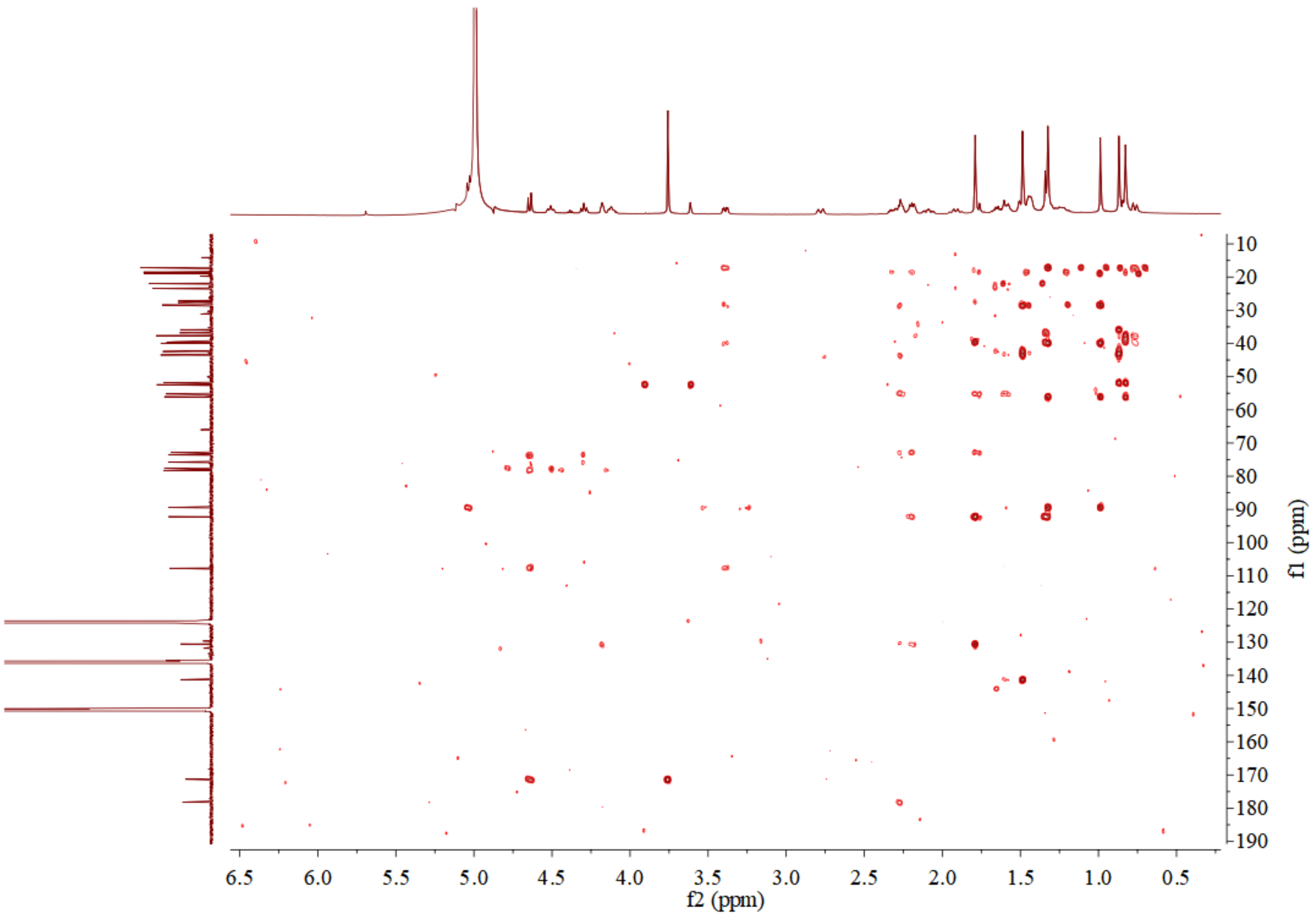

Figure S42. ^1H–^1H COSY spectrum of **5** in pyridine-d_5 (500 MHz)

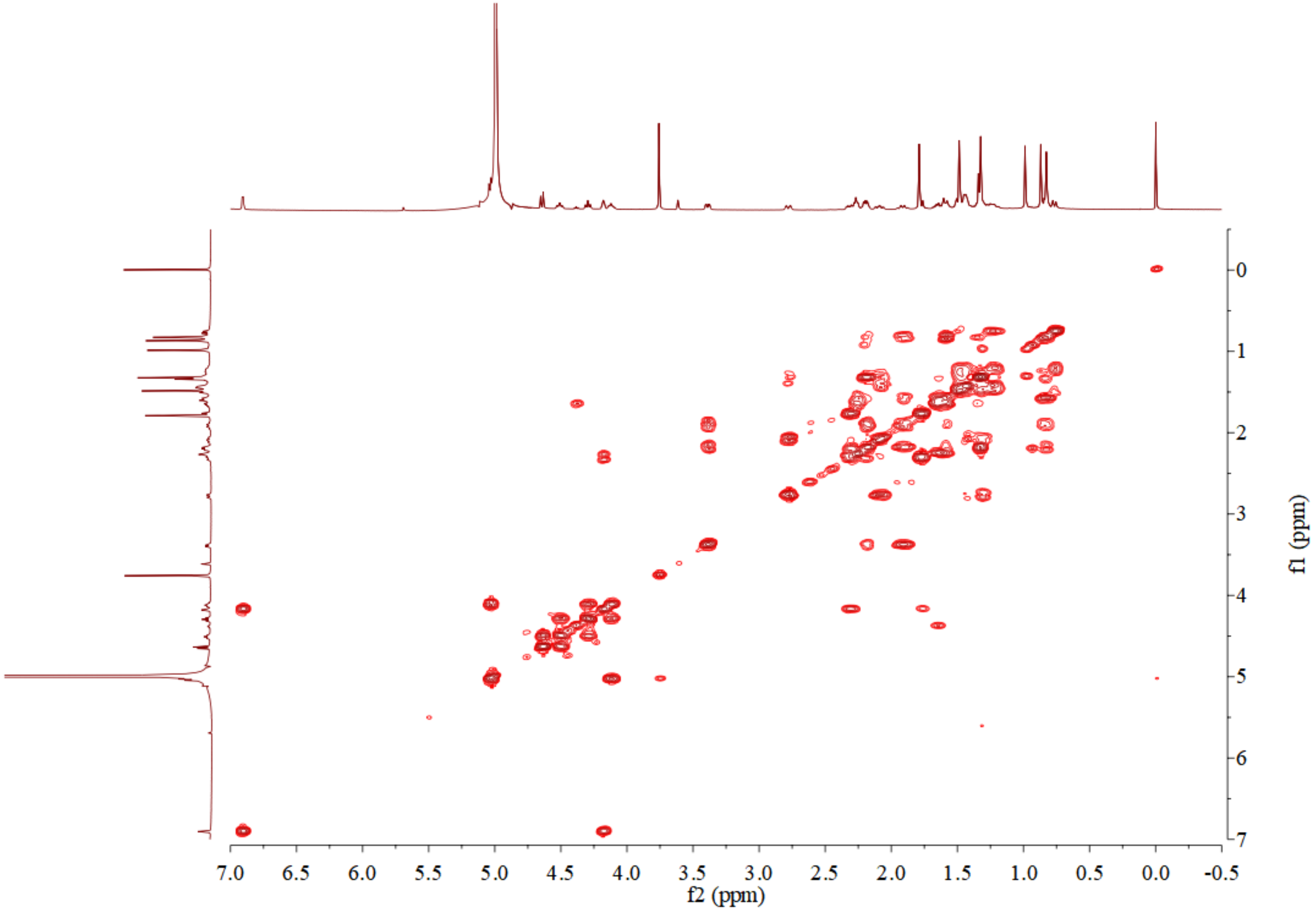

Figure S43. NOESY spectrum of **5** in pyridine-d_5 (500 MHz)

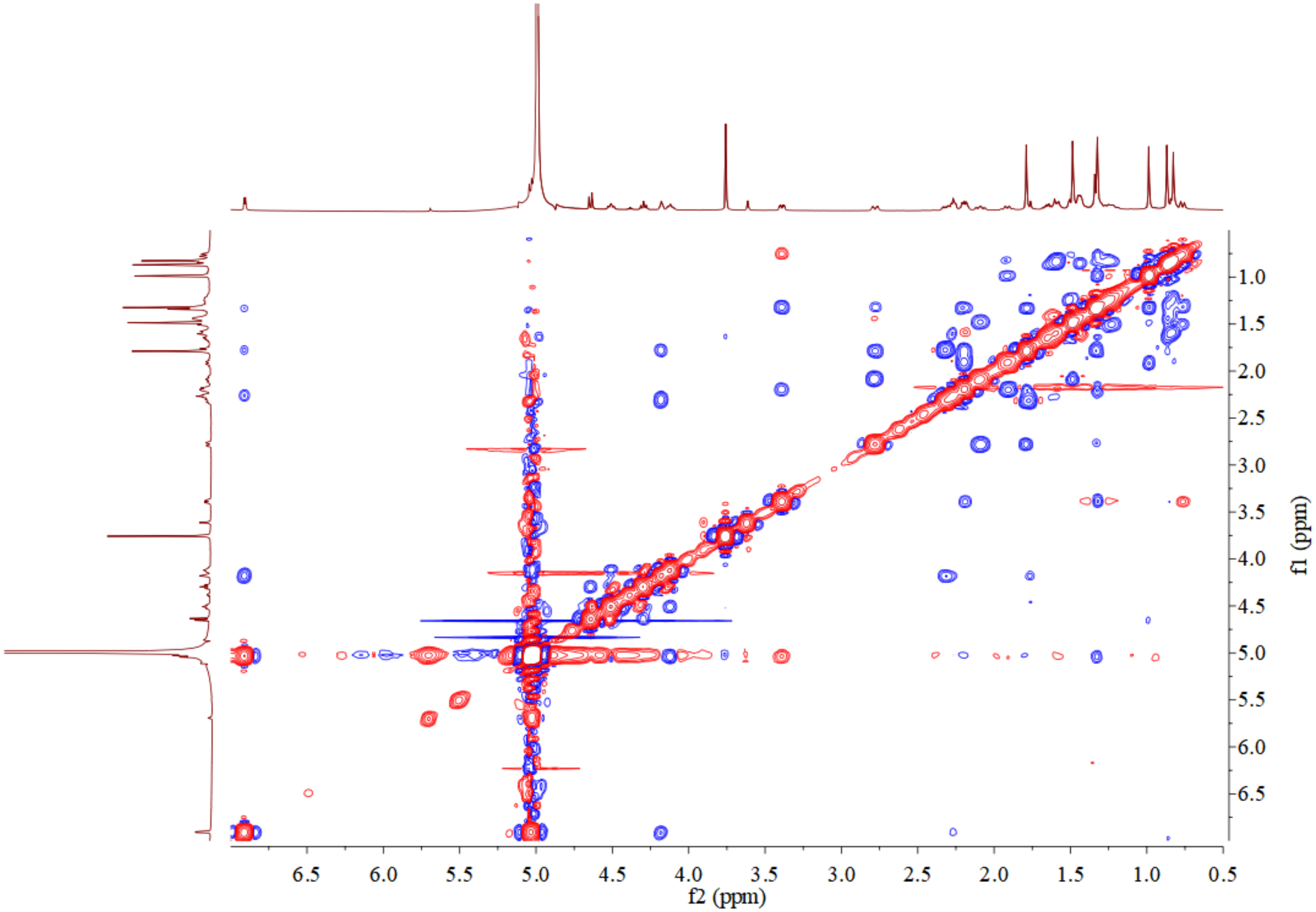

Figure S44. HRESIMS spectrum of **5**

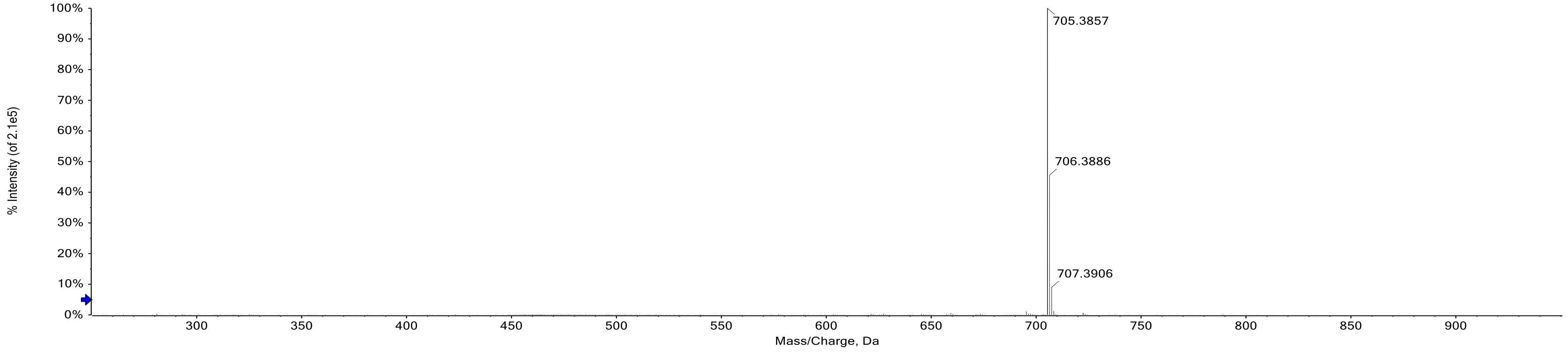

Figure S45. IR (KBr disc) spectrum of **5**

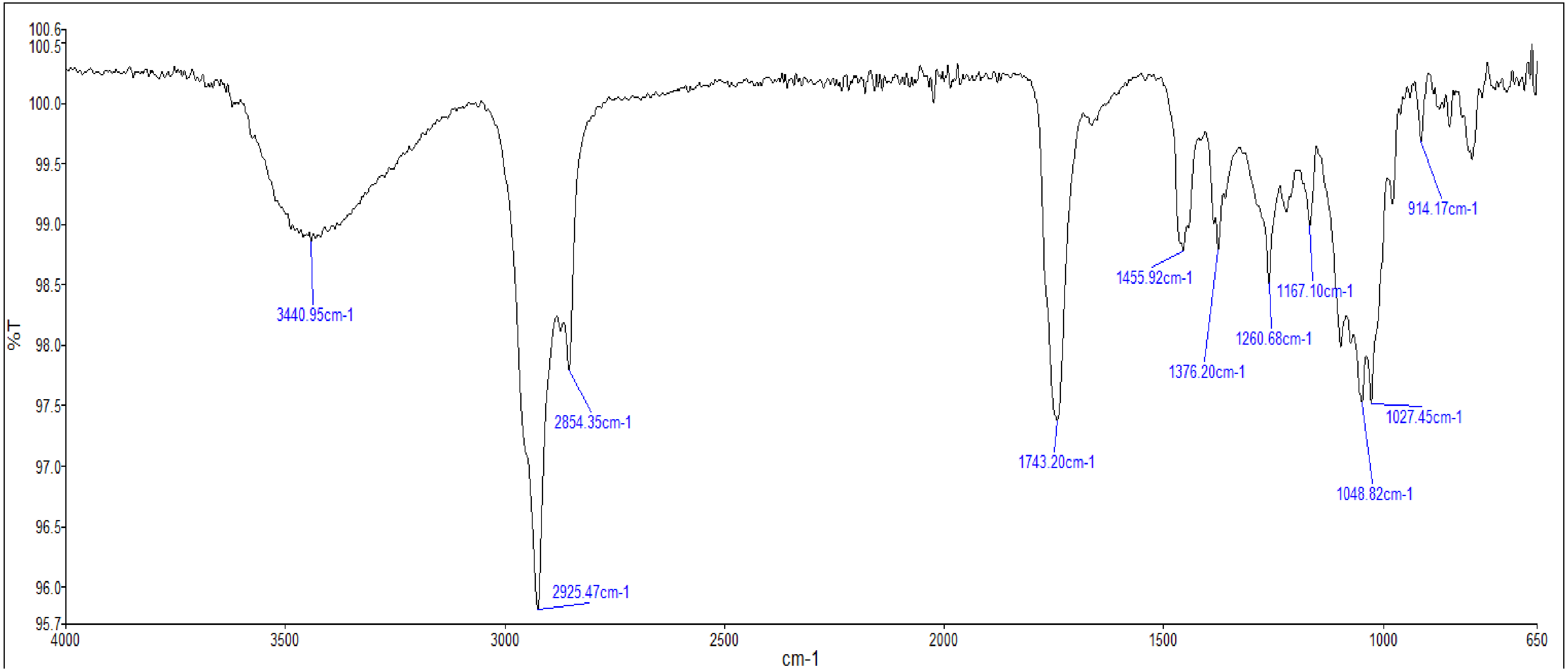

Figure S46. ^{1}H NMR spectrum of **6** in pyridine-d_5 (500 MHz)

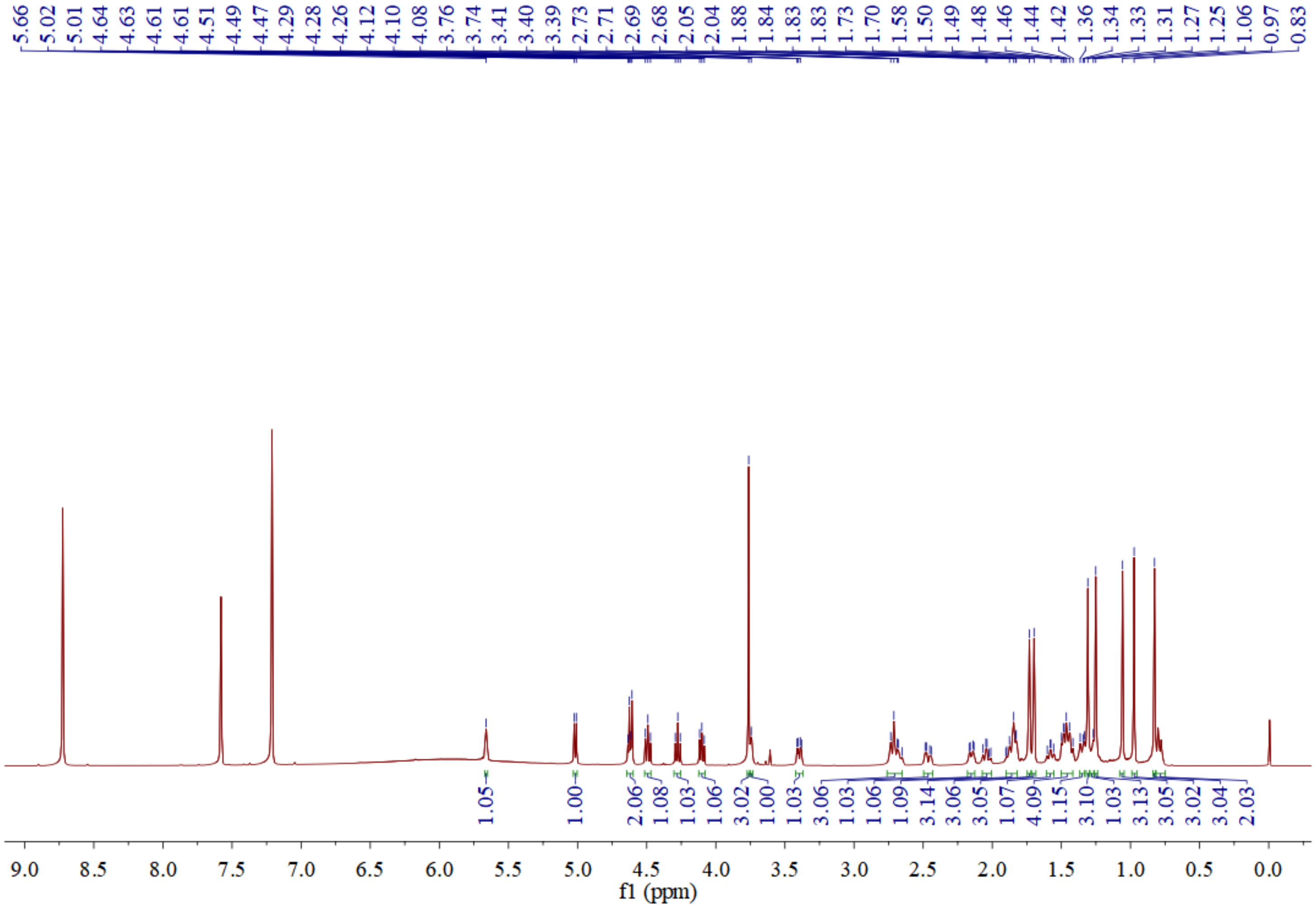

Figure S47. ^{13}C NMR and DEPT spectra of **6** in pyridine-d_5 (125 MHz)

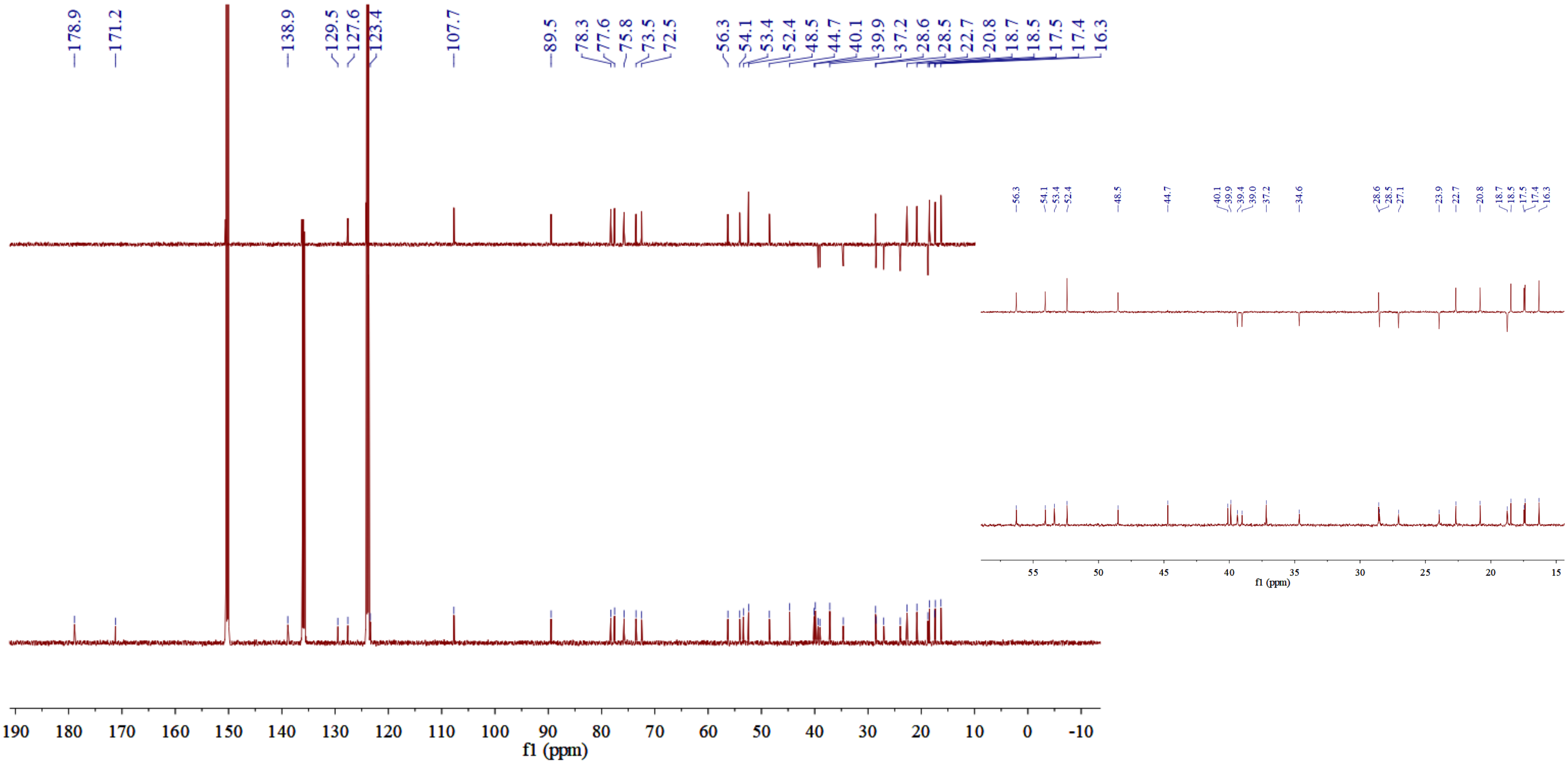

Figure S48. HSQC spectrum of **6** in pyridine-*d_5* (500 MHz)

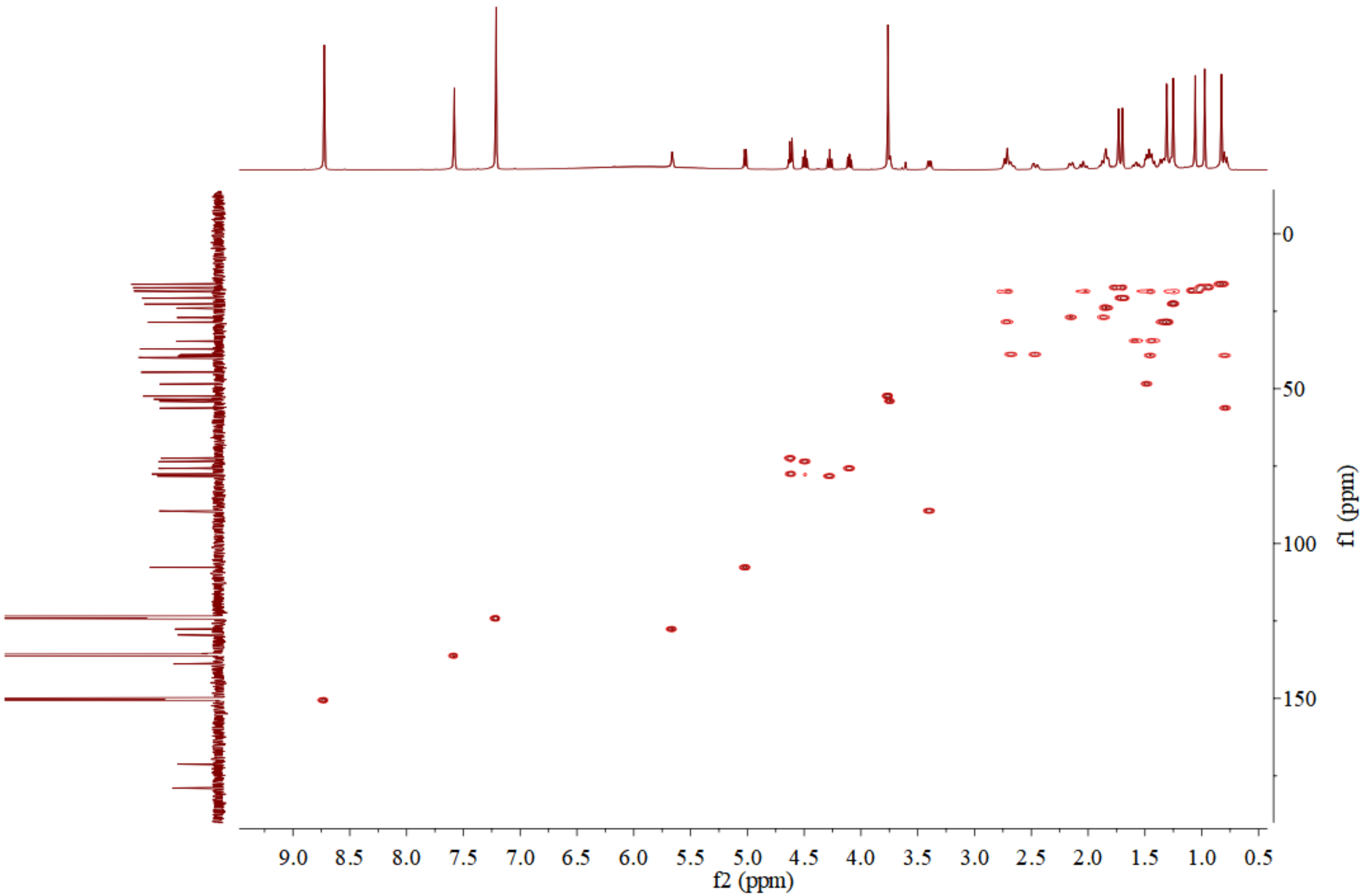

Figure S49. HMBC spectrum of **6** in pyridine-d_5 (500 MHz)

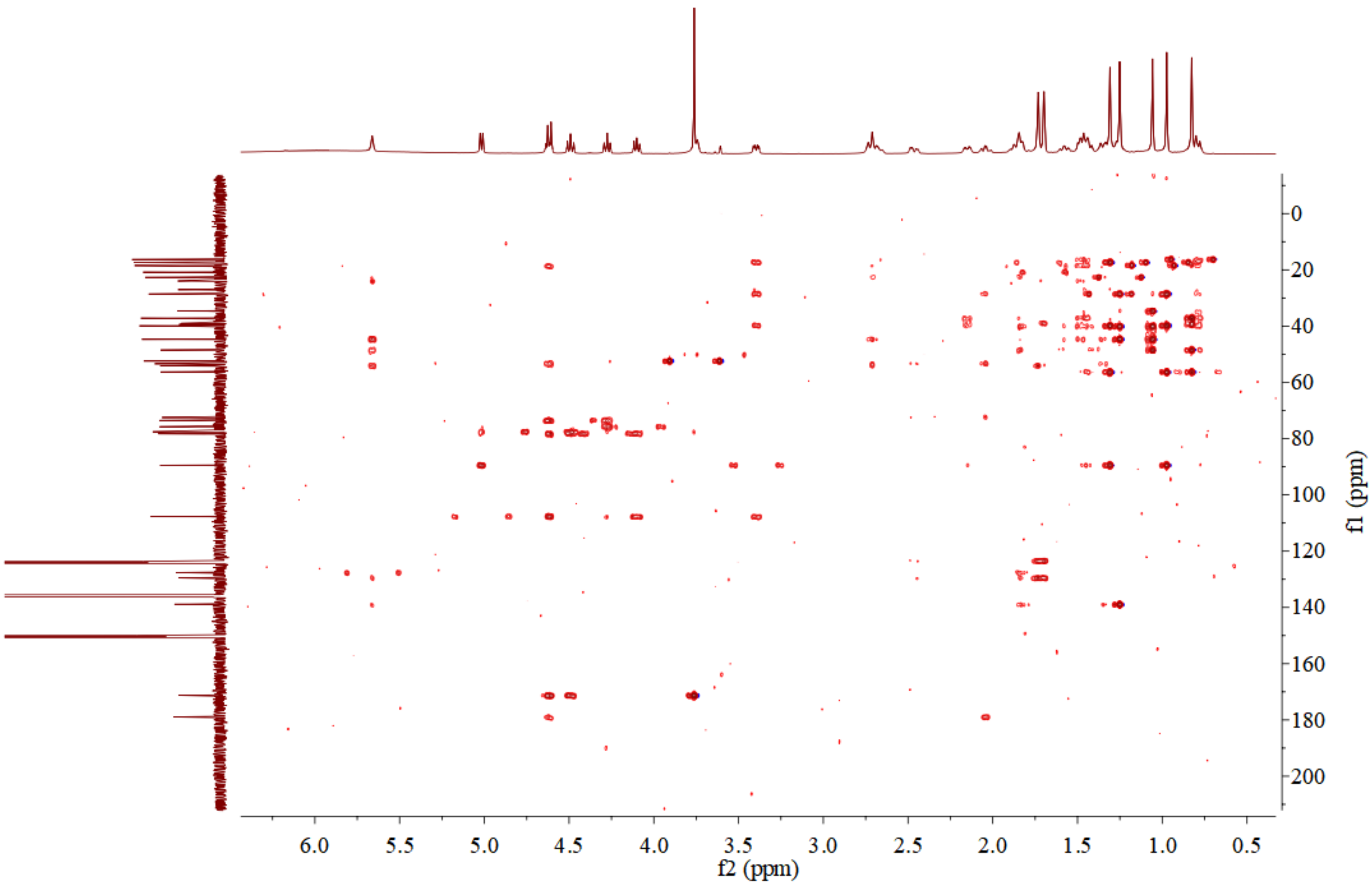

Figure S50. ^1H–^1H COSY spectrum of **6** in pyridine-d_5 (500 MHz)

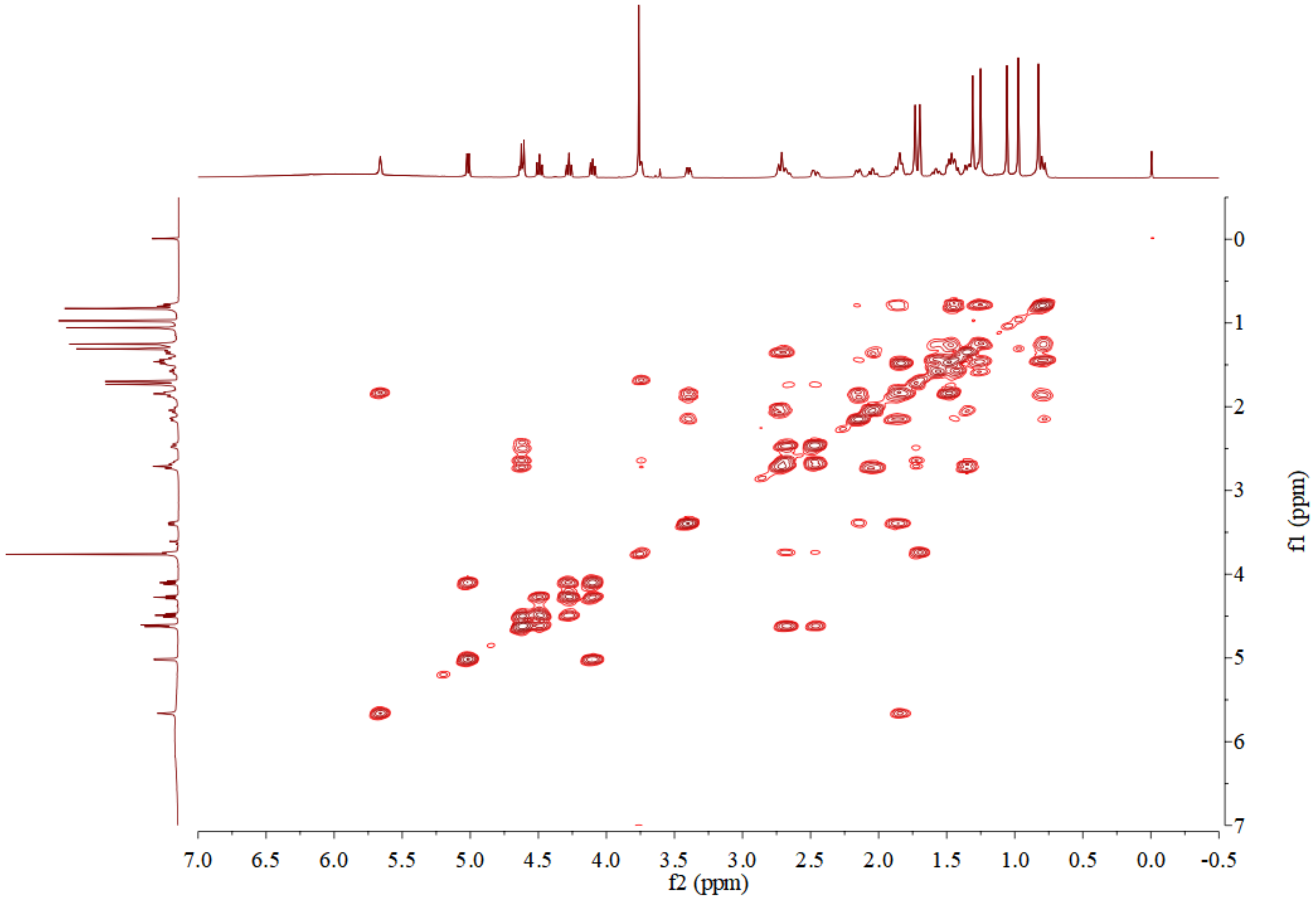

Figure S51. NOESY spectrum of **6** in pyridine-d_5 (500 MHz)

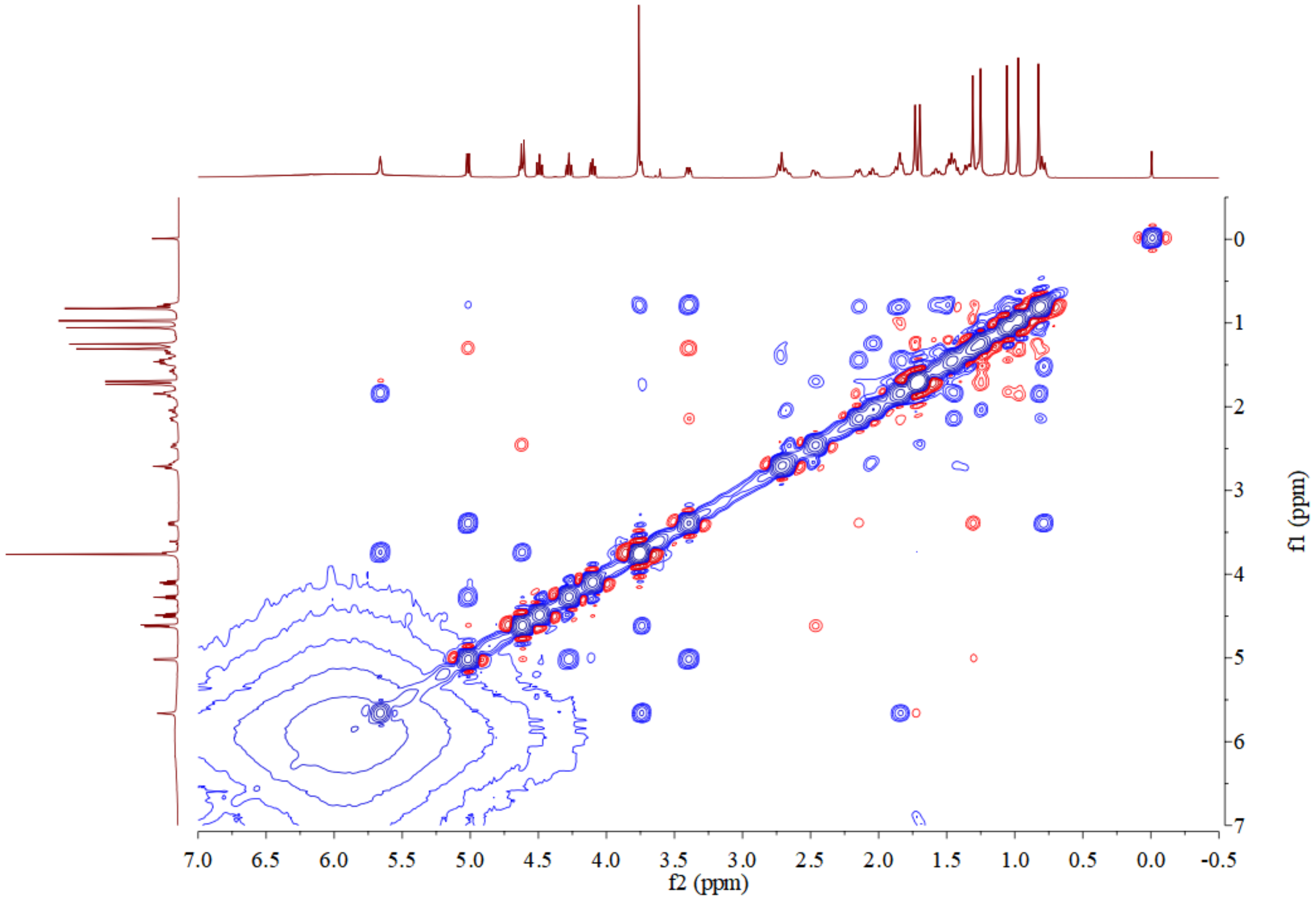

Figure S52. HRESIMS spectrum of **6**

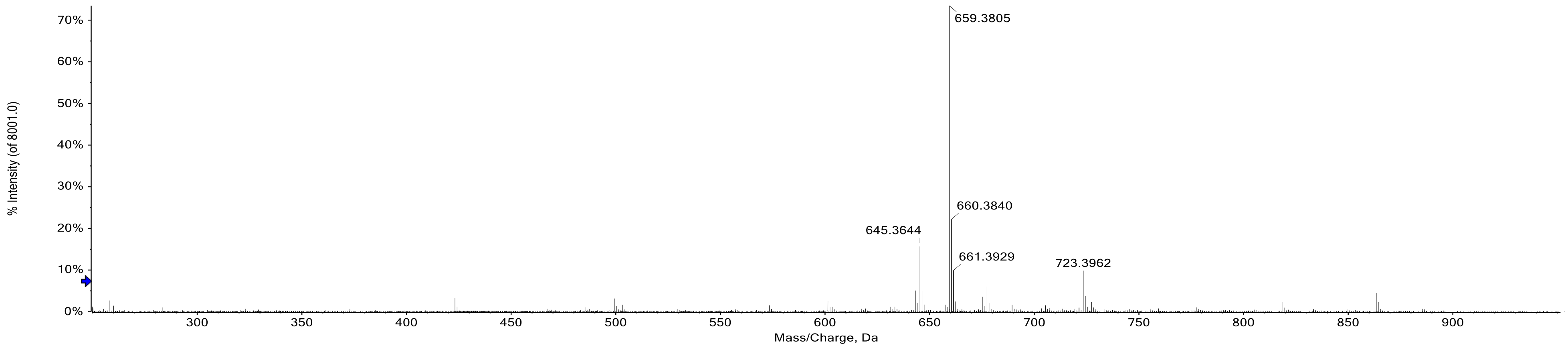

Figure S53. IR (KBr disc) spectrum of **6**

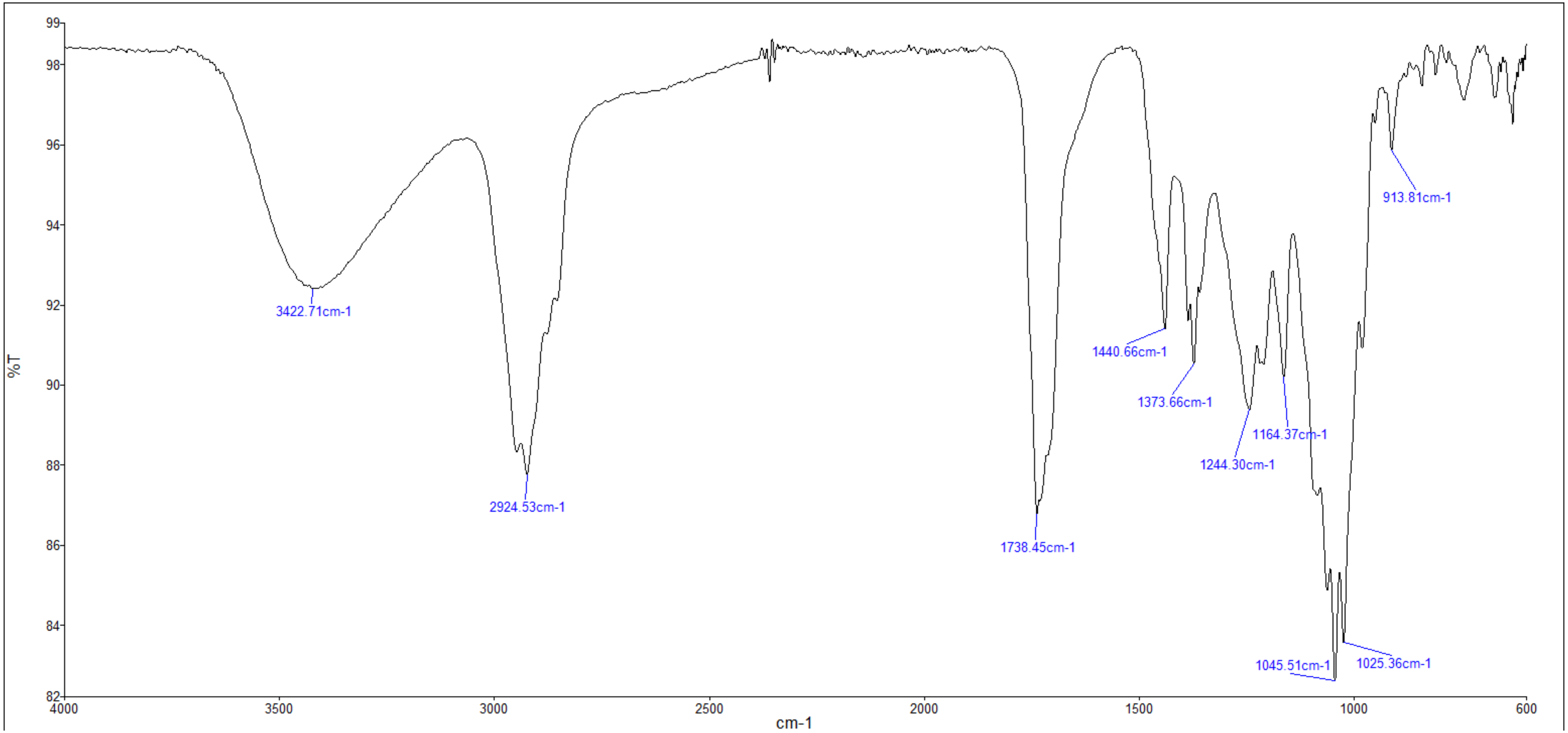

Figure S54. ^{1}H NMR spectrum of **7** in pyridine-d_5 (500 MHz)

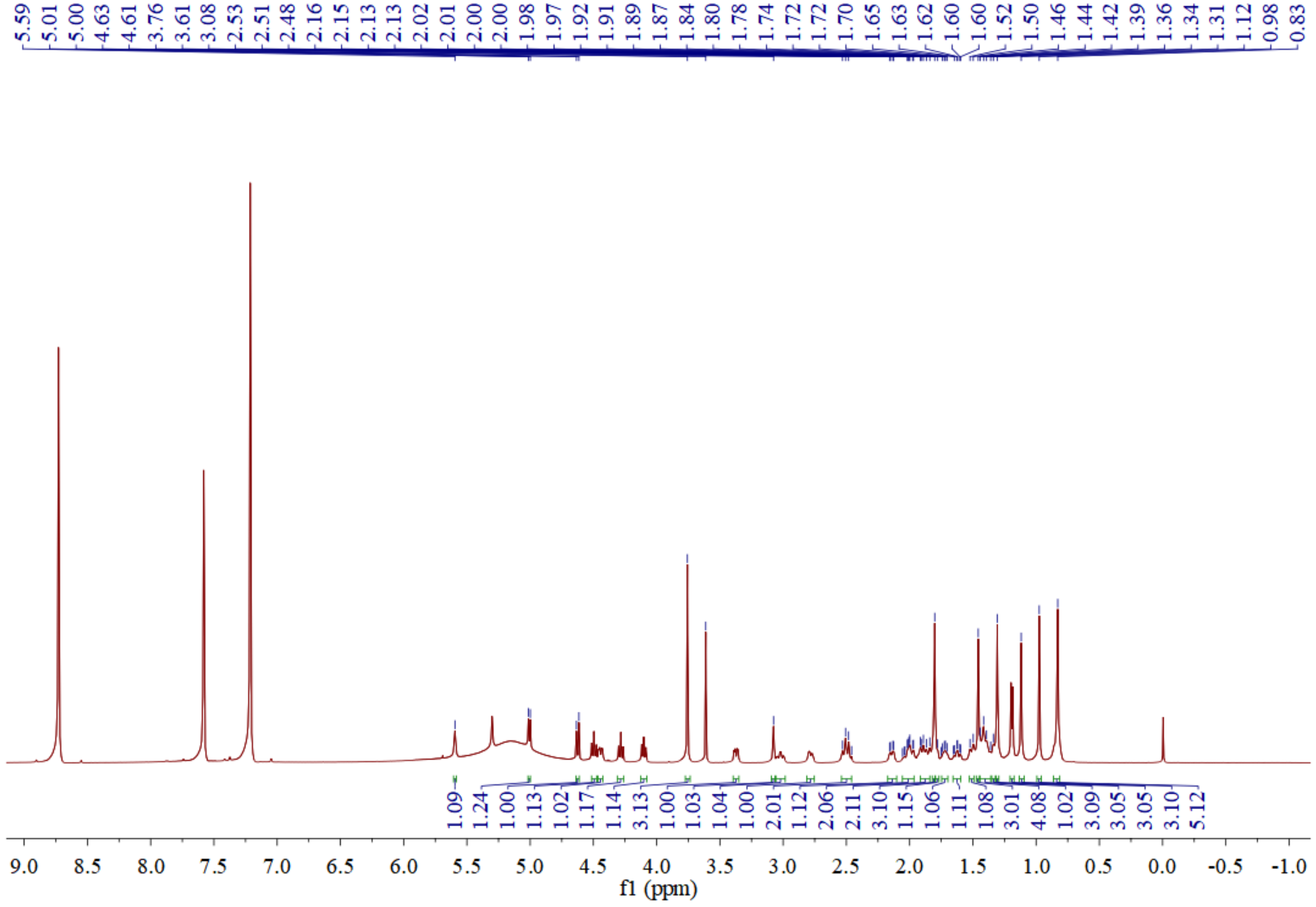

Figure S55. ^{13}C NMR and DEPT spectra of **7** in pyridine-d_5 (125 MHz)

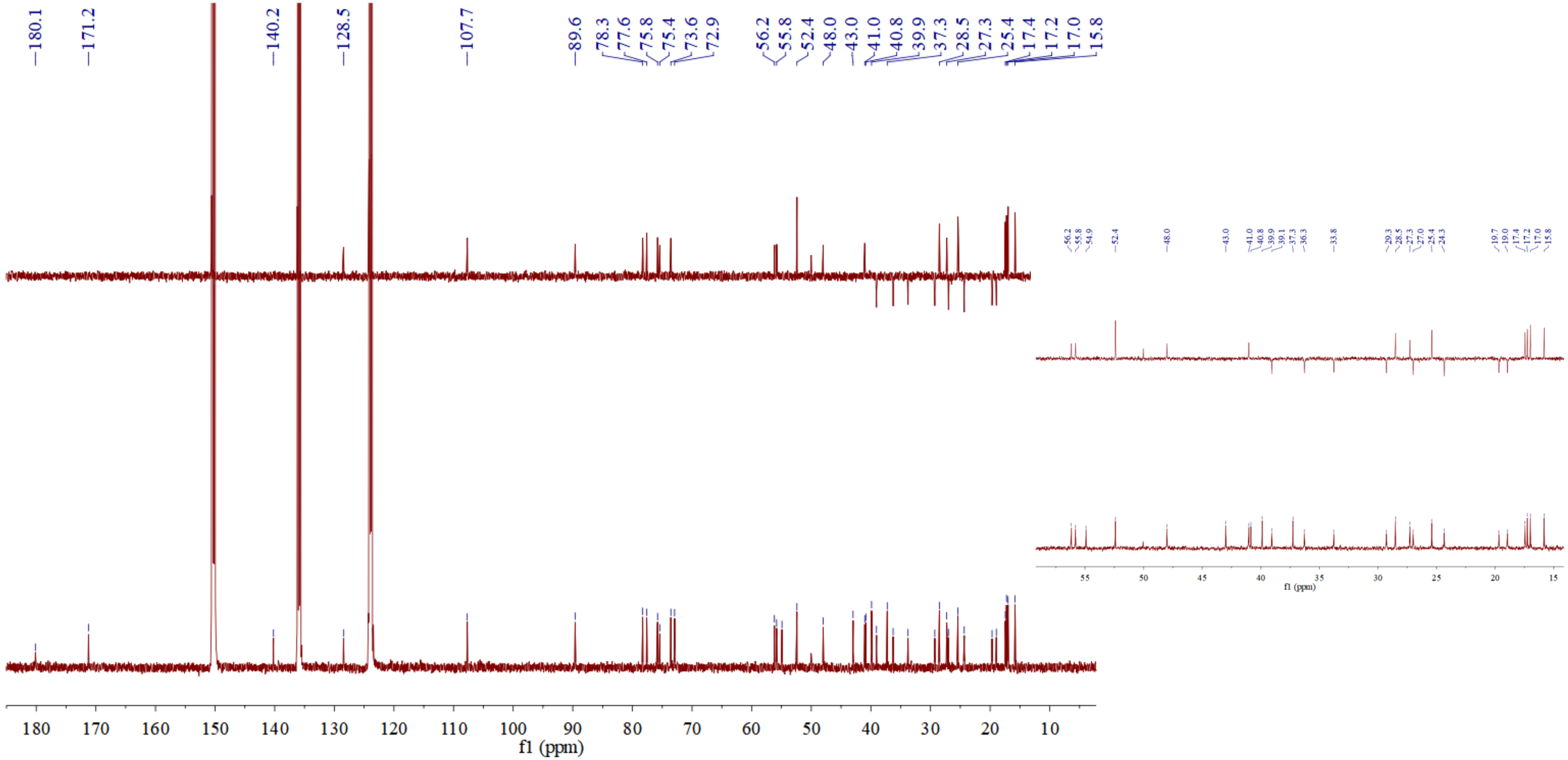

Figure S56. HSQC spectrum of **7** in pyridine-d_5 (500 MHz)

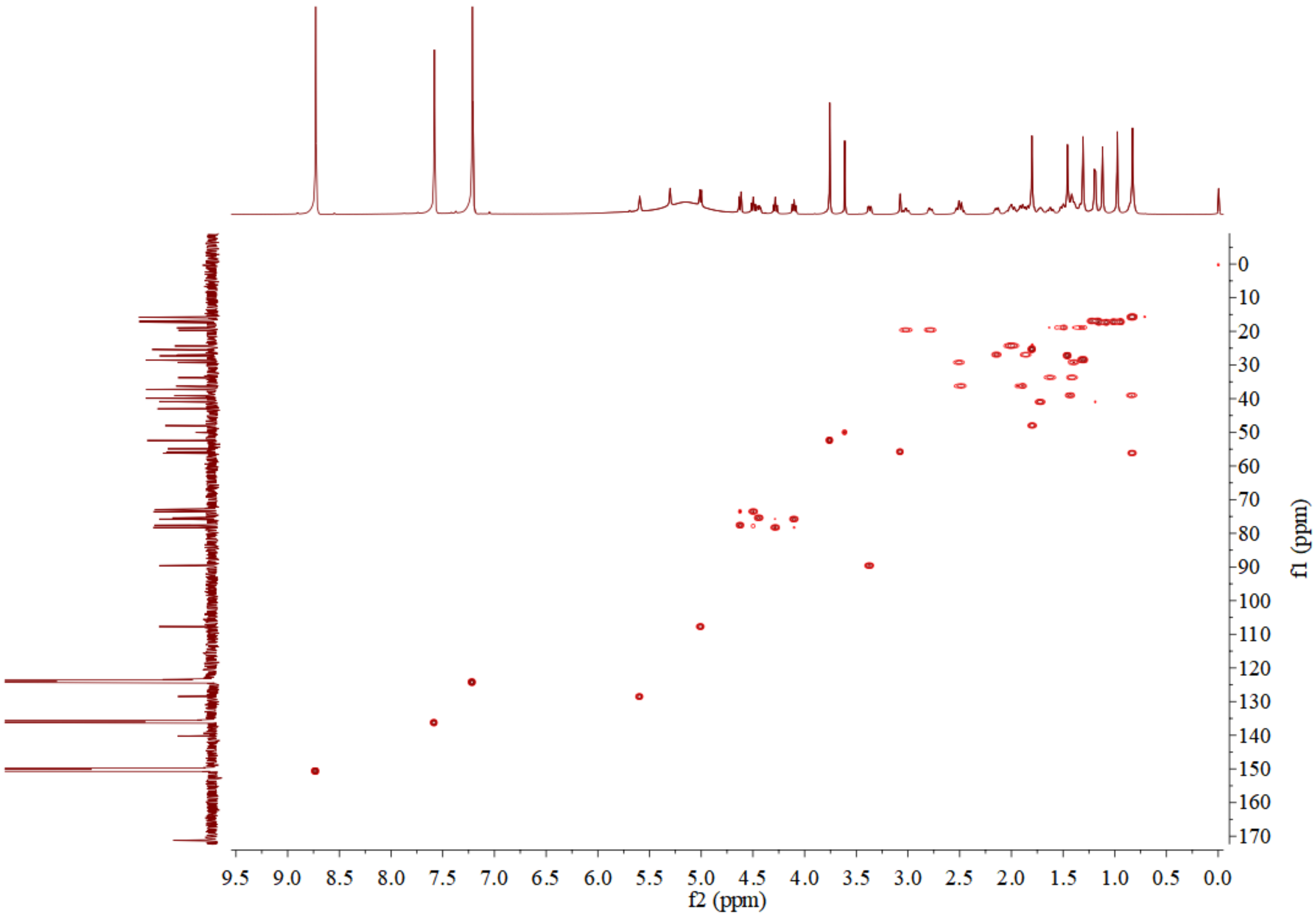

Figure S57. HMBC spectrum of **7** in pyridine-d_5 (500 MHz)

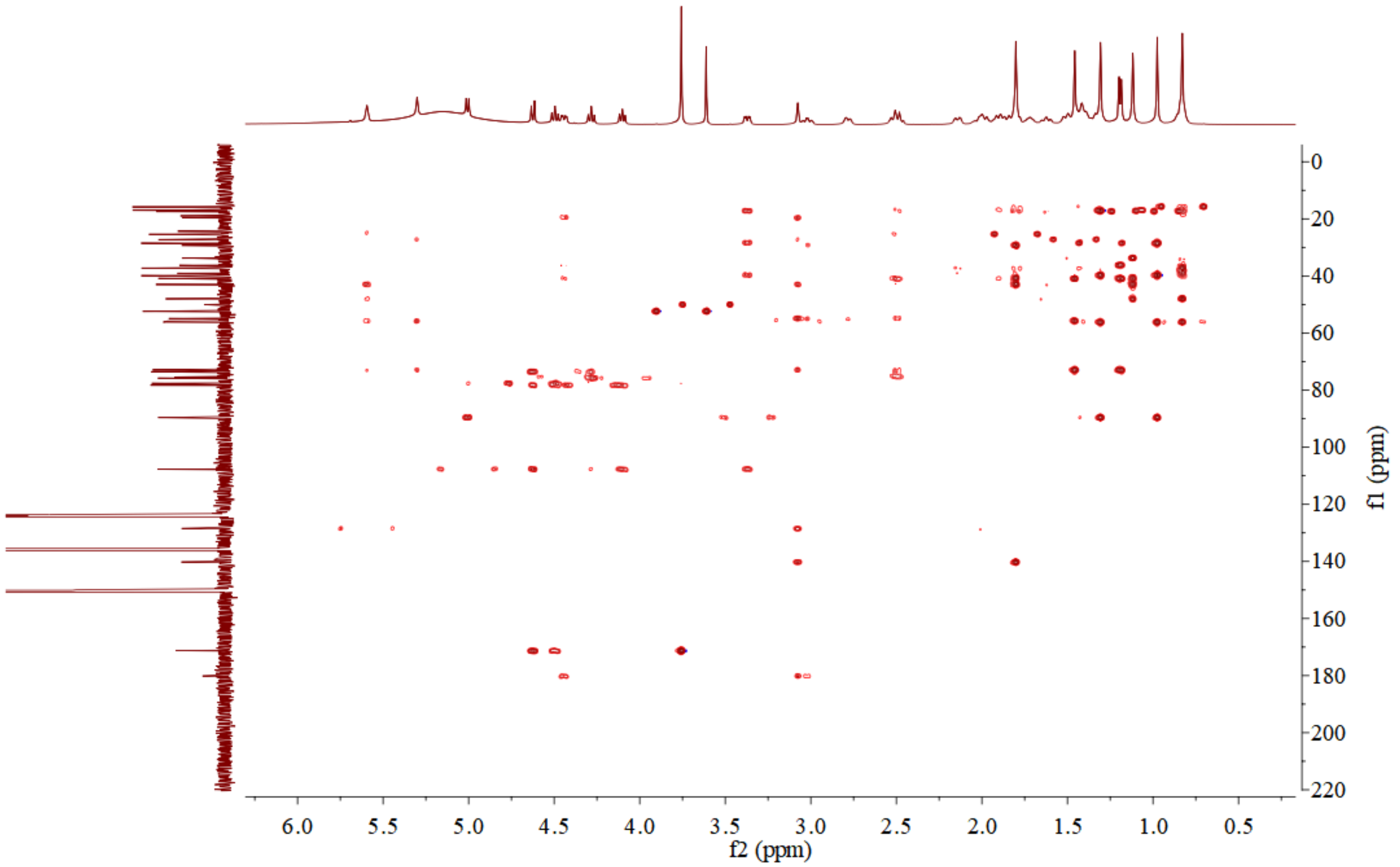

Figure S58. ^1H–^1H COSY spectrum of **7** in pyridine-d_5 (500 MHz)

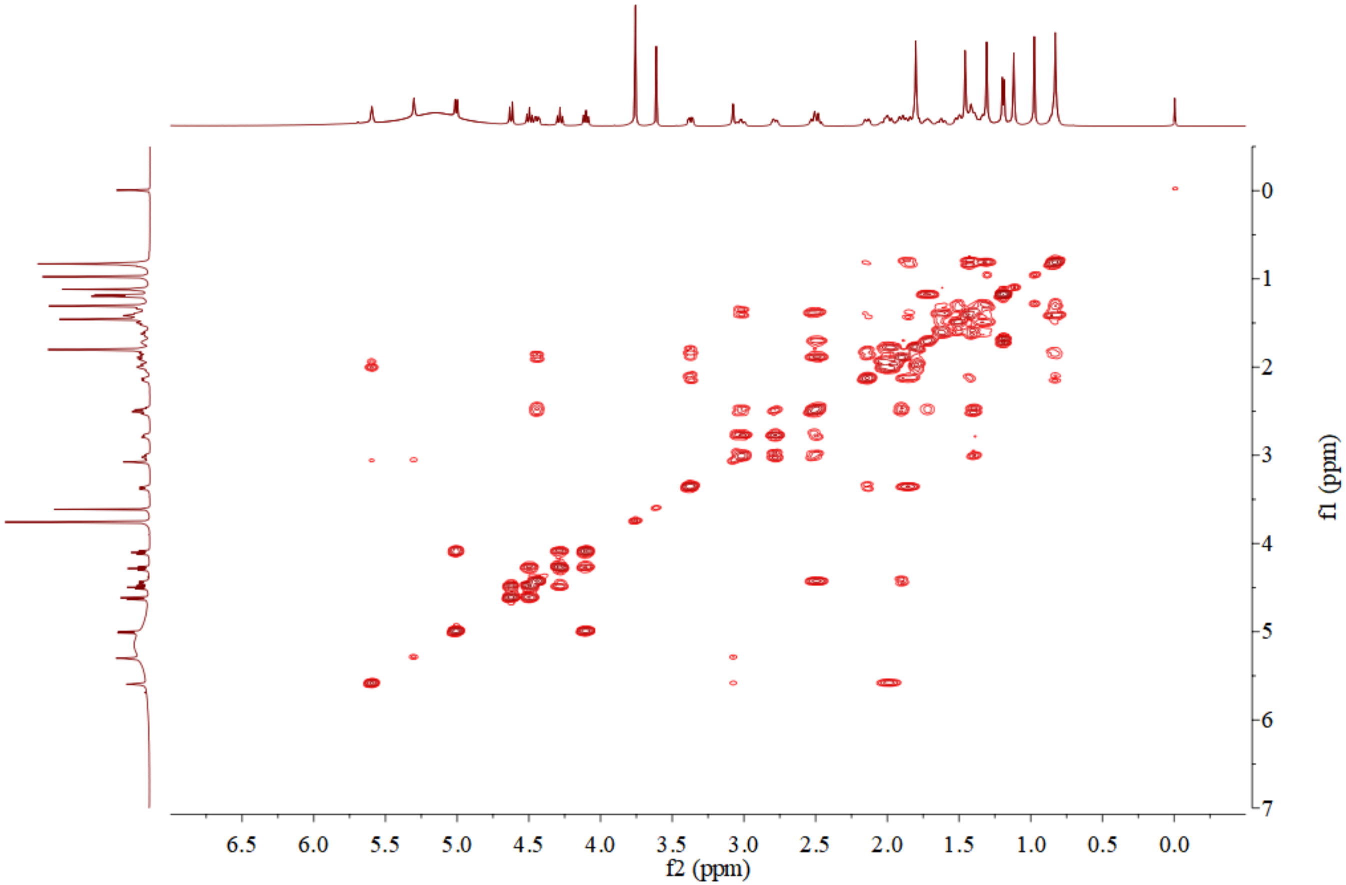

Figure S59. NOESY spectrum of **7** in pyridine-d_5 (500 MHz)

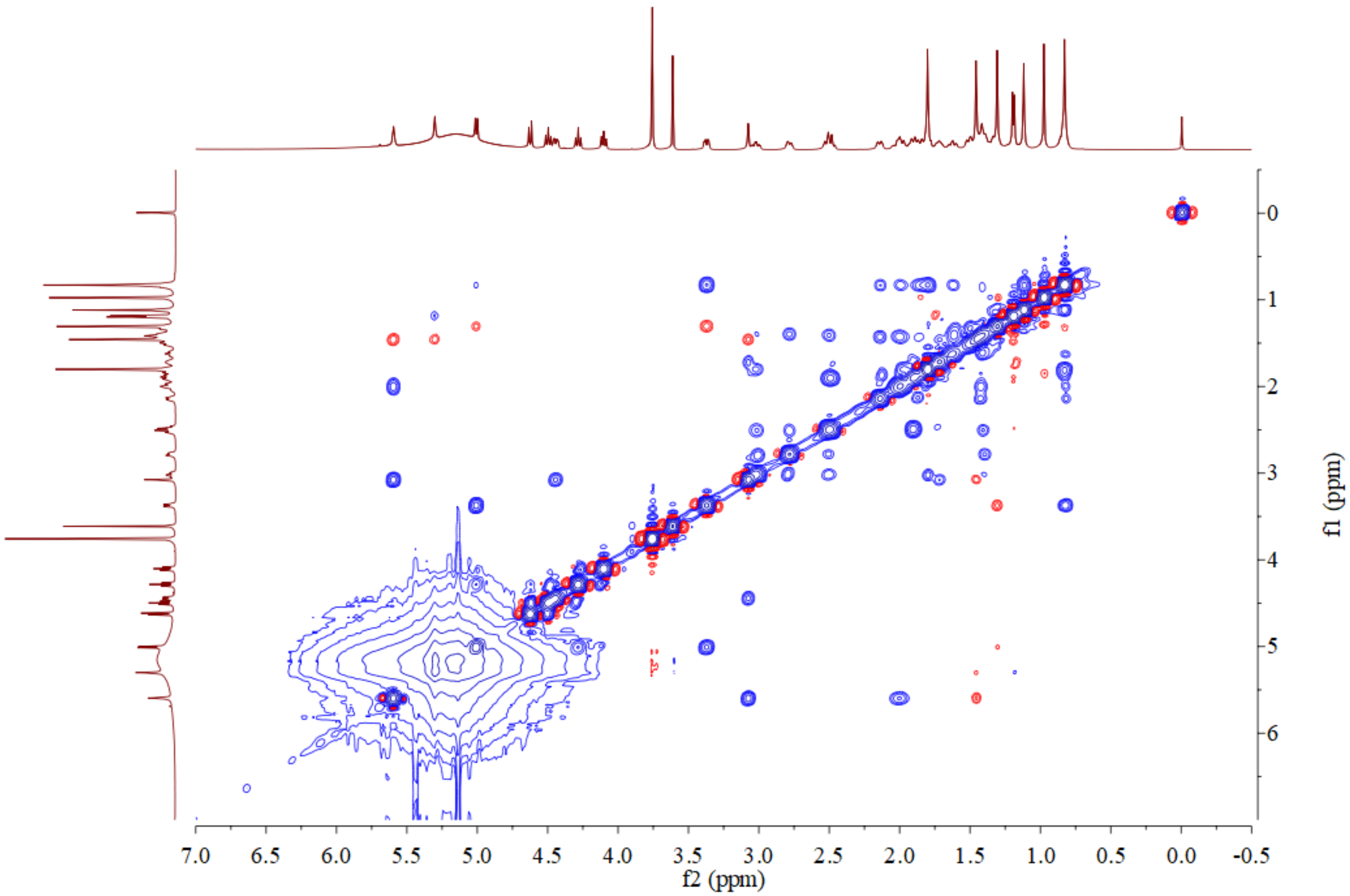

Figure S60. HRESIMS spectrum of **7**

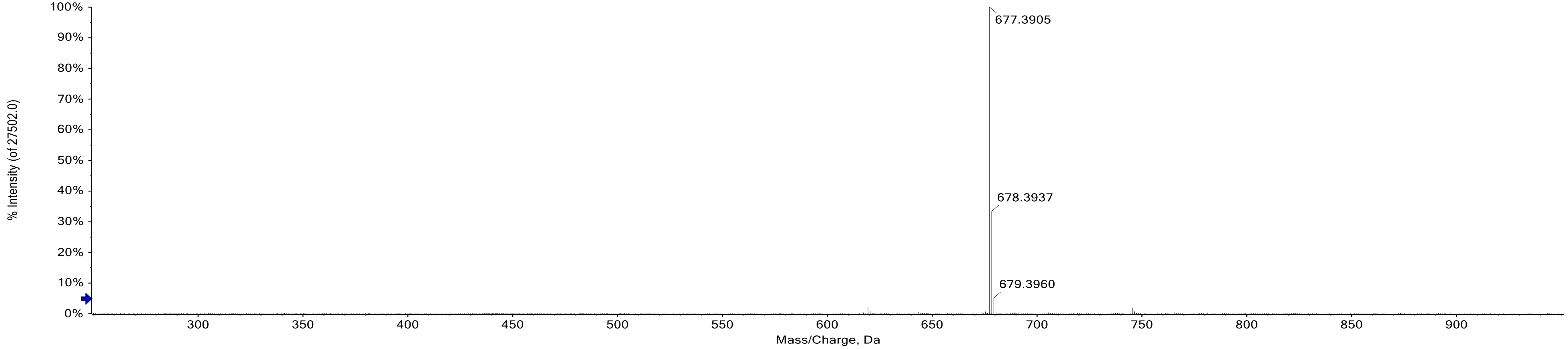

Figure S61. IR (KBr disc) spectrum of **7**

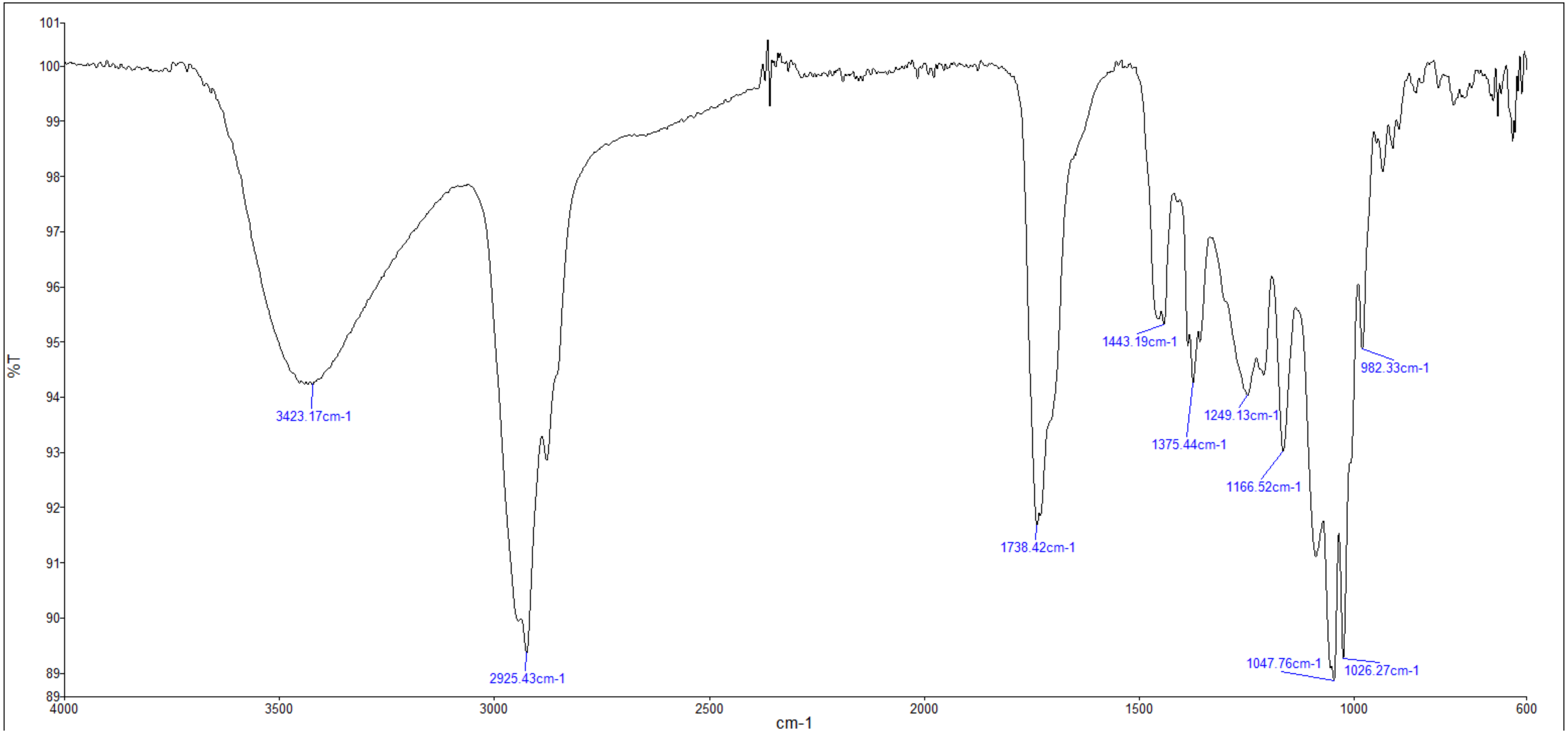

Figure S62. ¹H NMR spectrum of **8** in pyridine-d_5 (500 MHz)

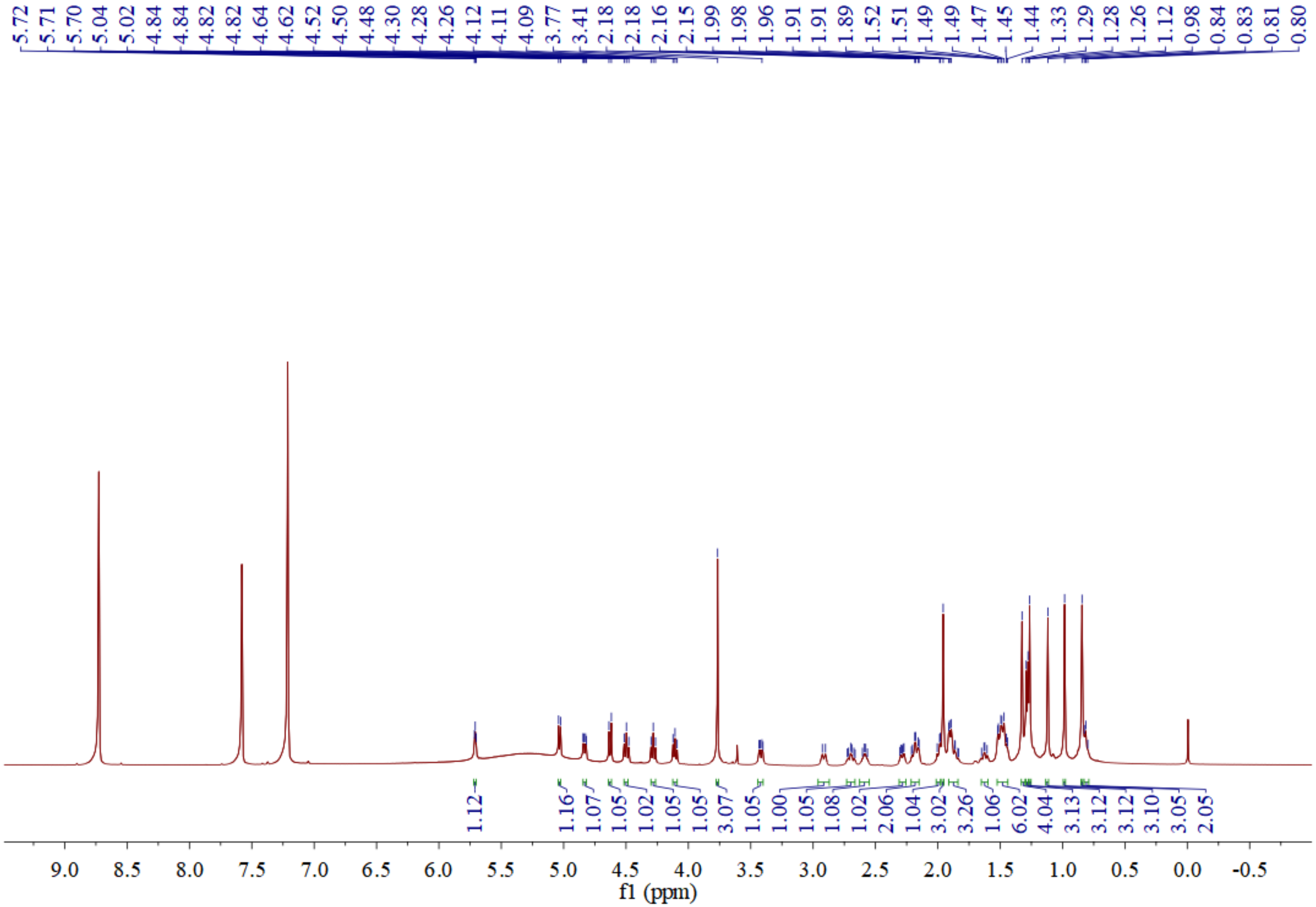

Figure S63. ^{13}C NMR and DEPT spectra of **8** in pyridine-d_5 (125 MHz)

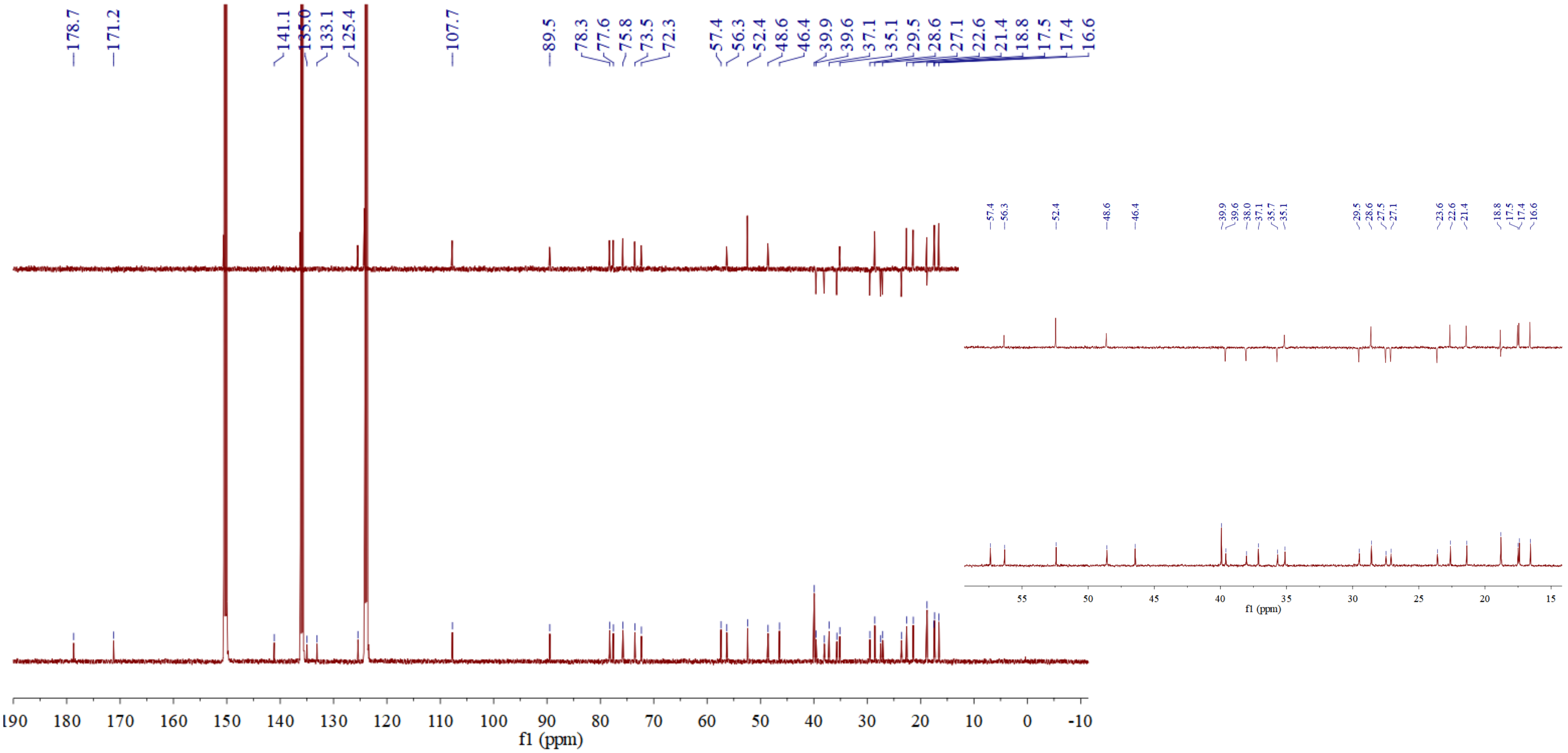

Figure S64. HSQC spectrum of **8** in pyridine-d_5 (500 MHz)

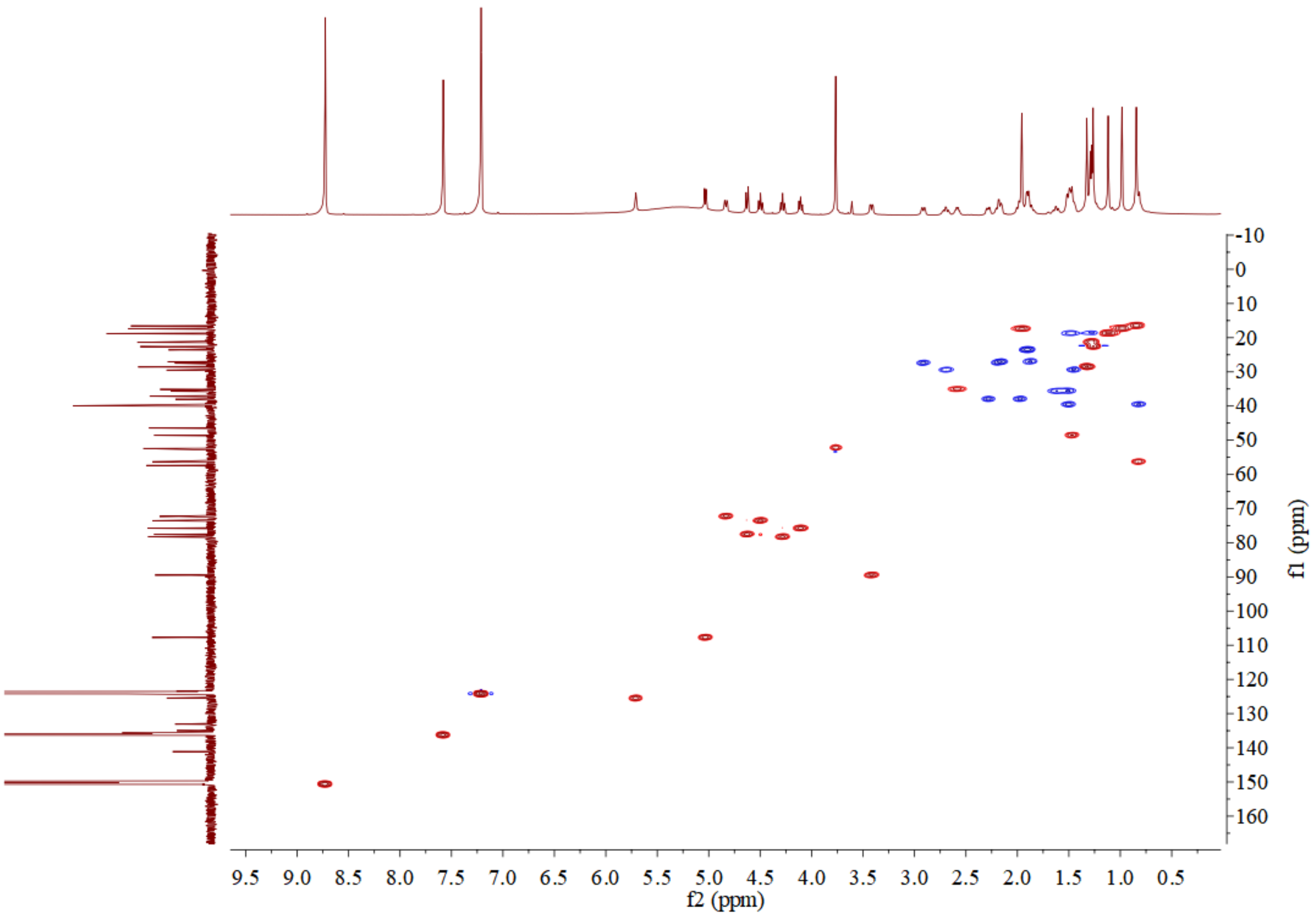

Figure S65. HMBC spectrum of **8** in pyridine-d_5 (500 MHz)

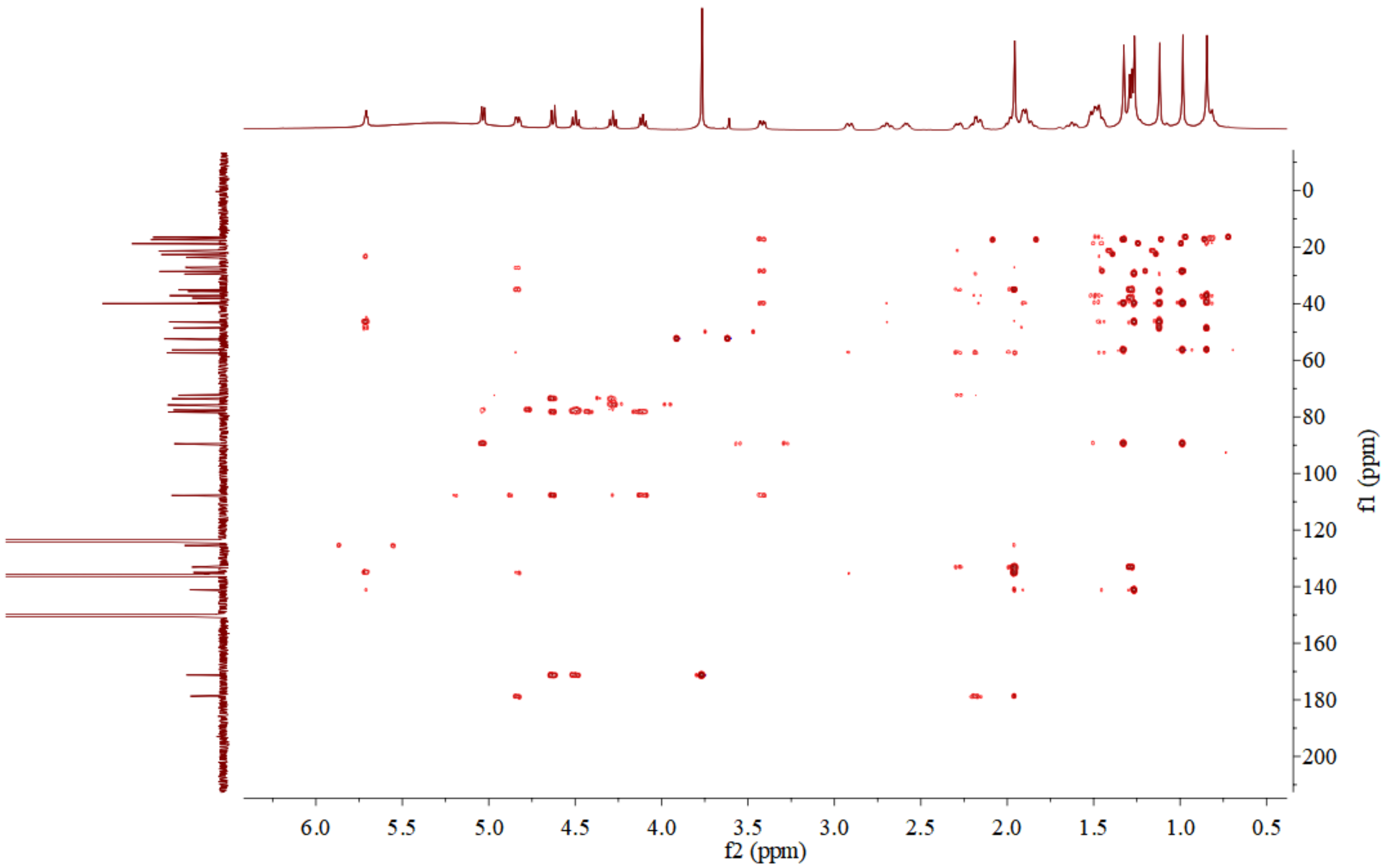

Figure S66. ^{1}H–^{1}H COSY spectrum of **8** in pyridine-d_5 (500 MHz)

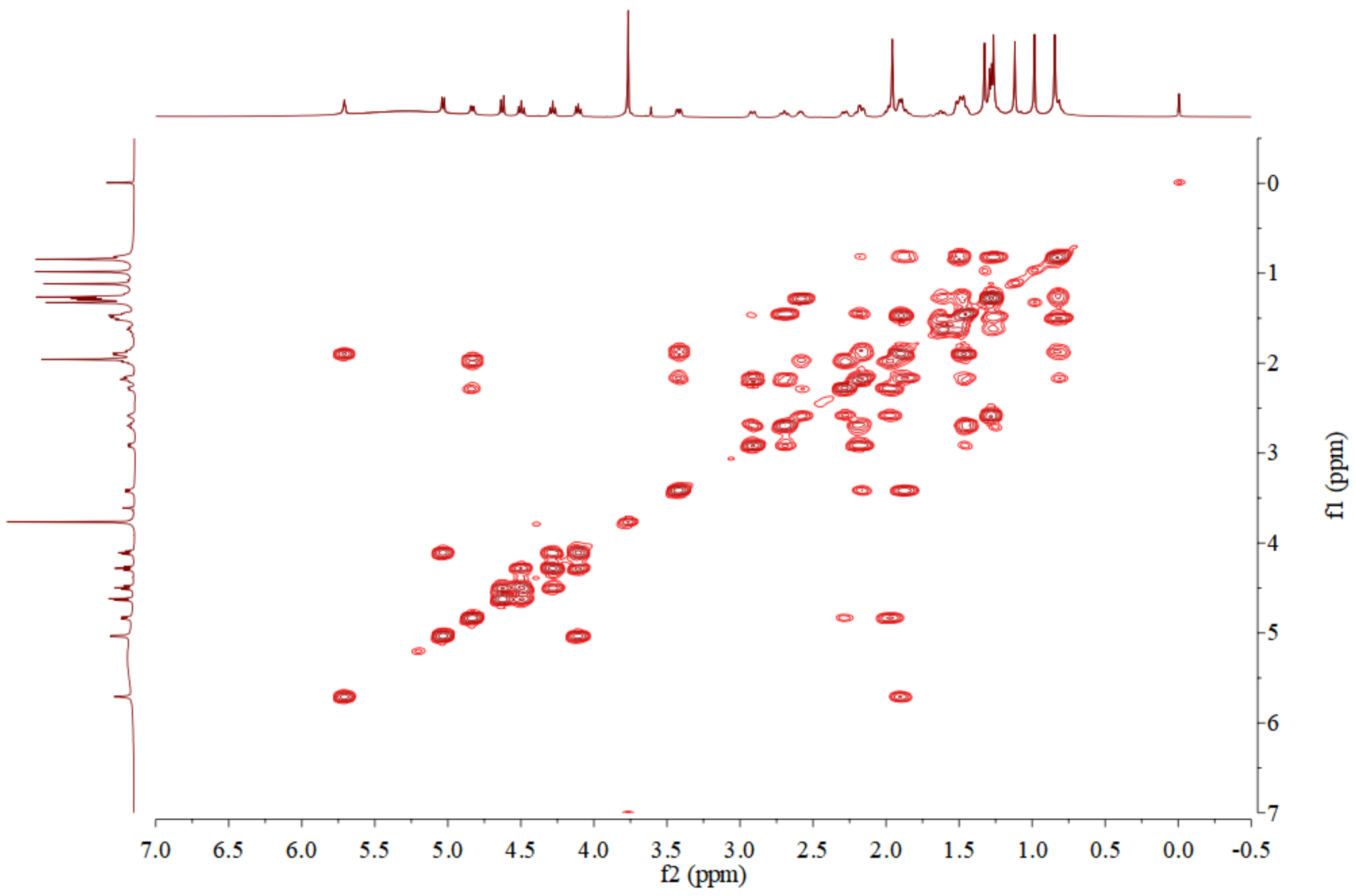

Figure S67. NOESY spectrum of **8** in pyridine-d_5 (500 MHz)

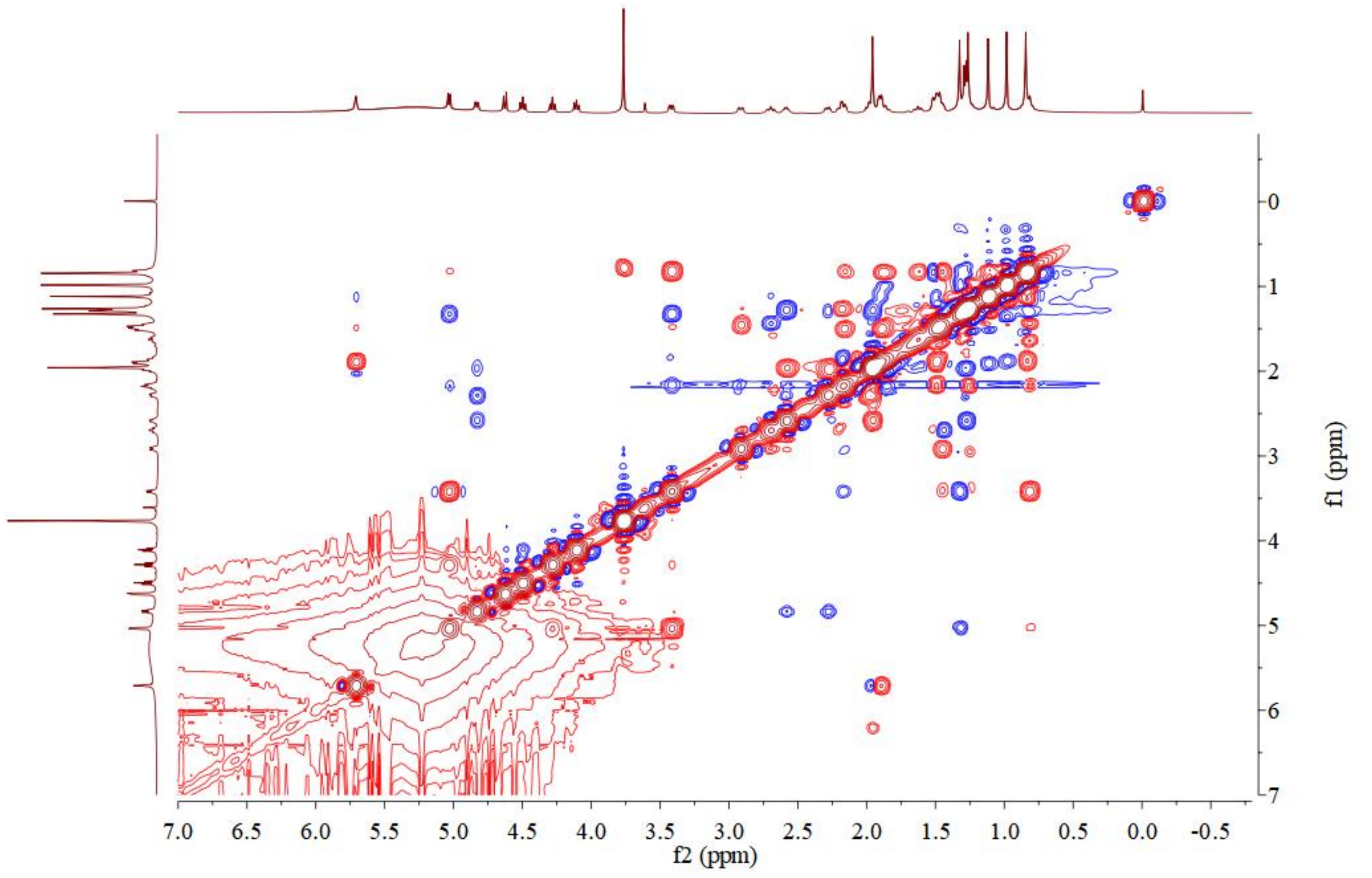

Figure S68. HRESIMS spectrum of **8**

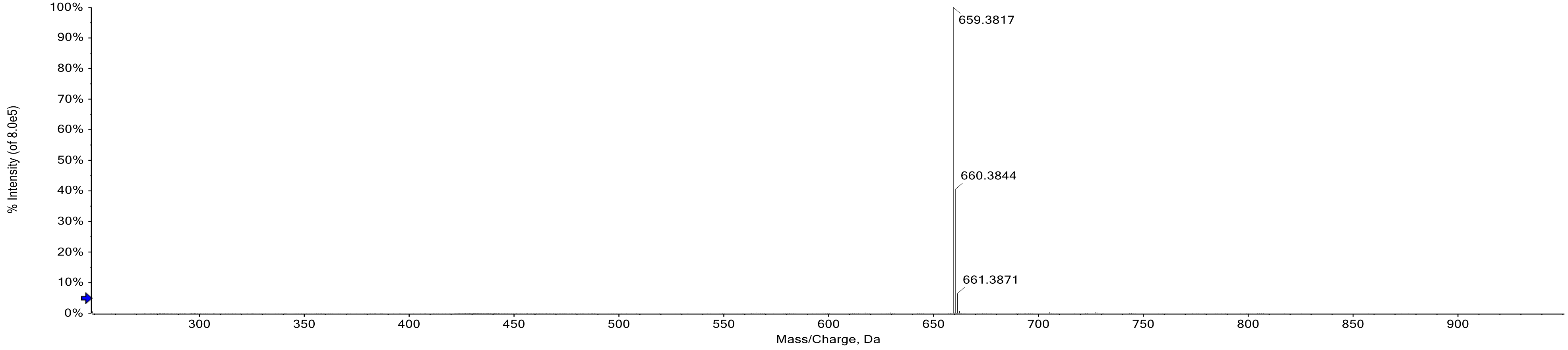

Figure S69. IR (KBr disc) spectrum of **8**

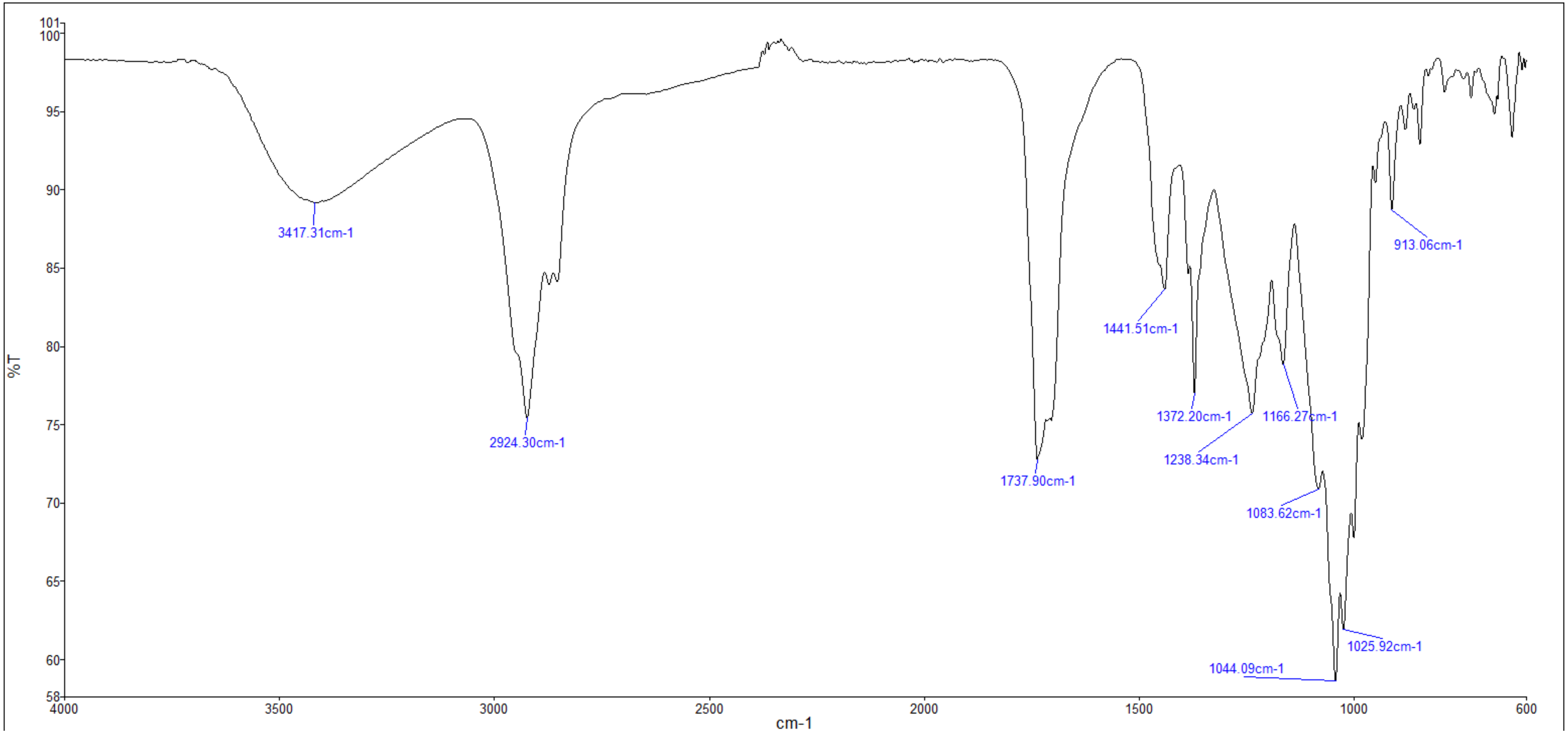

Figure S70. ^{1}H NMR spectrum of **9** in CD_3OD (500 MHz)

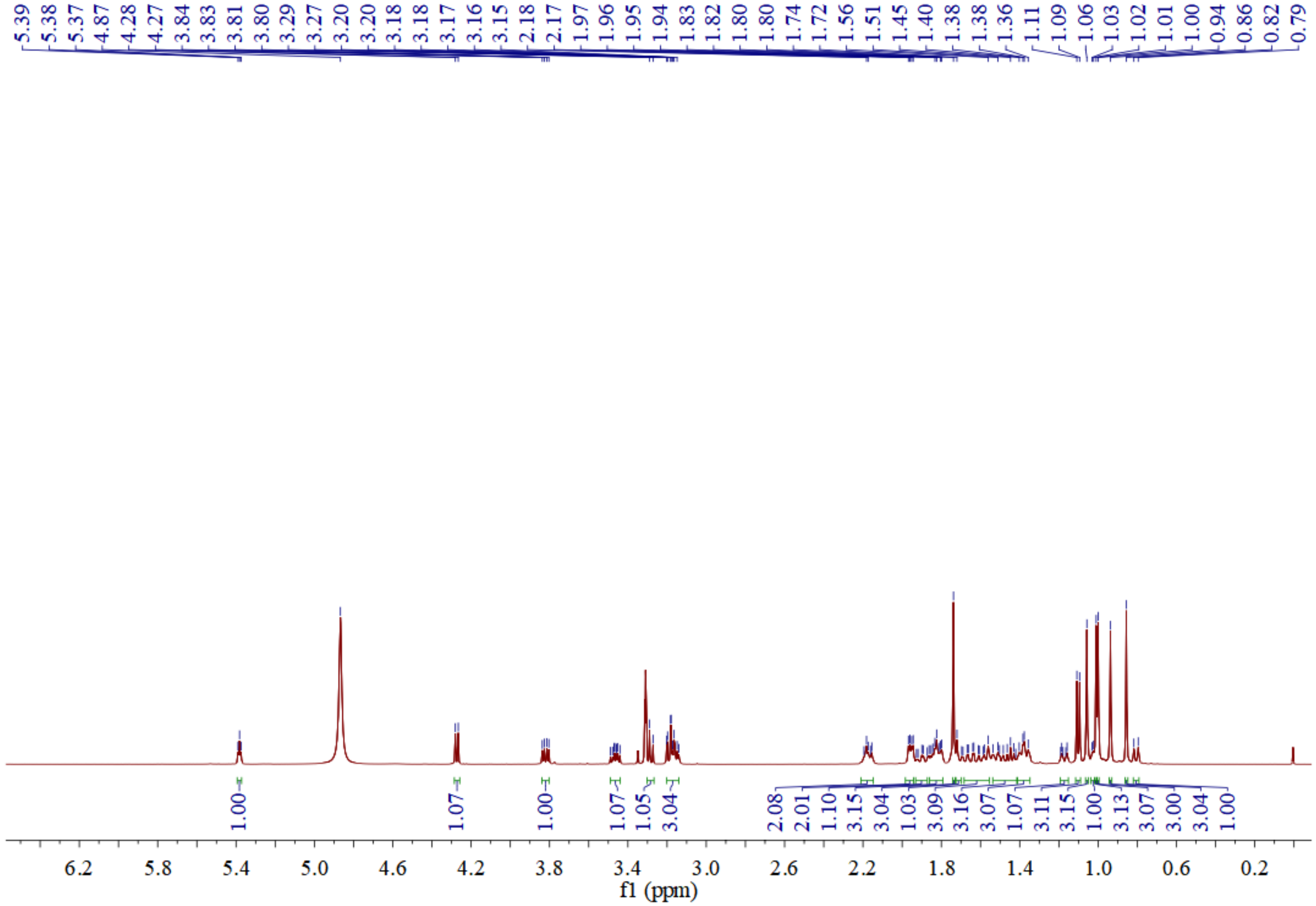

Figure S71. ^{13}C NMR and DEPT spectra of **9** in CD_3OD (125 MHz)

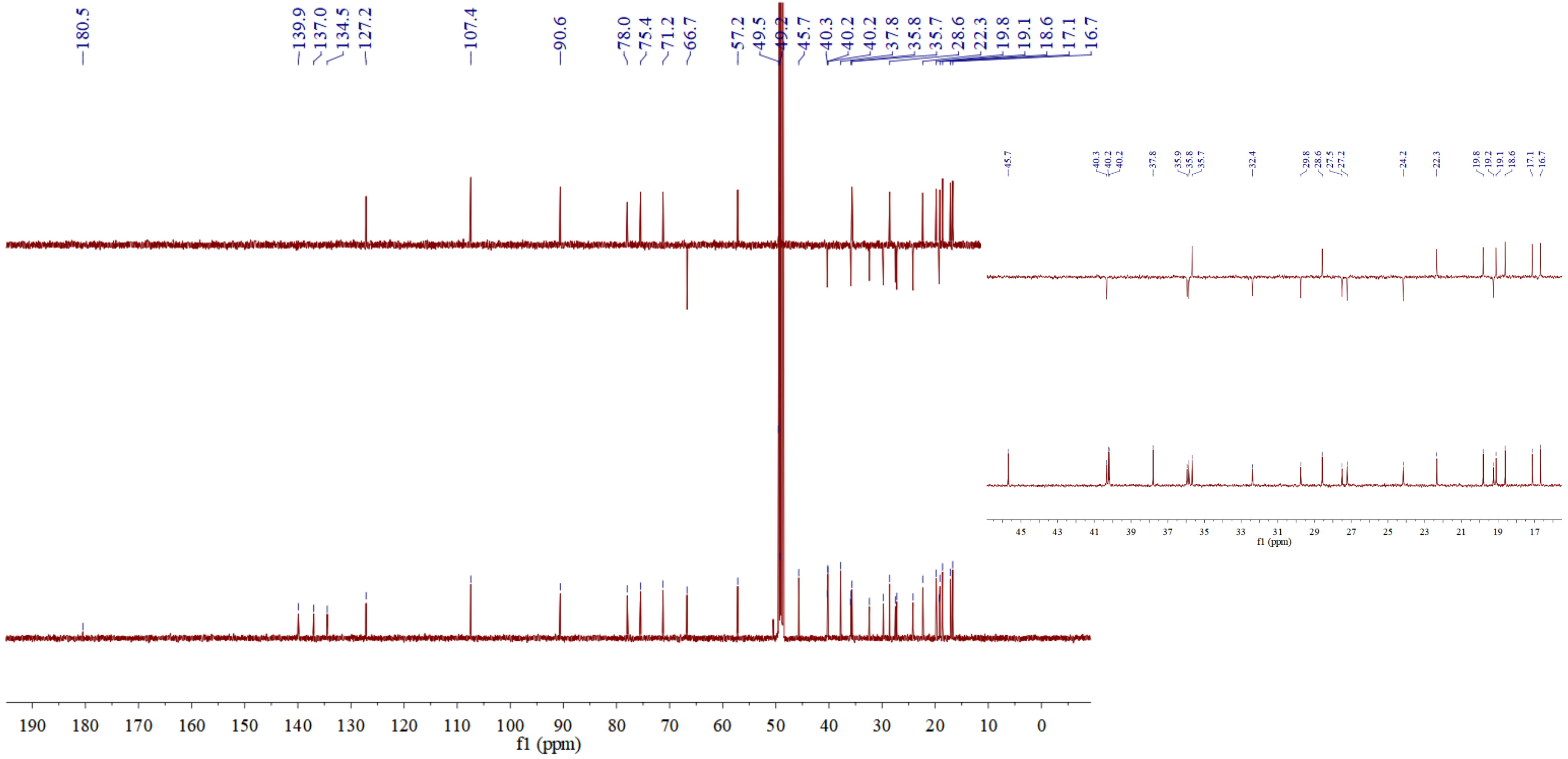

Figure S72. HSQC spectrum of **9** in CD_3OD (500 MHz)

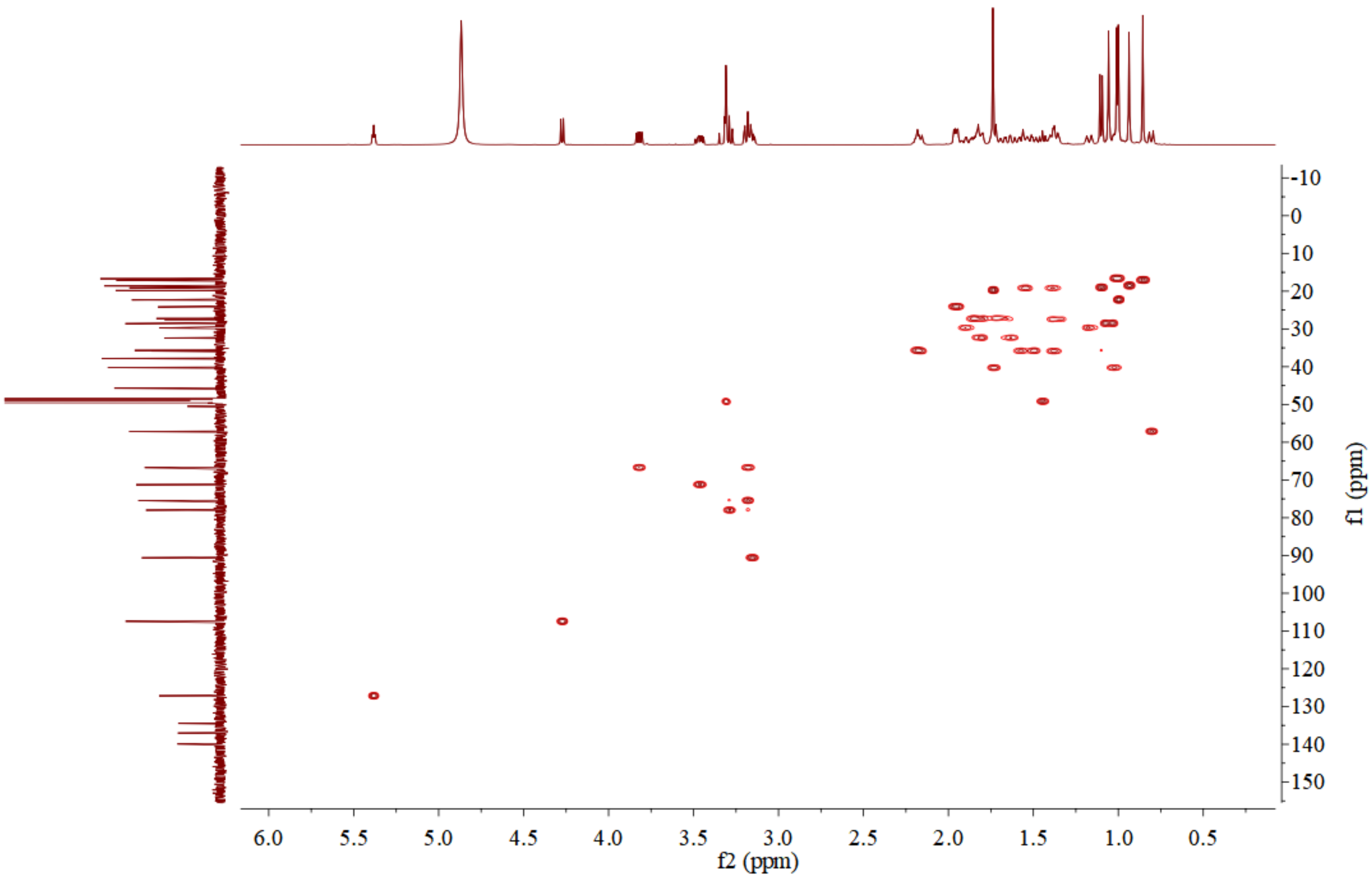

Figure S73. HMBC spectrum of **9** in CD_3OD (500 MHz)

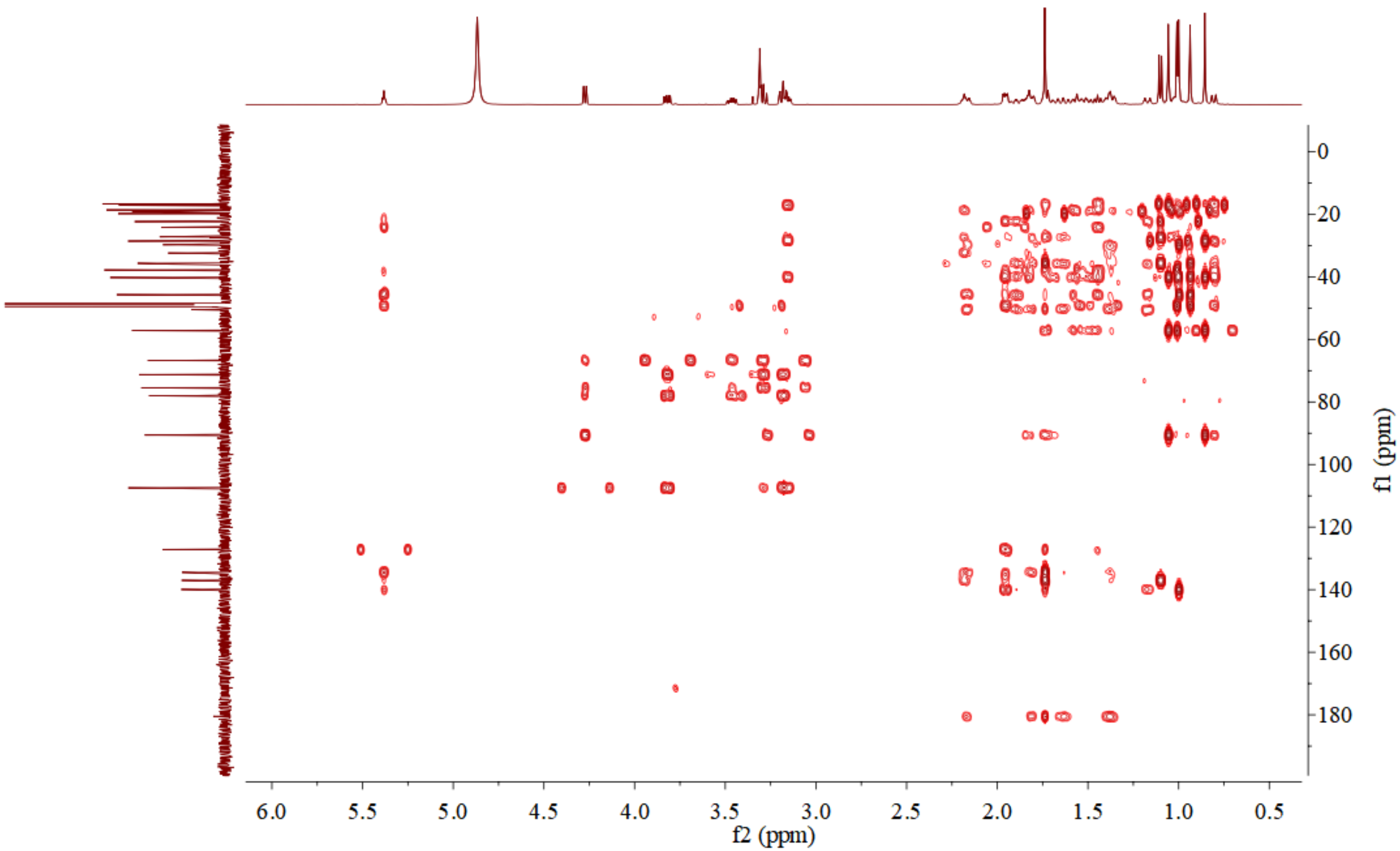

Figure S74. ^{1}H–^{1}H COSY spectrum of **9** in CD_3OD (500 MHz)

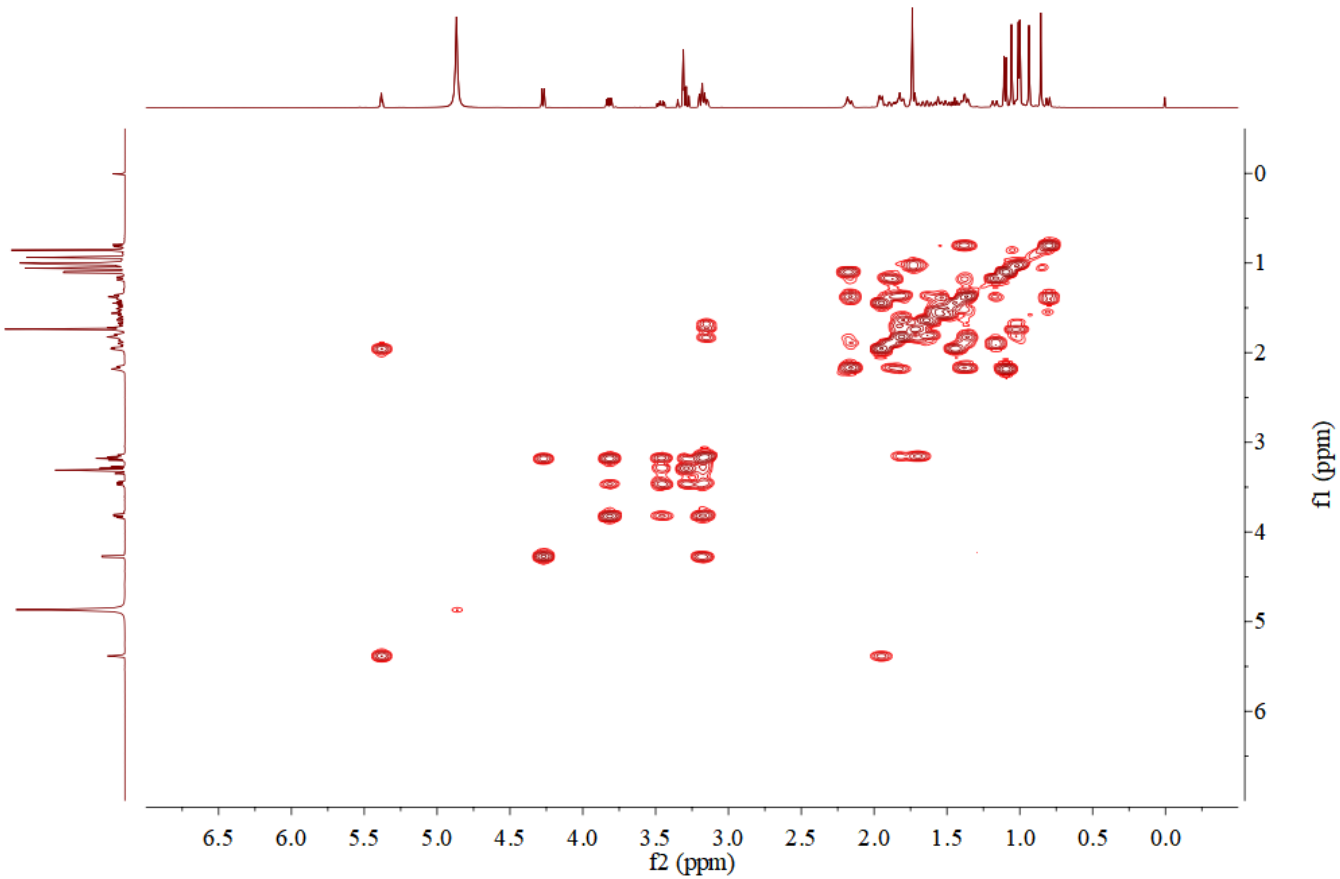

Figure S75. NOESY spectrum of **9** in CD_3OD (500 MHz)

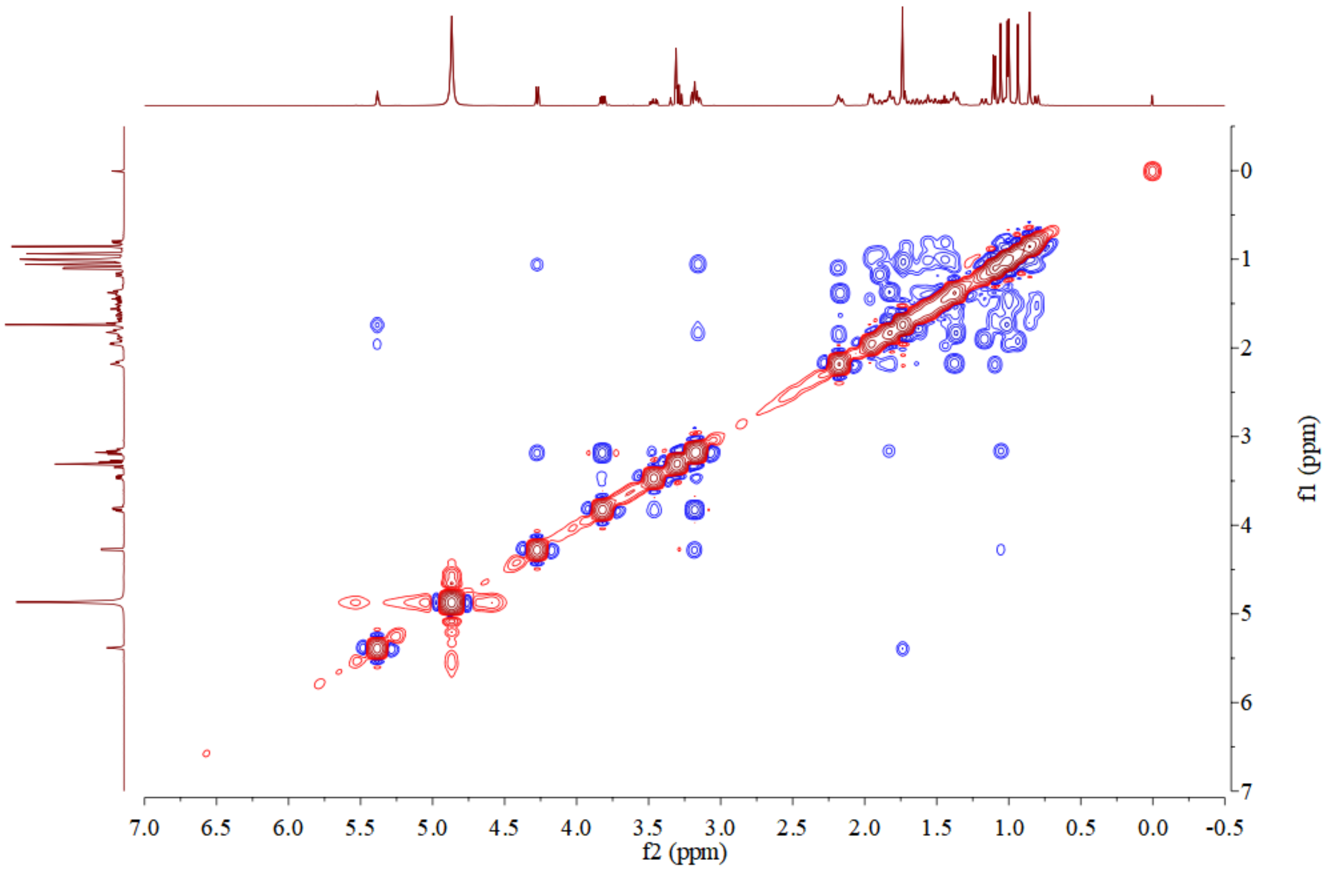

Figure S76. HRESIMS spectrum of **9**

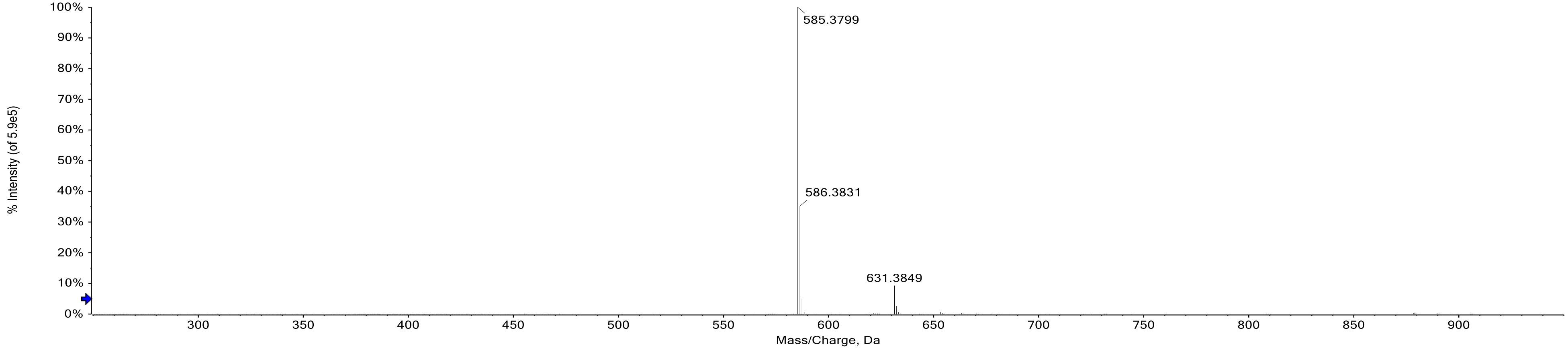

Figure S77. IR (KBr disc) spectrum of **9**

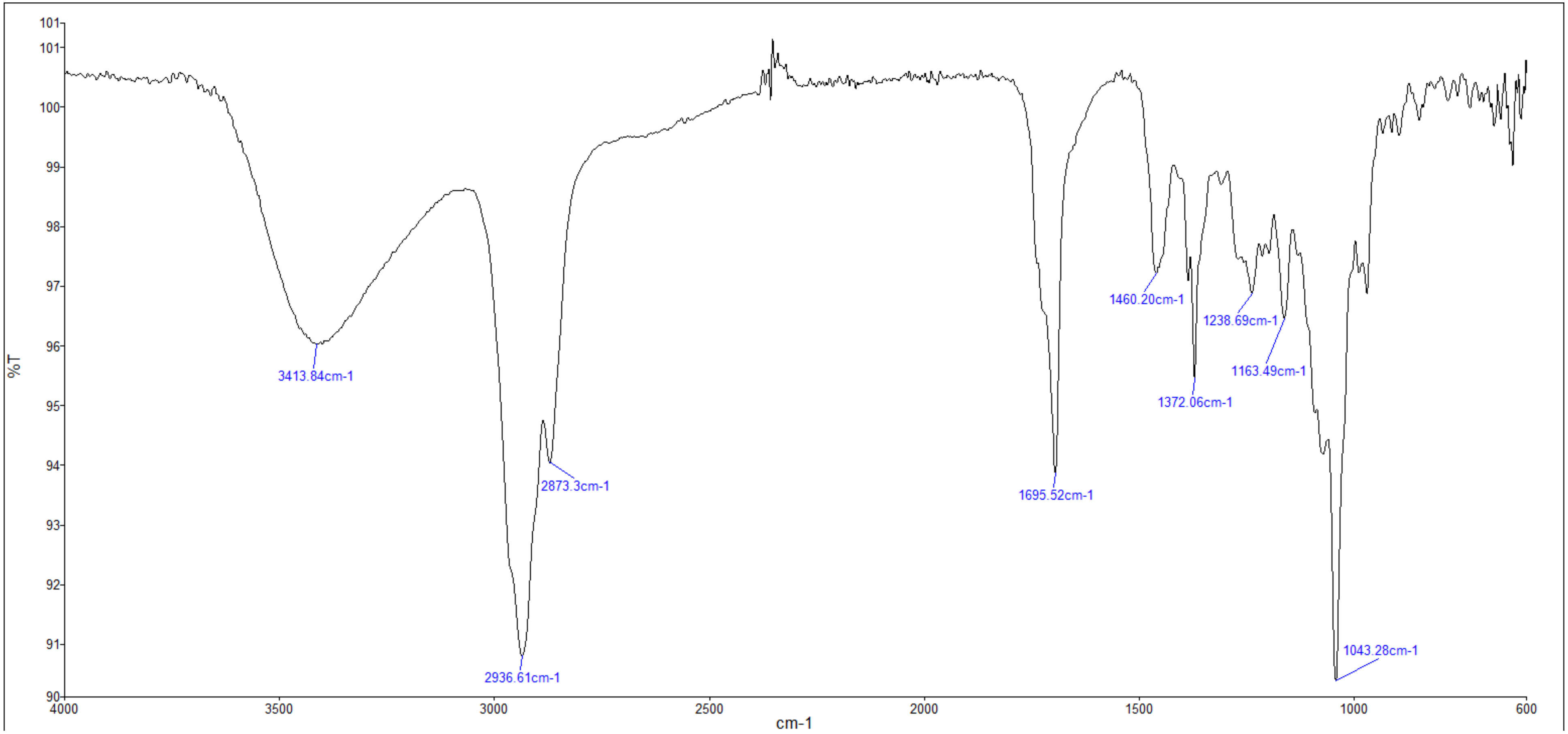

Figure S78. ^{1}H NMR spectrum of **10** in pyridine-d_5 (500 MHz)

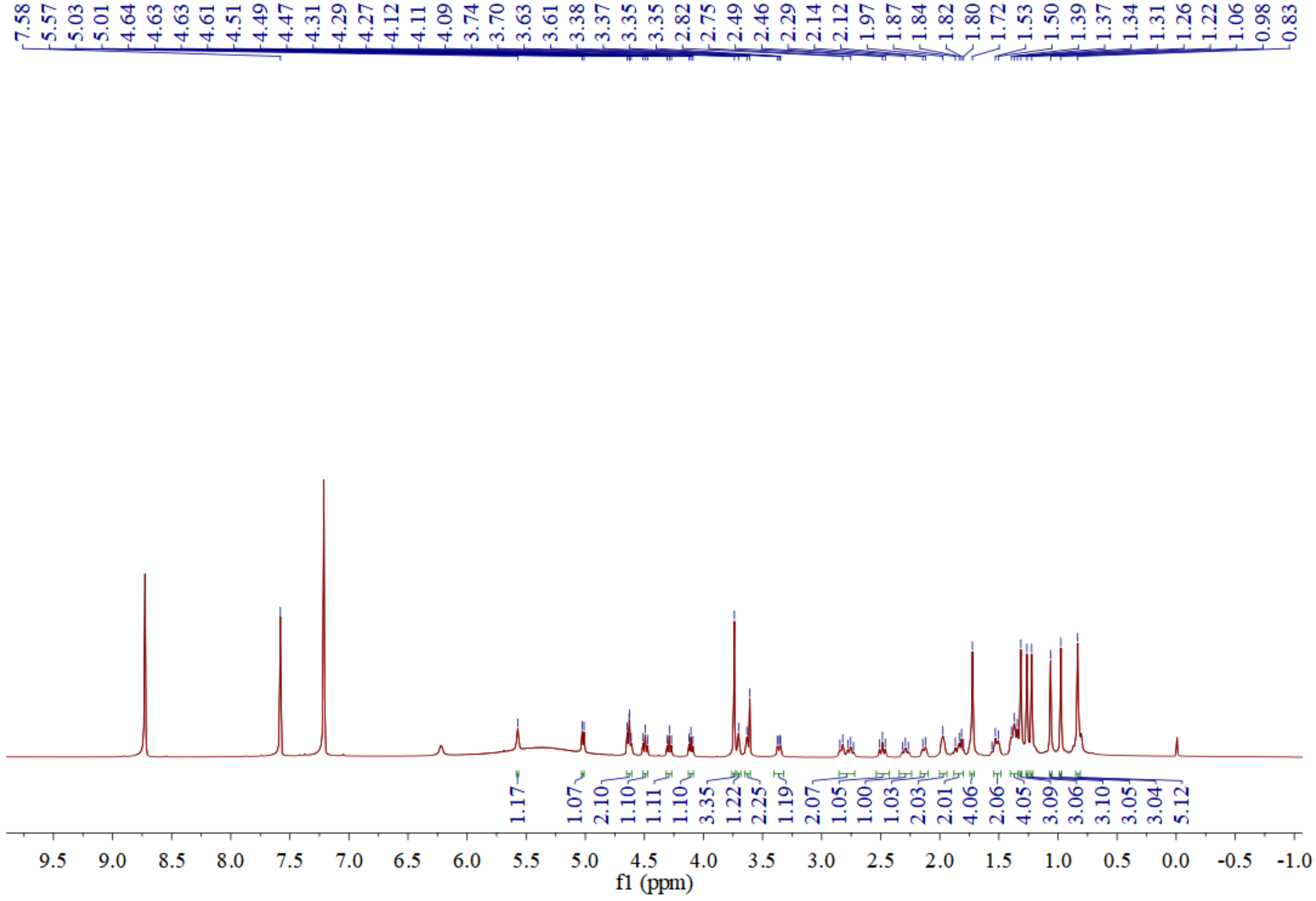

Figure S79. ^{13}C NMR and DEPT spectra of **10** in pyridine-d_5 (125 MHz)

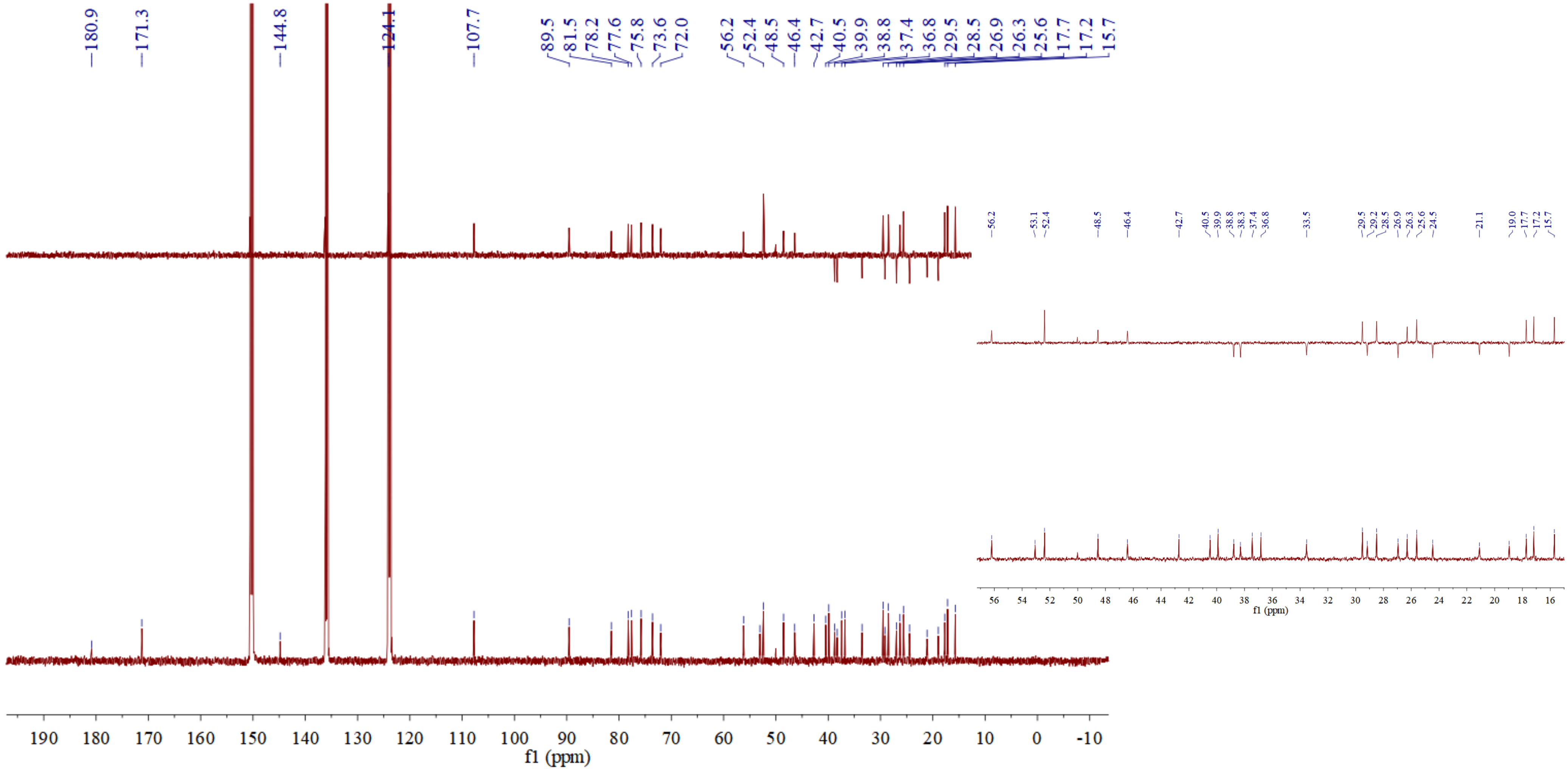

Figure S80. HSQC spectrum of **10** in pyridine-d_5 (500 MHz)

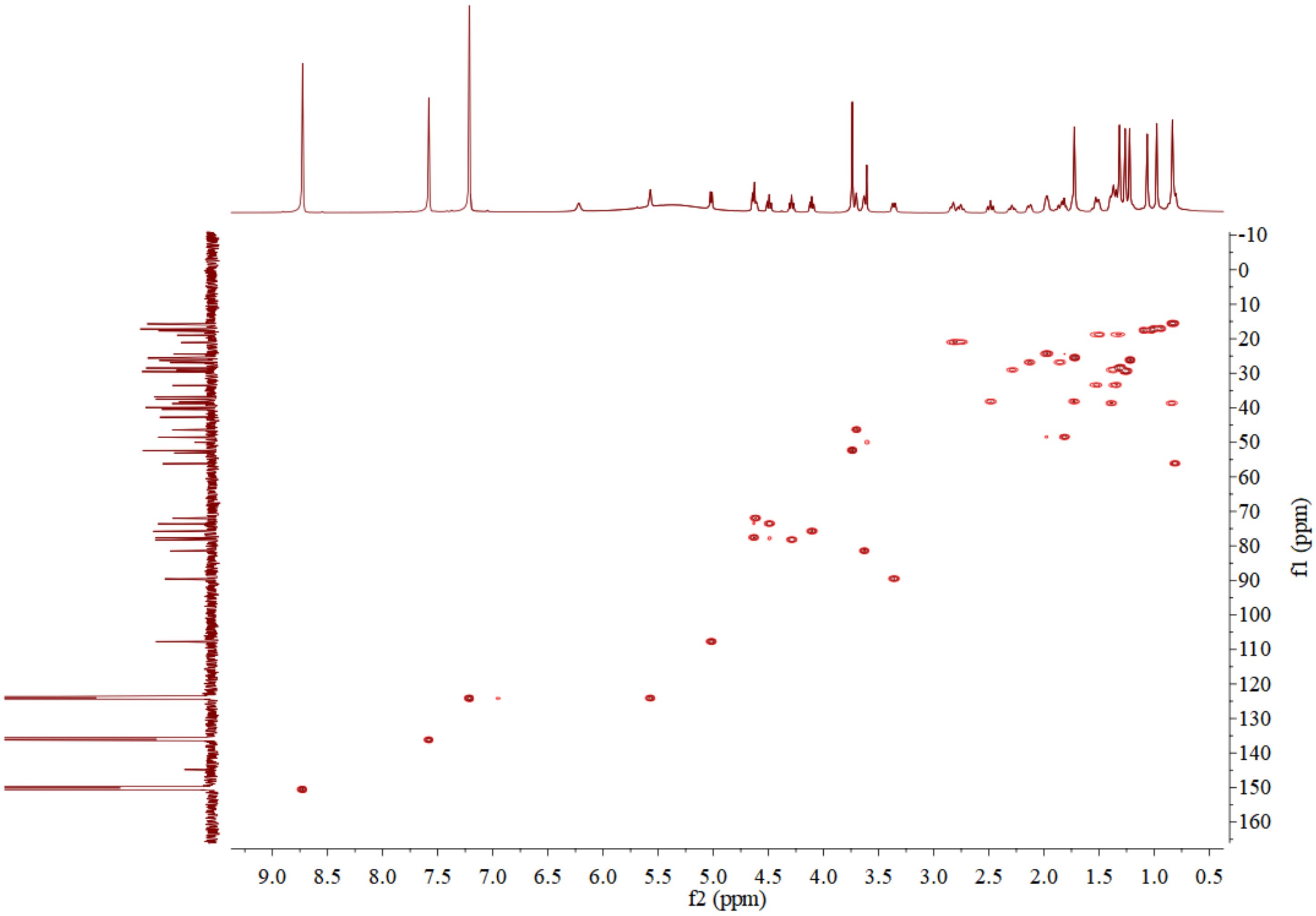

Figure S81. HMBC spectrum of **10** in pyridine-d_5 (500 MHz)

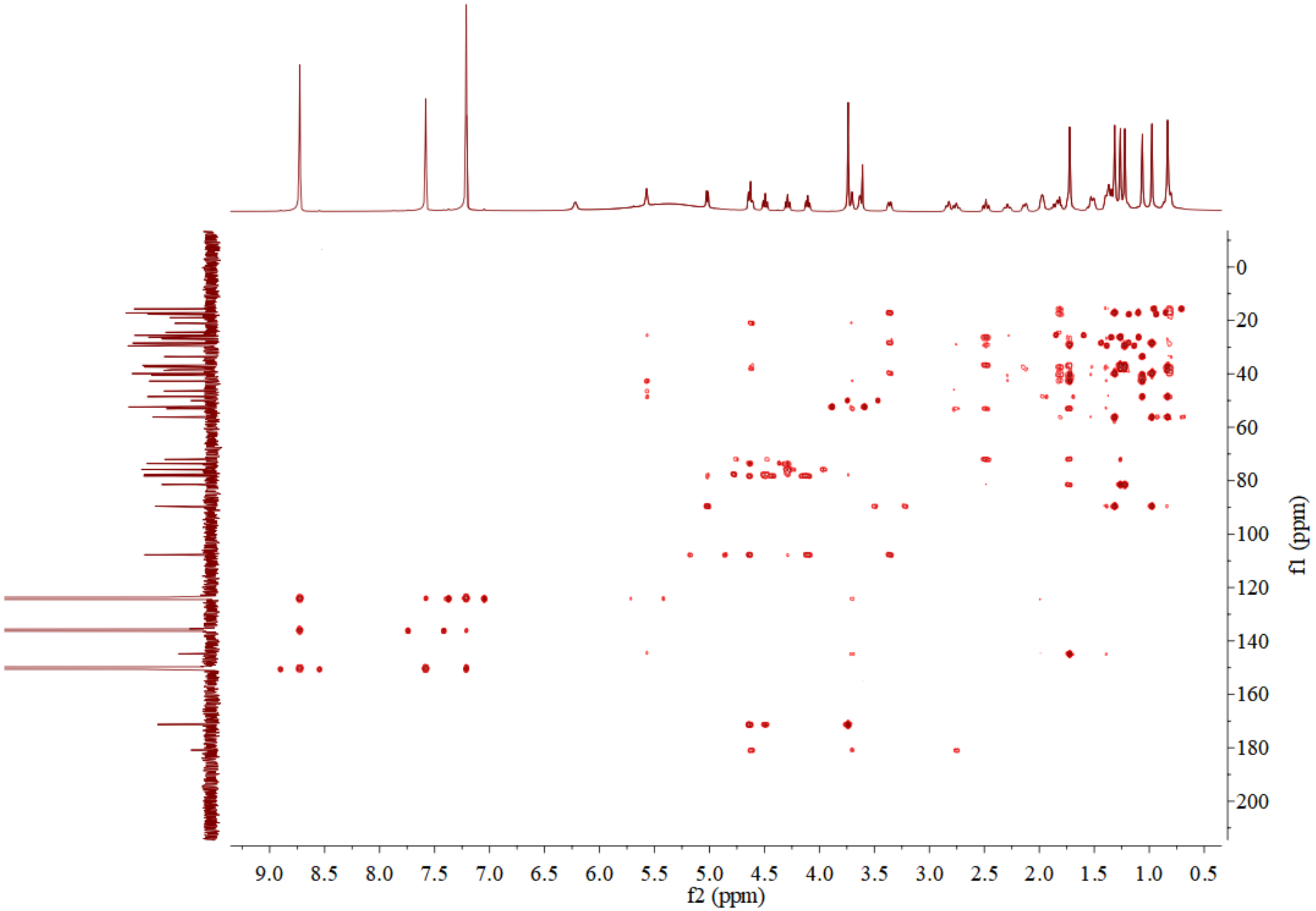

Figure S82. ^1H–^1H COSY spectrum of **10** in pyridine-d_5 (500 MHz)

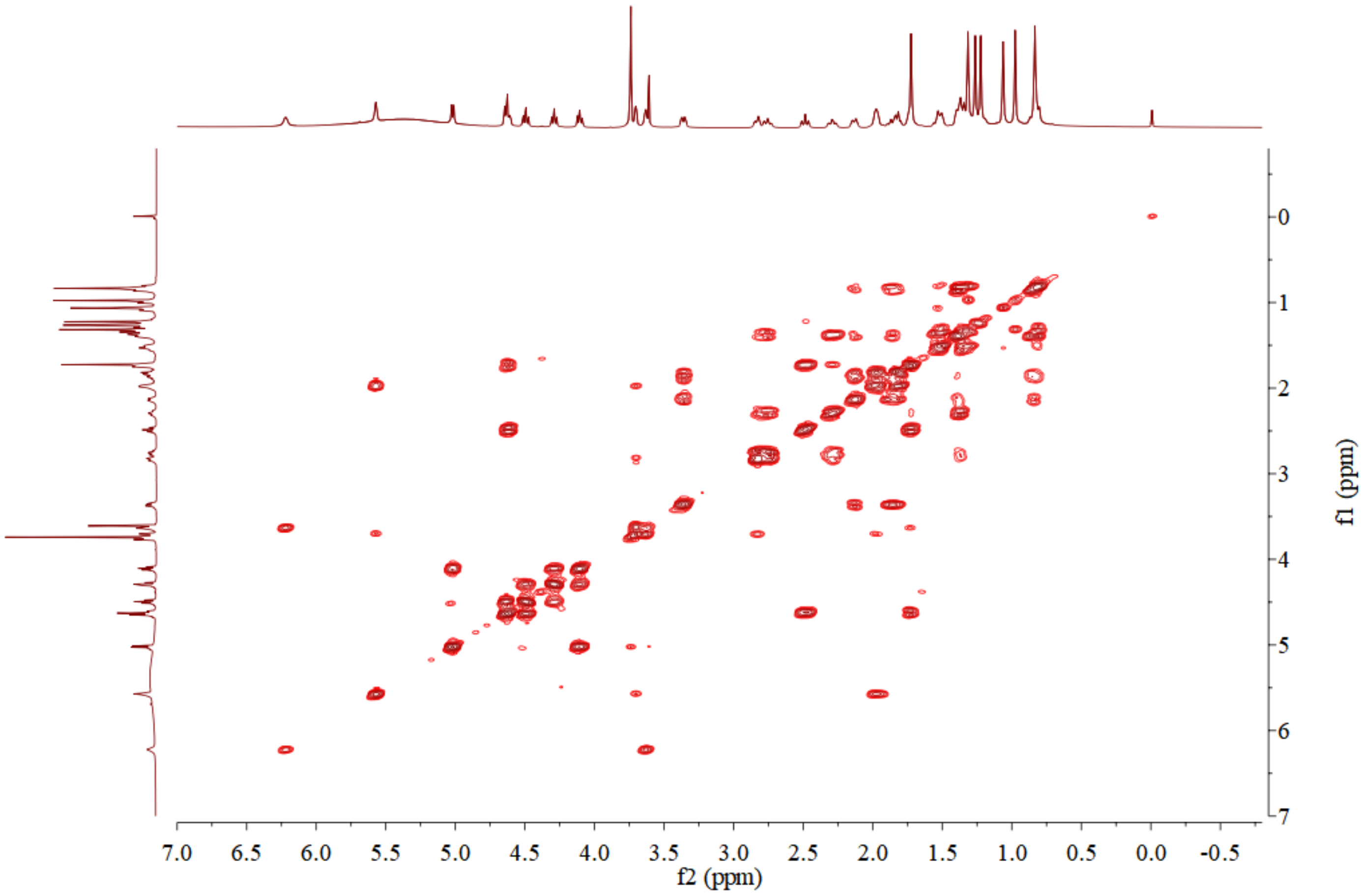

Figure S83. NOESY spectrum of **10** in pyridine-d_5 (500 MHz)

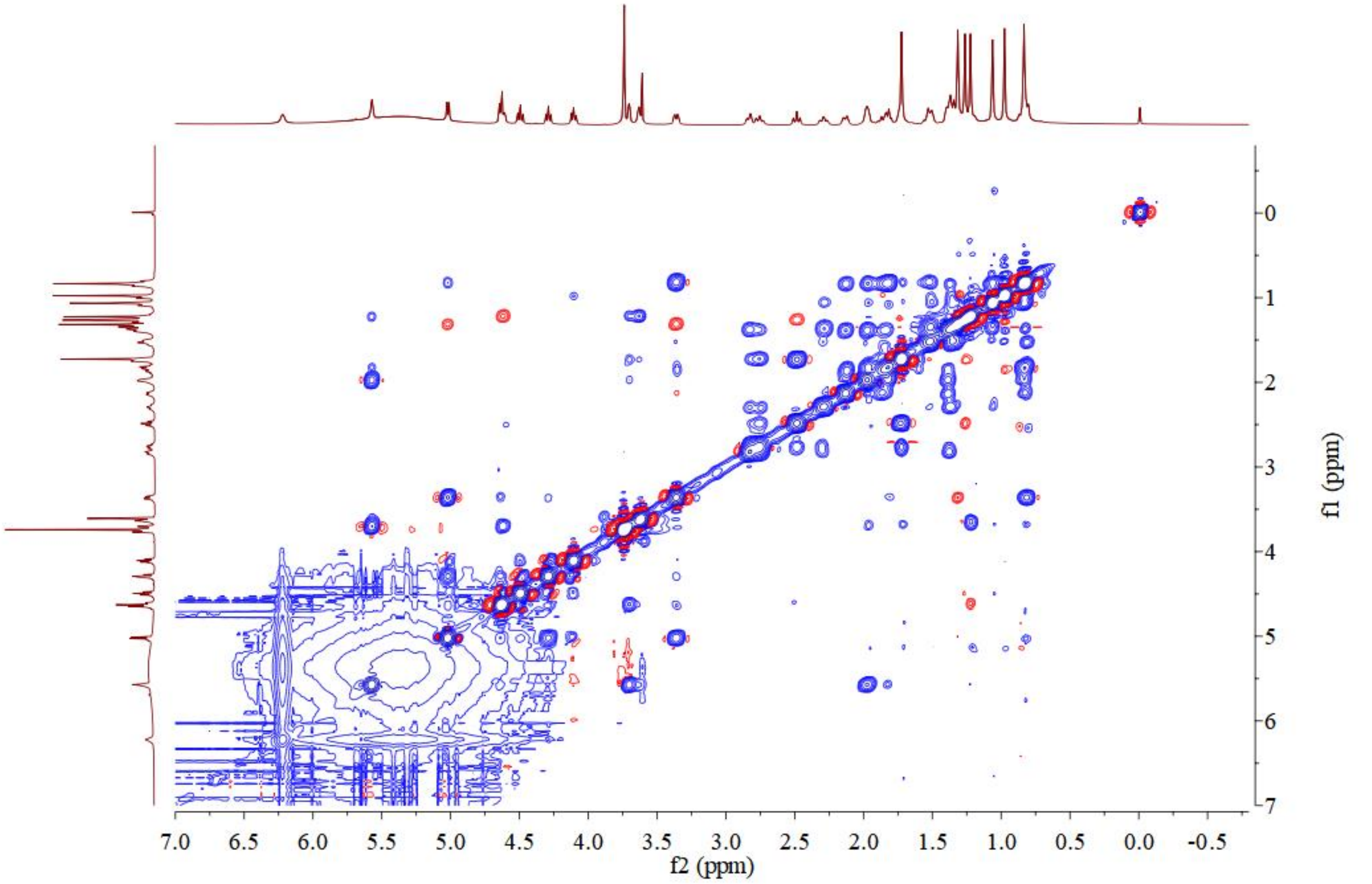

Figure S84. HRESIMS spectrum of **10**

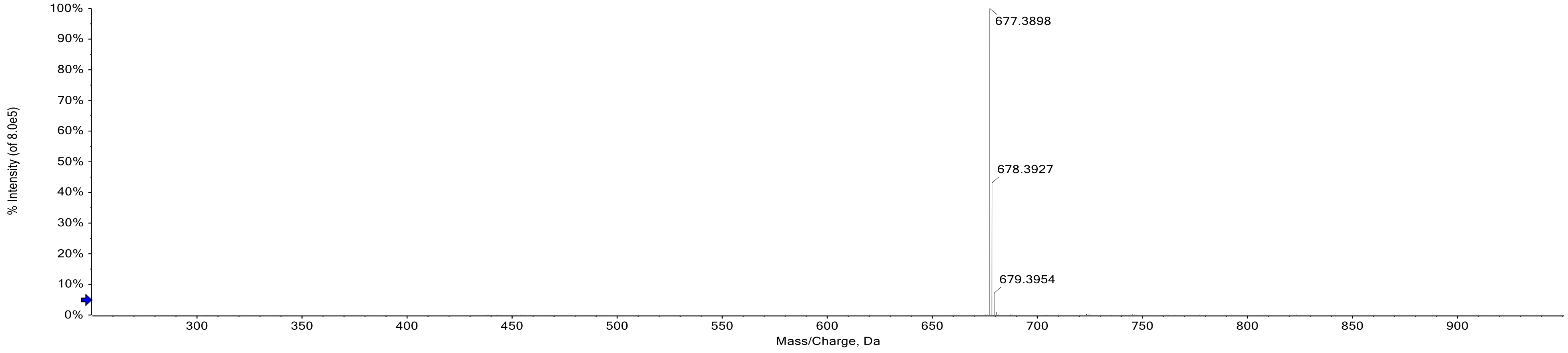

Figure S85. IR (KBr disc) spectrum of **10**

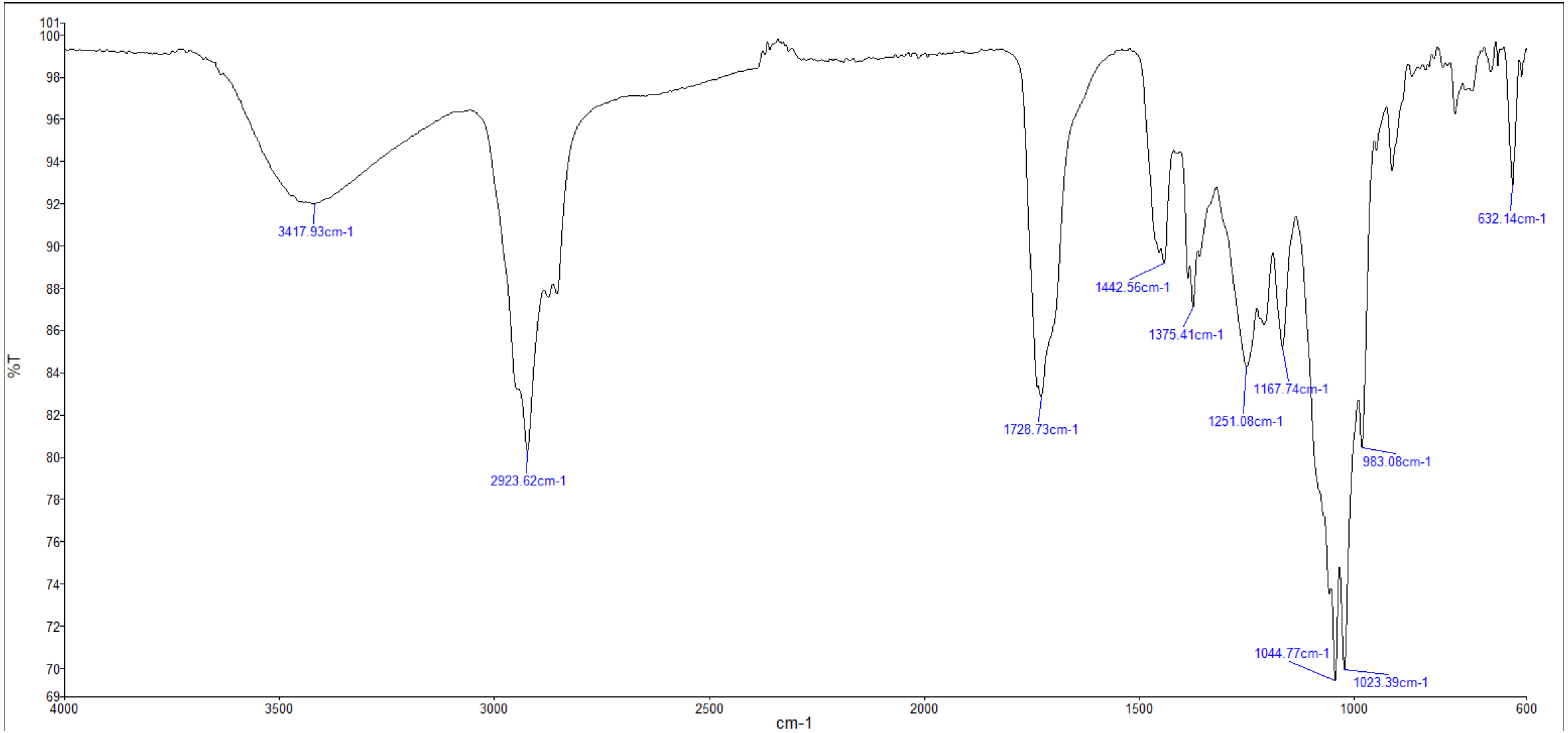

Supplement: Supplementary file 1 — Additional file 1. [file 13659_2025_499_MOESM1_ESM.pdf]
